# Derivation of the Time Dependent Gross–Pitaevskii Equation in Two Dimensions

**DOI:** 10.1007/s00220-019-03599-x

**Published:** 2019-11-08

**Authors:** Maximilian Jeblick, Nikolai Leopold, Peter Pickl

**Affiliations:** 1 Mathematisches Institut, Ludwig-Maximilians-Universität München, Theresienstraße 39, 80333 Munich, Germany; 2 Institute of Science and Technology Austria (IST Austria), Am Campus 1, 3400 Klosterneuburg, Austria; 3 Duke Kunshan University, Duke Avenue 8, Kunshan, 215316 China; 4 Ludwig-Maximilians-Universität München, Theresienstraße 39, 80333 Munich, Germany

## Abstract

We present microscopic derivations of the defocusing two-dimensional cubic nonlinear Schrödinger equation and the Gross–Pitaevskii equation starting from an interacting *N*-particle system of bosons. We consider the interaction potential to be given either by $W_\beta (x)=N^{-1+2 \beta }W(N^\beta x)$, for any $\beta >0$, or to be given by $V_N(x)=e^{2N} V(e^N x)$, for some spherical symmetric, nonnegative and compactly supported $W,V \in L^\infty ({\mathbb {R}}^2,{\mathbb {R}})$. In both cases we prove the convergence of the reduced density corresponding to the exact time evolution to the projector onto the solution of the corresponding nonlinear Schrödinger equation in trace norm. For the latter potential $V_N$ we show that it is crucial to take the microscopic structure of the condensate into account in order to obtain the correct dynamics.

## Introduction

We are interested in the time evolution of bosonic quantum systems of *N* particles in two dimensions that interact with each other by a two-particle interaction potential. At a given time *t*, the state of the system is described by a wave function $\Psi _t \in L^2_{s}({\mathbb {R}}^{2N}, {\mathbb {C}})$, where $L^2_{s}({\mathbb {R}}^{2N}, {\mathbb {C}})$ denotes the Hilbert space of all $\Psi \in L^2({\mathbb {R}}^{2N}, {\mathbb {C}})$ which are symmetric under permutations of the variables $x_1,\ldots , x_N \in {\mathbb {R}}^2$. The Hamiltonian of the system is given by

1 $$\begin{aligned} H_U=-\sum _{j=1}^N \Delta _j+\sum _{1\le j< k\le N} U(x_j-x_k) +\sum _{j=1}^N A_t(x_j) \end{aligned}$$

with $A_{\cdot }{:}\,{\mathbb {R}}^2 \times {\mathbb {R}} \rightarrow {\mathbb {R}}$ being a time-dependent external potential and $U{:}\,{\mathbb {R}}^2 \rightarrow {\mathbb {R}}$ modeling the interaction between the particles. The time evolution of the system is described by the Schrödinger equation

2 $$\begin{aligned} \text {i}\partial _t \Psi _t = H_U\Psi _t \end{aligned}$$

with initial datum $\Psi _0 \in L^2_{s}({\mathbb {R}}^{2N}, {\mathbb {C}})$. In general, even for small particle numbers *N*, it is not possible to solve the Schrödinger equation exactly or numerically. The time evolution of the system, however, can approximately be determined if one studies special classes of initial conditions and certain types of interaction potentials. In this paper, we are concerned with the dynamical evolution of a Bose–Einstein condensate. This state of matter appears if one cools bosons in an external trapping potential near absolute zero temperature such that almost all particles occupy the same quantum state (see e.g. [38] for a comprehensive discussion). After the trapping potential has been changed or completely switched off, the condensate is no longer in equilibrium and one would like to study its evolution in space.

Mathematically, the appearance of a Bose–Einstein condensate is described by means of the one-particle reduced density matrix $\gamma ^{(1)}_{\Psi }$ of the state $\Psi$. $\gamma ^{(1)}_{\Psi }$ is a non-negative trace class operator on $L^2({\mathbb {R}}^2,{\mathbb {C}})$ with an integral kernel given by

$$\begin{aligned} \gamma ^{(1)}_{\Psi }(x,x')=\int _{{\mathbb {R}}^{2N-2}} \overline{\Psi (x,x_2,\ldots ,x_N)} \Psi (x',x_2,\ldots ,x_N)d^2x_2\ldots d^2x_N. \end{aligned}$$

A state $\Psi$ is said to exhibit complete Bose–Einstein condensation, if there exists a one-particle wave function $\varphi \in L^2({\mathbb {R}}^2,{\mathbb {C}})$ with $\Vert \varphi \Vert =1$ such that $\gamma ^{(1)}_{\Psi } \rightarrow |\varphi \rangle \langle \varphi |$ in trace norm as $N \rightarrow \infty$.^1^ Initially, we consider a complete condensed state $\Psi _0$ and then show that $\gamma ^{(1)}_{\Psi _t} \rightarrow |\varphi _t\rangle \langle \varphi _t|$ as $N \rightarrow \infty$, where $\varphi _t$ solves a nonlinear Schrödinger equation. This statement shows that the condensate is stable during the time evolution. Moreover, it proves that the time-evolution of the one-particle reduced density matrix which is given by the many-body Schrödinger equation can approximately be described by a much simpler nonlinear one-particle equation.

To state the exact form of the one-particle equation, we specify the potentials *U* we are interested in.

- For $\beta >0$, we consider the so called nonlinear Schrödinger (NLS) scaling $U(x)= W_{\beta ,N}(x) = N^{-1+2 \beta } W(N^\beta x)$, for a compactly supported, spherically symmetric and nonnegative potential $W \in L^\infty _c ({\mathbb {R}}^2, {\mathbb {R}})$. In the case of $\beta > 1/2$, such a scaling models strong but short range repulsive interactions. The origin of the scaling can heuristically be motivated by the fact that for a completely factorized wave function $\Psi = \varphi ^{\otimes N}$ with $\varphi \in H^2({\mathbb {R}}^2, {\mathbb {C}})$ the kinetic energy per particle^2^$\frac{1}{N} \langle \!\langle \Psi , \sum _{k=1}^N (- \Delta _k) \Psi \rangle \!\rangle = - \langle \varphi , \Delta \varphi \rangle = {\mathcal {O}}(1)$ is of the same order as the potential energy per particle $\frac{1}{N} \langle \!\langle \Psi , \sum _{1< j< k < N}^N W_\beta (x_j -x_k)\Psi \rangle \!\rangle = {\mathcal {O}}(1)$.
- We also consider exponentially scaled potentials $$U(x) = V_N(x)=e^{2N} V(e^N x) $$ with $$V\in L^\infty _c ({\mathbb {R}}^2, {\mathbb {R}}) $$ being spherically symmetric and nonnegative. This scaling will be denoted Gross–Pitaevskii scaling in the following. The motivation to consider an exponential scaling is similar to the Gross Pitaevskii scaling $$V_N(x)=N^2 V(Nx)$$ in three space dimensions. Namely, the kinetic and interaction energy are of the same order for a gas of fixed volume. This will be shown below, when discussing the scattering process of two particles, see (4). Furthermore, the interaction originates from a *N*-independent potential by rescaling space and time coordinates [see (7)]. Our results can be generalized to a wider class of *N*-dependent interactions covering most of the relevant cases discussed in the literature on two dimensional Bose gases [39].

For these scalings the condensate wave function $\varphi _t$ satisfies the cubic nonlinear Schrödinger equation

3 $$\begin{aligned} \text {i}\partial _t \varphi _t=\left( -\Delta +A_t\right) \varphi _t+ b_U |\varphi _t|^2\varphi _t=:h_{ b_U}^{GP}\varphi _t \end{aligned}$$

with initial datum $\varphi _0$. The precise definition of $b_U$ will be given in Definition 2.1. At the moment however if suffices to note that for the potentials from above we have $b_{W_{\beta ,N}}= N\Vert W_{\beta ,N} \Vert _1= \Vert W\Vert _1$ if $U=W_{\beta ,N}$ and $b_{V_N}= 4\pi$ for $U= V_N$. In case that the coupling constant is given by $b_{V_N}= 4\pi$ Eq. (3) is also referred to as Gross–Pitaevskii equation.

We are going to explain on a heuristic level why the coupling constants differ in the NLS and Gross–Pitaevskii scaling. We first consider the exponential scaling and assume that the energy of the many-body state $\Psi _t$ is comparable to the ground state energy of the system. In this case, the wave function develops a short scale correlation structure which prevents the particles from being too close to each other [39]. If we neglect for the moment all but two particle correlations, one may heuristically think of $\Psi _t$ to be of Jastrow-type [38, p. 15 and p. 28], i.e. $\Psi _t (x_1, \dots ,x_N) \approx \prod _{i<j} F(x_i-x_j) \prod _{k=1}^ N \varphi _t (x_k)$. The function *F* accounts for pair correlations between the particles at scales of order ${\mathcal {O}}(e^{-N})$. These correlations determine the time evolution of the condensate in a crucial manner and must therefore explicitly be taken into account. Since $V_N$ is a strong, short range potential, the interaction between the particles can in first order be described as a two-body scattering process. That is, the correlation function *F* should approximately be given by the zero energy scattering state $j_{N,R} \in C^1({\mathbb {R}}^2,{\mathbb {R}})$ which is defined by

4 $$\begin{aligned} {\left\{ \begin{array}{ll} \left( - \Delta _x + \frac{1}{2} e^{2N} V(e^N x) \right) j_{N,R}(x)=0, \\ j_{N,R}(x)=1 \; \text {for } |x| = R \end{array}\right. } \end{aligned}$$

for some $R \in (0, \infty )$ used to normalize $j_{N,R}$ via the second line of (4). Note, that it is a peculiarity of two dimensional scattering states that $\lim _{x\rightarrow \infty }|j_{N,R}(x)|$ does not exist for short range potentials and can not be used for normalization. A particle at location *x* then experiences the effective interaction

$$\begin{aligned} \int _{{\mathbb {R}}^2} d^2y N V_N( x-y)j_{N,R}(x-y) |\varphi _t(y)|^2 \approx | \varphi _t(x) |^2 \int _{{\mathbb {R}}^2} d^2x NV_N( x)j_{N,R}(x), \end{aligned}$$

see e.g. [19] for a nice derivation. It will be shown in Sect. 5 that

$$\begin{aligned} N\int _{{\mathbb {R}}^2} d^2x V_N( x)j_{N,R}(x) = N \frac{4\pi }{ \ln \left( \frac{R}{ae^{-N}} \right) }, \end{aligned}$$

where *a* denotes the scattering length of the potential *V*. Since $\frac{4\pi }{ \ln \left( \frac{R}{ae^{-N}} \right) } \approx \frac{4 \pi }{N}$ holds for $a>0$, the effective coupling $b_{V_N}$ will be given by $4 \pi$. This shows that the scaling we used gives us a system where the kinetic energy and the interaction energy are of the same order.

Let us now turn to the NLS scaling and consider for $\beta > 0$ the scattering equation of the potential $W_{\beta ,N}$

5 $$\begin{aligned} {\left\{ \begin{array}{ll} \left( - \Delta _x + N^{-1+2\beta } W(N^{\beta } x) \right) F_{N,\beta , R}(x)=0, \\ F_{N,\beta , R}(x)=1 \; \text {for } |x| = R. \end{array}\right. } \end{aligned}$$

With $y= N^{\beta } x$, ${\tilde{R}}= N^{\beta } R$ and $G_{N,\beta , R} = F_{N,\beta , R}(N^{-\beta } \cdot )$, this can be written as

6 $$\begin{aligned} {\left\{ \begin{array}{ll} \left( - \Delta _y + N^{-1} W(y) \right) G_{N,\beta , R}(y)=0, \\ G_{N,\beta , R}(y)=1 \; \text {for } |y| = {\tilde{R}}. \end{array}\right. } \end{aligned}$$

Due to the factor $N^{-1}$, the zero energy scattering state is almost constant for large *N*, $F_{N,\beta , R}(x) \approx 1 \; \forall \; |x| \le R$. It can therefore be concluded that the microscopic structure has a negligible effect on the effective interaction on each particle which is approximated by^3^

$$\begin{aligned}&\int _{{\mathbb {R}}^2} d^{2}y {NW}_{\beta ,N}(x-y)F_{N,\beta ,R}(x-y) |\varphi _t(y)|^2 \approx \int _{{\mathbb {R}}^2} d^{2}y {NW}_{\beta ,N}(x-y) |\varphi _t(y)|^2 \\&\quad \rightarrow \Vert W\Vert _1 | \varphi _t(x)|^{2}. \end{aligned}$$

This yields to the correct coupling in the effective equation (3) in the case of $U(x)= W_{\beta ,N}(x)$.

Let us briefly compare the phenomenon of Bose–Einstein condensation in two and three dimensions. In three dimension the NLS scaling is defined by $N^{-1+ 3 \beta } W(N^{\beta }x)$ only for $0<\beta < 1$ while in the case of $\beta =1$ the microscopic structure must be taken into account. This difference originates from the different form of the scattering state in two and three dimension, see Appendix C of [38]. In the case that the time evolution of $\Psi _t$ is generated by $H_{V_N}$ it is interesting to note that the effective evolution equation of $\varphi _t$ does not depend on the scattering length *a*. Also this contrasts the three-dimensional case, where the correct mean field coupling is given by $8 \pi a_{3D}$, $a_{3D}$ denoting the scattering length of the potential in three dimensions. The universal coupling $4 \pi$ in the case of a two-dimensional setup is known within the physical literature, see e.g. (30) and (A3) in [18] (note that $\hbar =1, m= \frac{1}{2}$ in our choice of coordinates).

Actually, our dynamical result complements a more general theory describing the ground state properties of dilute, two-dimensional Bose gases. It was shown in [39] that for a gas with repulsive interaction $V \ge 0$, the ground state energy per particle is to leading order given by either the Gross–Pitaevskii energy functional with coupling parameter $8 \pi /| \ln ( {\overline{\rho }} a^ 2 )|$ or a Thomas–Fermi type functional, depending on the diluteness of the gas, i.e. the mean-particle distance compared to the scattering length of the interaction. Here, ${\overline{\rho }}$ denotes the mean density of the gas and *a* is the scattering length which must decrease exponentially with *N* in the Gross–Pitaevskii limit [39, p. 20].

It should be pointed out that there has been some debate about the question whether two-dimensional Bose–Einstein condensation can be observed experimentally. This amounts to the question whether condensation takes place for temperatures $T>0$. For an ideal, noninteracting gas in a box, the standard grand canonical computation for the critical temperature $T_c$ of a Bose–Einstein condensate shows that there is no condensation for $T>0$. For trapped, noninteracting bosons in a confining power-law potential, the findings in [3] however show that in that case $T_c>0$ holds. Finally, it was proven in [37] that $\gamma ^{(1)}_{\Psi }$ converges to $|\varphi \rangle \langle \varphi |$ in trace norm if $\Psi$ is the ground state of $H_{V_N}$ and $\varphi$ is the ground state of the Gross–Pitaevskii energy functional, see (8). It was furthermore proven that one does not observe 100% condensation in the ground state of an interacting homogenous system. The emergence of 100% Bose–Einstein condensation as a ground state phenomenon thus highly depends on the particular physical system. Our approach is the following: Initially, we assume the convergence of $\gamma ^{(1)}_{\Psi _0}$ to $|\varphi _0\rangle \langle \varphi _0|$. We then show the persistence of condensation for time scales of order one. Our assumption is thus in agreement with the findings in [37].

The rigorous derivation of effective evolution equations is well known in the literature, see e.g. [2, 5, 9–11, 19–22, 30, 43, 44, 48–51] and references therein. For the two-dimensional case we consider, it has been proven, for $0<\beta <3/4$ and *W* nonnegative, that $\gamma ^{(1)}_{\Psi _t}$ converges to $|\varphi _t\rangle \langle \varphi _t|$ as $N \rightarrow \infty$ [27]. For $0< \beta < 1/6$, it has been established in [14] that the reduced density matrices converge, assuming that the potential *W* is attractive, i.e. $W \le 0$. This result was later extended to a larger class of scaling parameters $\beta$, under some assumptions on the negative part of the potential *W* [26, 34]. In [45] a norm approximation to the two-dimensional focusing Schrödinger equation in the NLS scaling with $0<\beta <1$ was considered. Here, the evolution of the condensate is effectively described by the nonlinear Schrödinger equation while the evolution of the fluctuations around the condensate is governed by a quadratic Hamiltonian, resulting from Bogoliubov approximation. Another approach which relates more closely to the experimental setup is to consider a three-dimensional gas of bosons which is strongly confined in one spatial dimension. Then, one obtains an effective two-dimensional system in the unconfined directions. We remark that in this dimensional reduction two limits appear, the length scale in the confined direction and the scaling of the interaction in the unconfined directions. A derivation of the two-dimensional Gross–Pitaevskii equation from the three-dimensional quantum many-body dynamics of strongly confined bosons was just recently given in [7]. Further results in this direction can be found in [4, 6, 8, 15–17, 28, 29]. For known results regarding the ground state properties of dilute Bose gases, we refer to the monograph [38], which also summarizes the papers [37, 39, 40].

Our proof is based on [49], which covers the derivation of the time dependent Gross–Pitaevskii equation in three dimensions. In particular, the exponential scaling of the interaction forces us to adapt crucial ideas and refine many estimates. Additional difficulties arise amongst others from the logarithmic behaviour of the scattering state and the fact that $\Vert e^{2N} V(e^N \cdot ) \Vert _{L^1({\mathbb {R}}^2,{\mathbb {C}})} \sim 1$ while $\Vert N^{-1+ 3} V(N \cdot ) \Vert _{L^1({\mathbb {R}}^3,{\mathbb {C}})} \sim N^{-1}$ in the three-dimensional Gross–Pitaevskii regime.

We shortly discuss the physical relevance of the Gross–Pitaevskii scaling. It is possible to rescale space- and time-coordinates in such a way that in the new coordinates the interaction is *notN*-dependent. Choosing $y=e^Nx$ and $\tau = e^{2N}t$ the Schrödinger equation reads

7 $$\begin{aligned} \text {i}\frac{d}{d\tau } \Psi _{e^{-2N}\tau }=\left( -\sum _{j=1}^N \Delta _{y_j}+\sum _{1\le j< k\le N} V(y_j-y_k) +\sum _{j=1}^N A_{e^{-2N}\tau }(e^{-N}y_j)\right) \Psi _{e^{-2N}\tau }.\nonumber \\ \end{aligned}$$

The latter equation thus corresponds to an extremely dilute gas of bosons with density $\sim e^{-2N}$. In order to observe a nontrivial dynamics, this condensate is then monitored over time scales of order $\tau \sim e^{2N}$. Since the trapping potential is adjusted according to the density of the gas in the experiment, the *N* dependence of $A_{e^{-2N}\tau }(e^{-N} \cdot )$ is reasonable.

## Main Result

Our main theorem consists of two parts, which consider potentials in the NLS and Gross–Pitaevskii scaling, respectively. For the proof of the theorem it is useful to enlarge the class of potentials in the NLS regime because it allows us in the derivation of the Gross–Pitaevskii equation to refer to various estimates that appear in first part of the proof.

### Definition 2.1

- For $\beta >0$, we define the following space of sequences $\big ( W_{\beta ,N} \big )_{N \in {\mathbb {N}}}$.
  $$\begin{aligned} \widetilde{{\mathcal {W}}}_{\beta }&= \Big \lbrace \big ( W_{\beta ,N} \big )_{N \in {\mathbb {N}}}|\;\; W_{\beta ,N} \in L_{c}^{\infty }({\mathbb {R}}^2,{\mathbb {R}}), \exists C>0 \, \text { independent of}\\&\qquad \, N \, \text {and} \, \beta {:}\,W_{\beta ,N}(x) \ge 0 \,\, \forall x \in {\mathbb {R}}^2, \\&\qquad \Vert W_{\beta ,N} \Vert _1 \le { CN }^{-1}, \Vert W_{\beta ,N} \Vert \le { CN }^{-1+ \beta }, \Vert W_{\beta ,N} \Vert _\infty \le { CN }^{-1+2 \beta }, \\&\qquad W_{\beta ,N} (x)=0 \; \forall |x| \ge { CN }^{-\beta },\; W_{\beta ,N} \text { is spherically symmetric} \Big \rbrace . \end{aligned}$$
- For every $\big ( W_{\beta ,N} \big )_{N \in {\mathbb {N}}} \in \widetilde{{\mathcal {W}}}_{\beta }$ we define the coupling parameter $b_{W_{\beta }} = \lim _{N \rightarrow \infty } N ||W_{\beta ,N} ||_1$.
- Define the set of potentials ${\mathcal {W}}_{\beta }$ by
  $$\begin{aligned} {\mathcal {W}}_{\beta }&= \Big \lbrace \big ( W_{\beta ,N} \big )_{N \in {\mathbb {N}}} \in \widetilde{{\mathcal {W}}}_{\beta } |\;\; \exists C>0 \, \text { independent of} \, N \, \text {and} \, \beta {:}\,\\&\qquad \big | N ||W_{\beta ,N} ||_1 - b_{W_{\beta }} \big | \le C N^{-1} \ln (N) \Big \rbrace . \end{aligned}$$

To ease the notation, we often omit to display the dependence on *N* and denote both the sequence $\big ( W_{\beta ,N} \big )_{N \in {\mathbb {N}}}$ and the element $W_{\beta ,N}$ by $W_{\beta }$.

### Remark 2.2

It should be noted that $N^{-1+2\beta }W(N^\beta x) \in {\mathcal {W}}_\beta$, if $W \in L^\infty _c ({\mathbb {R}}^2, {\mathbb {R}})$ is nonnegative and spherically symmetric. In this case, $b_{W_{\beta }} = ||W||_1$.

For notational convenience, it is in addition helpful to define a class of potentials with Gross–Pitaevskii scaling.

### Definition 2.3

Define the set of sequences of potentials $\big (V_N \big )_{N \in {\mathbb {N}}}$ as

$$\begin{aligned} {\mathcal {V}}_N&= \lbrace \big (V_N \big )_{N \in {\mathbb {N}}} | \exists V \in L^\infty _c ({\mathbb {R}}^2,{\mathbb {R}}) \, \text {not being identically zero}{:}\,V_N(x)= e^{2N} V(e^N x), \\&\qquad V(x)\ge 0 \; \forall x \in {\mathbb {R}}^2, V\text { is spherically symmetric} \rbrace . \end{aligned}$$

With a slight abuse of notation we use $V_N$ to denote the sequence $\big (V_N \big )_{N \in {\mathbb {N}}}$ and its *N*th element.

For $U \in \lbrace W_{\beta }, V_N \rbrace$ and $A_t \in L^{\infty }({\mathbb {R}}^2, {\mathbb {R}})$, define the energy functional ${\mathcal {E}}_{U}{:}\,H^1({\mathbb {R}}^{2N}, {\mathbb {C}}) \rightarrow {\mathbb {R}}$

$$\begin{aligned} {\mathcal {E}}_{U}(\Psi )=N^{-1}\langle \!\langle \Psi ,H_{U}\Psi \rangle \!\rangle , \end{aligned}$$

where $\langle \!\langle \cdot ,\cdot \rangle \!\rangle$ denotes the scalar product on $L^2({\mathbb {R}}^{2N},{\mathbb {C}})$. Furthermore, define the Gross–Pitaevskii energy functional ${\mathcal {E}}_{b_U}^{GP}{:}\,H^1({\mathbb {R}}^ {2}, {\mathbb {C}}) \rightarrow {\mathbb {R}}$

8 $$\begin{aligned} {\mathcal {E}}_{b_U}^{GP}(\varphi )&=\langle \nabla \varphi ,\nabla \varphi \rangle +\langle \varphi ,(A_t+\frac{1}{2} b_U|\varphi |^2)\varphi \rangle =\langle \varphi , (h_{b_U}^{GP}-\frac{1}{2} b_U |\varphi |^2)\varphi \rangle \end{aligned}$$

where $\langle \cdot ,\cdot \rangle$ denotes the scalar product on $L^2({\mathbb {R}}^2,{\mathbb {C}})$. Note that both ${\mathcal {E}}_{U}(\Psi )$ and ${\mathcal {E}}_{b_U}^{GP}(\varphi )$ depend on *t*, due to the time varying external potential $A_t$. For the sake of readability, we will not indicate this time dependence explicitly. Our main theorem is the following.

### Theorem 2.4

Let $\Psi _0 \in L^2_{s}({\mathbb {R}}^{2N}, {\mathbb {C}}) \cap H^2({\mathbb {R}}^{2N}, {\mathbb {C}})$ with $\Vert \Psi _0\Vert =1$. Let $\varphi _0 \in L^{2}({\mathbb {R}}^2,{\mathbb {C}})$ with $\Vert \varphi _0\Vert =1$. Let the external potential $A_t$ satisfy $A_{\cdot } \in C^1({\mathbb {R}},L^{\infty }({\mathbb {R}}^2,{\mathbb {R}}))$.

- Let $$\beta >0$$, $$W_\beta \in {\mathcal {W}}_\beta $$ and let $$\Psi _t$$ the unique solution to $$i \partial _t \Psi _t = H_{W_\beta } \Psi _t$$ with initial datum $$\Psi _0$$. Let $$\varphi _t$$ the unique solution to $$i \partial _t \varphi _t = h^{GP}_{ b_{W_{\beta }}} \varphi _t$$ with initial datum $$\varphi _0$$ and assume that $$\varphi _t \in H^3({\mathbb {R}}^2, {\mathbb {C}})$$$$\forall t \in {\mathbb {R}}$$. Let $$ {\mathcal {E}}_{W_\beta }(\Psi _0) \le C $$, where $$C>0$$ is a constant independent of *N*. Then, for any $$t>0$$ there exists a constant $$0<C_t<\infty $$, which depends on *t* but not on *N*, such that
  9 $$\begin{aligned} \text {Tr} \left| \gamma ^{(1)}_{\Psi _t} - |\varphi _t \rangle \langle \varphi _t| \right|&\le e^{C_t} \Bigg ( \root 4 \of {\text {Tr} \left| \gamma ^{(1)}_{\Psi _0} - |\varphi _0 \rangle \langle \varphi _0| \right| } \nonumber \\&\quad +\,\sqrt{\left| {\mathcal {E}}_{W_\beta }(\Psi _0) -{\mathcal {E}}_{b_{W_{\beta }}}^{GP}(\varphi _0)\right| } + N^{-\gamma } \sqrt{\ln (N)} \Bigg ), \end{aligned}$$
  10 $$\begin{aligned} \left| {\mathcal {E}}_{W_\beta }(\Psi _t)-{\mathcal {E}}_{b_{W_{\beta }}}^{GP}(\varphi _t)\right|&\le e^{C_t} \Bigg ( \sqrt{\text {Tr} \left| \gamma ^{(1)}_{\Psi _0} - |\varphi _0 \rangle \langle \varphi _0| \right| } \nonumber \\&\quad +\,\left| {\mathcal {E}}_{W_\beta }(\Psi _0)-{\mathcal {E}}_{b_{W_{\beta }}}^{GP}(\varphi _0)\right| + N^{- 2\gamma } \ln (N) \Bigg ), \end{aligned}$$
  where $\gamma = \beta$ for $0<\beta <1/12$ and $\gamma = 1/20$ for $\beta \ge 1/12$.
- Let $$V_N \in {\mathcal {V}}_N$$ and let $$\Psi _t$$ the unique solution to $$i \partial _t \Psi _t = H_{V_N} \Psi _t$$ with initial datum $$\Psi _0$$. Let $$\varphi _t$$ the unique solution to $$i \partial _t \varphi _t = h^{GP}_{4 \pi } \varphi _t$$ with initial datum $$\varphi _0$$ and assume that $$\varphi _t \in H^3({\mathbb {R}}^2, {\mathbb {C}})$$$$\forall t \in {\mathbb {R}}$$. Let $$ {\mathcal {E}}_{V_N}(\Psi _0) \le C $$, where $$C>0$$ is a constant independent of *N*. Then, for any $$t>0$$ there exists a constant $$0<C_t<\infty $$, which depends on *t* but not on *N*, such that
  11 $$\begin{aligned} \text {Tr} \left| \gamma ^{(1)}_{\Psi _t} - |\varphi _t \rangle \langle \varphi _t| \right|&\le e^{C_t} \Bigg ( \root 4 \of {\text {Tr} \left| \gamma ^{(1)}_{\Psi _0} - |\varphi _0 \rangle \langle \varphi _0| \right| } \nonumber \\&\quad +\,\sqrt{\left| {\mathcal {E}}_{V_N} (\Psi _0) - {\mathcal {E}}^{GP}_{4 \pi } (\varphi _0) \right| } + N^{-1/20} \Bigg ), \end{aligned}$$
  12 $$\begin{aligned} \left| {\mathcal {E}}_{V_N} (\Psi _t) - {\mathcal {E}}^{GP}_{4 \pi } (\varphi _t) \right|&\le e^{C_t} \Bigg ( \sqrt{\text {Tr} \left| \gamma ^{(1)}_{\Psi _0} - |\varphi _0 \rangle \langle \varphi _0| \right| } \nonumber \\&\quad +\,\left| {\mathcal {E}}_{V_N} (\Psi _0) - {\mathcal {E}}^{GP}_{4 \pi } (\varphi _0) \right| + N^{-1/10} \Bigg ). \end{aligned}$$

### Remarks

- If one considers initial many-body states which exhibit condensation and whose energy per particle converges to the corresponding Gross–Pitaevskii energy, i.e.
  $$\begin{aligned}&\lim _{N \rightarrow \infty } \text {Tr} \left| \gamma ^{(1)}_{\Psi _0} - |\varphi _0 \rangle \langle \varphi _0| \right| = 0 \quad \text {and} \; \lim _{N \rightarrow \infty } \left| {\mathcal {E}}_{U} (\Psi _0) - {\mathcal {E}}^{GP}_{b_{U}} (\varphi _0) \right| = 0 \\&\quad \quad \text {with }U \in \{ W_{\beta }, V_N \}, \end{aligned}$$
  it follows from Theorem 2.4 that
  $$\begin{aligned} \lim _{N \rightarrow \infty } \text {Tr} \left| \gamma ^{(1)}_{\Psi _t} - |\varphi _t \rangle \langle \varphi _t| \right| = 0 \quad \text {and} \; \lim _{N \rightarrow \infty } \left| {\mathcal {E}}_{U} (\Psi _t) - {\mathcal {E}}^{GP}_{b_{U}} (\varphi _t) \right| = 0 \quad \text {for any t > 0.} \end{aligned}$$
  Our result consequently shows the stability of the condensate during the time evolution.
- It has been shown that in the limit $N\rightarrow \infty$ the energy-difference ${\mathcal {E}}_{V_N}(\Psi ^{gs})-{\mathcal {E}}_{4 \pi }^{GP}(\varphi ^{gs})\rightarrow 0$, where $\Psi ^{gs}$ is the ground state of a trapped Bose gas and $\varphi ^{gs}$ the ground state of the respective Gross–Pitaevskii energy functional, see [39, 40].
- The necessity to require $\varphi _t \in H^3({\mathbb {R}}^2, {\mathbb {C}})$ stems from the fact that the constant $C_t$ in (9) and (11) depends on $\Vert \varphi _t \Vert _{H^3}$, see the discussion before Lemma 4.7. For regular enough external potentials $A_t$ we expect the assumption $\varphi _t \in H^3({\mathbb {R}}^2, {\mathbb {C}})$ to follow from regularity assumptions on the initial datum $\varphi _0$. If $\varphi _0 \in \Sigma ^3({\mathbb {R}}^2,{\mathbb {C}}) = \lbrace f \in L^2 ({\mathbb {R}}^2, {\mathbb {C}})| \sum _{ \alpha + \beta \le 3} \Vert x^\alpha \partial _x^\beta f \Vert < \infty \rbrace$ holds, the bound $\Vert \varphi _t \Vert _{H^3} < \infty$ has been proven for external potentials which are at most quadratic in space, see [13] and Lemma 4.7. In particular, for $\varphi _0 \in \Sigma ^3({\mathbb {R}}^2, {\mathbb {C}})$, the bound $\Vert \varphi _t \Vert _{H^3} \le C$ with $C>0$ uniformly bounded in *t* holds if the external potential is not present, i.e. $A_t=0$ [see [13] above (1.3.)].
- One can relax the conditions on the initial condition and only require $\Psi _0 \in L^2_{s}({\mathbb {R}}^{2N}, {\mathbb {C}})$ using a standard density argument.

## Organization of the Proof

The method we are applying to prove Theorem 2.4 was originally introduced in [50] and later generalized to derive various mean-field equations  [1, 8, 30, 32, 33, 42, 46–49]. Our proof is primarily based on [49] which covers the three-dimensional counterpart of our system. The key idea of the method is to show the existence of Bose–Einstein condensation not in terms of reduced density matrices but to consider an equivalent measure of condensation. Heuristically speaking, we count for each time *t* the relative number of those particles which are not in the state of the condensate wave function $\varphi _t$. It is then possible to show that the rate of the particles which leave the condensate is small, if initially almost all particles were in the state $\varphi _0$. The counting of the particles will be performed with the help of a functional. In order to define it, we introduce the following operators.

### Definition 3.1

Let $$\varphi \in L^{2}({\mathbb {R}}^2,{\mathbb {C}})$$ with $$\Vert \varphi \Vert =1$$.

- For any $1\le j\le N$ the projectors $p_j^\varphi {:}\,L^2({\mathbb {R}}^{2N},{\mathbb {C}})\rightarrow L^2({\mathbb {R}}^{2N},{\mathbb {C}})$ and $q_j^\varphi {:}L^2({\mathbb {R}}^{2N},{\mathbb {C}})\rightarrow L^2({\mathbb {R}}^{2N},{\mathbb {C}})$ are defined as
  $$\begin{aligned} p_j^\varphi \Psi =\varphi (x_j)\int \varphi ^*({\tilde{x}}_j)\Psi (x_1,\ldots , {\tilde{x}}_j,\dots ,x_N)d^2{\tilde{x}}_j\;\;\;\forall \;\Psi \in L^2({\mathbb {R}}^{2N},{\mathbb {C}})\end{aligned}$$
  and $q_j^\varphi =1-p_j^\varphi$. We shall also use, with a slight abuse of notation, the bra-ket notation $p_j^\varphi =|\varphi (x_j)\rangle \langle \varphi (x_j)|$.
- For any $0\le k\le N$ we define the set
  $$\begin{aligned} {\mathcal {S}}_k=\left\{ \vec {s} = (s_1,s_2,\ldots ,s_N)\in \{0,1\}^N\;;\; \sum _{j=1}^N s_j=k\right\} \end{aligned}$$
  and the orthogonal projector $P_{k}^\varphi {:}\,L^2({\mathbb {R}}^{2N},{\mathbb {C}})\rightarrow L^2({\mathbb {R}}^{2N},{\mathbb {C}})$ as
  $$\begin{aligned} P_{k}^\varphi =\sum _{\vec s \in {\mathcal {S}}_k}\prod _{j=1}^N\big (p_{j}^{\varphi }\big )^{1-s_j} \big (q_{j}^{\varphi }\big )^{s_j}. \end{aligned}$$
  For negative *k* and $k>N$ we set $P_{k}^\varphi =0$.
- For any function $m{:}\,{\mathbb {N}}_0 \rightarrow {\mathbb {R}}^+_0$ we define the operator ${\widehat{m}}^{\varphi }{:}\,L^2({\mathbb {R}}^{2N},{\mathbb {C}})\rightarrow L^2({\mathbb {R}}^{2N},{\mathbb {C}})$ as
  13 $$\begin{aligned} {\widehat{m}}^{\varphi }=\sum _{j=0}^N m(j)P_j^\varphi . \end{aligned}$$
  We also need the shifted operators ${\widehat{m}}^{\varphi }_d{:}\,L^2({\mathbb {R}}^{2N},{\mathbb {C}})\rightarrow L^2({\mathbb {R}}^{2N},{\mathbb {C}})$ given by
  $$\begin{aligned} {\widehat{m}}^{\varphi }_d=\sum _{j=-d}^{N-d} m(j+d)P_j^\varphi \quad \text {with} \; d \in {\mathbb {Z}}. \end{aligned}$$

Following a general strategy^4^ we will define a functional $\alpha {:}\,L^2({\mathbb {R}}^{2N},{\mathbb {C}})\times L^2({\mathbb {R}}^2,{\mathbb {C}})\rightarrow {\mathbb {R}}^+_0$ such that

- $\alpha (\Psi _0,\varphi _0)\rightarrow 0$ for suitably chosen initial data $(\Psi _0,\varphi _0) \in L^2({\mathbb {R}}^{2N},{\mathbb {C}})\times L^2({\mathbb {R}}^2,{\mathbb {C}})$.^5^
- If $\Psi _t$ is a solution of (2) and $\varphi _t$ a solution of (3), $\alpha (\Psi _t,\varphi _t)$ can be estimated by $\alpha (\Psi _0,\varphi _0)+ \int _0^t ds \, C_s \big ( \alpha (\Psi _s,\varphi _s) + {\mathcal {O}}(1) \big )$ for some time dependent constant $C_s$. Using a Grönwall type estimate, it then follows that $\alpha (\Psi _t,\varphi _t) \le e^{ 2 \int _0^t {{d}}\tau \, C_\tau } \big ( \alpha (\Psi _0,\varphi _0) + {\mathcal {O}}(1) \big )$.
- $\alpha (\Psi _t,\varphi _t)\rightarrow 0$ implies the convergence of the one-particle reduced density matrix of $\Psi _t$ to $|\varphi _t\rangle \langle \varphi _t|$ in trace norm as well as the convergence of the energy per particle of the many-body system to the energy of the condensate wave function.

In [30, 50] the mean field scaling $W_0(x) = N^{-1} W(x)$ and a condensate wave function which evolves according to the Hartree equation $i \partial _t \varphi _t = \big ( - \Delta + A_t \big ) \varphi _t + \big ( W *|\varphi _t|^2 \big ) \varphi _t$ were considered in the three-dimensional setting. In these works it was shown that the persistence of condensation can be proven if one chooses

$$\begin{aligned} \alpha (\Psi _t,\varphi _t)= \left\langle \!\left\langle \Psi _t, \left( {\widehat{n}}^{\varphi _t}\right) ^j\Psi _t \right\rangle \!\right\rangle , \end{aligned}$$

where $n(k)=\sqrt{k/N}$, $j >0$ and $\Psi _t$ is a solution of (2) with $U = W_0$. The choice $j=2$ corresponds to the functional $\langle \!\langle \Psi _t, \sum _{k=0}^N \frac{k}{N} P_{k}^{\varphi _t} \Psi _t \rangle \!\rangle$, whose action on $\Psi _t$ can be viewed as "counting the relative number of particles which are not in the state $\varphi _t$". Other values of *j* or a different choice of ${\widehat{m}}^{\varphi _t}$ should be understood as a weighted measure of counting the number of particles which are not in the condensate state. We will therefore sometimes call *m* the weight function of the functional $\alpha$.

In this work we are interest in interaction potentials which get peaked as $N \rightarrow \infty$. As explained in Sect. 6.1, it is then no longer possible to obtain a Grönwall estimate with the previous choice of the functional and we have to adjust it in accordance with the scaling of the interaction. The precise definition of the functional and the proof of Theorem 2.4 are given in Sect. 6. In the preceding chapters we introduce the necessary preliminaries.

The rest of the paper is organized as follows:

- In Sect. 4 we start by fixing the notation. Afterwards, we recall important properties of the operator ${\widehat{m}}$ and explain the required regularity conditions on the solutions of the nonlinear Schrödinger equation.
- In case of the exponential scaling, the interaction is so strong such that the many-body state develops a short scale correlation structure. This correlation structure affects the time evolution of the condensate and must therefore also be regarded in the definition of the functional. In Sect. 5, we explain the correlations structure in greater detail, provide certain estimates on the zero-energy scattering state and explain how the effective coupling parameter $b_{V_N}$ can be inferred from the microscopic structure.
- In Sect. 6 we prove Theorem 2.4. We first consider the potential $W_\beta$ and define a counting measure which allows us to establish a Grönwall estimate for all $\beta >0$. We will explain in detail how one arrives at this Grönwall estimate. Afterwards, the counting measure is adjusted to the case $V_N$, taking the microscopic structure $j_{N,R}$ of the wave function into account. We then establish a Grönwall estimate and finally prove the second part of the main theorem.
- In order to improve the readability of the paper we only state the estimates which are needed for the proof of Theorem 2.4 in Sect. 6. Their derivation is provided afterwards in Sect. 7.

## Preliminaries

We will first fix the notation we are going to employ during the rest of the paper.

### Notation 4.1

- Throughout the paper hats $\;{\widehat{\cdot }}$  will always be used in the sense of Definition 3.1(c). The label *n* will always be used for the function $n(k)=\sqrt{k/N}$.
- For better readability, we will often omit the upper index $\varphi$ on $p_j$, $q_j$, $P_j$ and ${\widehat{\cdot }}$. It will be placed exclusively in formulas where the $\varphi$-dependence is crucial.
- The operator norm, defined for any linear operator $f{:}\,L^2({\mathbb {R}}^{2N},{\mathbb {C}})\rightarrow L^2({\mathbb {R}}^{2N},{\mathbb {C}})$, will be denoted by
  $$\begin{aligned} \Vert f\Vert _{\text {op}}=\sup _{\psi \in L^2({\mathbb {R}}^{2N},{\mathbb {C}}), \Vert \Psi \Vert =1}\Vert f\Psi \Vert . \end{aligned}$$
- We will bound expressions which are uniformly bounded in *N* and *t* by some constant *C*. Constants appearing in a sequence of estimates will not be distinguished, i.e. in $X\le CY\le CZ$ the constants may differ.
- We will denote by ${\mathcal {K}}(\varphi _t, A_t)$ a generic polynomial with finite degree in $\Vert \varphi _t\Vert _\infty , \Vert \nabla \varphi _t\Vert _\infty , \Vert \nabla \varphi _t\Vert , \Vert \Delta \varphi _t \Vert , \Vert A_t\Vert _\infty , \int _0^t ds \Vert \dot{A}_s \Vert _\infty$ and $\Vert \dot{A}_t \Vert _\infty$. Note, in particular, that for a generic constant *C* the inequality $C \le {\mathcal {K}}(\varphi _t, A_t)$ holds. The exact form of ${\mathcal {K}}(\varphi _t, A_t)$ which appears in the final bounds can be reconstructed, collecting all contributions from the different estimates.
- We will denote for any multiplication operator $F{:}\,L^2({\mathbb {R}}^2, {\mathbb {C}}) \rightarrow L^2({\mathbb {R}}^2, {\mathbb {C}})$ the corresponding operator
  $$\begin{aligned} \mathbb {1}^{\otimes (k-1)} \otimes F \otimes \mathbb {1}^{\otimes (N-k)}{:}\,L^2({\mathbb {R}}^{2N}, {\mathbb {C}}) \rightarrow L^2({\mathbb {R}}^{2N}, {\mathbb {C}}) \end{aligned}$$
  acting on the *N*-particle Hilbert space by $F(x_k)$. In particular, we will use, for any $\Psi ,\Omega \in L^2({\mathbb {R}}^{2N}, {\mathbb {C}})$ the notation
  $$\begin{aligned} \langle \!\langle \Omega , \mathbb {1}^{\otimes (k-1)} \otimes F \otimes \mathbb {1}^{\otimes (N-k)}\Psi \rangle \!\rangle = \langle \!\langle \Omega , F(x_k) \Psi \rangle \!\rangle . \end{aligned}$$
  In analogy, for any two-particle multiplication operator $K{:}\,L^2({\mathbb {R}}^2, {\mathbb {C}}) ^{\otimes 2}\rightarrow L^2({\mathbb {R}}^2, {\mathbb {C}})^{\otimes 2}$, we denote the operator acting on any $\Psi \in L^2({\mathbb {R}}^{2N}, {\mathbb {C}})$ by multiplication in the variable $x_i$ and $x_j$ by $K(x_i,x_j)$. In particular, we denote
  $$\begin{aligned} \langle \!\langle \Omega , K(x_i,x_j) \Psi \rangle \!\rangle = \int _{{\mathbb {R}}^{2N}} K(x_i, x_j) \Omega ^*(x_1,\ldots ,x_N) \Psi (x_1,\ldots ,x_N) d^2x_1 \dots d^2x_N. \end{aligned}$$

Next, we prove some properties of the projectors $p_j$ and $q_j$, which are defined in Definition 3.1.

### Lemma 4.2

- For any weights $m,r{:}\,{\mathbb {N}}_0\rightarrow {\mathbb {R}}^+_0$ the commutation relations
  $$\begin{aligned} {\widehat{m}}{\widehat{r}}\,={\widehat{mr}}={\widehat{r}}\,{\widehat{m}},\;\;\;\;\;\;\;{\widehat{m}}p_j=p_j{\widehat{m}},\;\;\;\;\;\;\;{\widehat{m}}q_j=q_j{\widehat{m}},\;\;\;\;\;\;\;{\widehat{m}}P_{k}=P_{k}{\widehat{m}} \end{aligned}$$
  hold.
- Let $n{:}\,{\mathbb {N}}_0\rightarrow {\mathbb {R}}^+_0$ be given by $n(k)=\sqrt{k/N}$. Then, the square of ${\widehat{n}}$ equals the relative particle number operator of particles not in the state $\varphi$, i.e.
  14 $$\begin{aligned} \left( {\widehat{n}}\right) ^2=N^{-1}\sum _{j=1}^Nq_j. \end{aligned}$$
- For any weight $m{:}\,{\mathbb {N}}_0\rightarrow {\mathbb {R}}^+_0$ and any function $f \in L^\infty \left( {\mathbb {R}}^4,{\mathbb {C}}\right)$ and any $j,k=0,1,2$
  $$\begin{aligned} {\widehat{m}} Q_j f(x_1,x_2)Q_k= Q_j f(x_1,x_2){\widehat{m}}_{j-k}Q_k, \end{aligned}$$
  where $Q_0=p_1 p_2$, $Q_1\in \{p_1q_2,q_1p_2\}$ and $Q_2=q_1q_2$. Furthermore, for $j,k\in \{0,1\}$ and $g \in L^{\infty }({\mathbb {R}}^2,{\mathbb {C}})$ the relations
  $$\begin{aligned} {\widehat{m}} {\widetilde{Q}}_j g(x_1) {\widetilde{Q}}_k= {\widetilde{Q}}_j g(x_1) {\widehat{m}}_{j-k}{\widetilde{Q}}_k \quad \text {and} \quad {\widehat{m}} {\widetilde{Q}}_j \nabla _1 {\widetilde{Q}}_k= {\widetilde{Q}}_j \nabla _1{\widehat{m}}_{j-k}{\widetilde{Q}}_k \end{aligned}$$
  hold, where ${\widetilde{Q}}_0=p_1$ and ${\widetilde{Q}}_1=q_1$.
- For any weight $m:{\mathbb {N}}_0\rightarrow {\mathbb {R}}^+_0$ and any functions $f \in L^\infty \left( {\mathbb {R}}^4,{\mathbb {C}}\right)$, $g \in L^\infty \left( {\mathbb {R}}^2,{\mathbb {C}}\right)$ the commutation relations
  $$\begin{aligned}{}[f(x_1,x_2),{\widehat{m}}]&=\left[ f(x_1,x_2),p_1p_2({\widehat{m}}-{\widehat{m}}_2)+(p_1q_2+q_1p_2)({\widehat{m}}-{\widehat{m}}_1)\right] , \\ [g(x_1),{\widehat{m}}]&= q_1 g(x_1) ({\widehat{m}}-{\widehat{m}}_1) p_1 - p_1 ({\widehat{m}}-{\widehat{m}}_1) g(x_1) q_1 \end{aligned}$$
  hold.
- Let $$f\in L^1\left( {\mathbb {R}}^2,{\mathbb {C}}\right) $$, $$g\in L^2\left( {\mathbb {R}}^2,{\mathbb {C}}\right) $$. Then,
  15 $$\begin{aligned} \Vert p_j f(x_j-x_k)p_j\Vert _{\text {op}}&\le \Vert f\Vert _1\Vert \varphi \Vert _\infty ^2, \end{aligned}$$
  16 $$\begin{aligned} \Vert p_{j}g^{*}(x_j-x_k)\Vert _{\mathrm{{op}}}&= \Vert g(x_j-x_k)p_j\Vert _{\mathrm{{op}}}\le \Vert g\Vert \;\Vert \varphi \Vert _\infty , \end{aligned}$$
  17 $$\begin{aligned} \Vert |\varphi (x_j) \rangle \langle \nabla _j \varphi (x_j)| g^* (x_j-x_k)\Vert _{\text {op}}&= \Vert g(x_j-x_k)\nabla _j p_j\Vert _{\text {op}}\le \Vert g \Vert \Vert \nabla \varphi \Vert _{\infty }. \end{aligned}$$

### Proof

- follows immediately from Definition 3.1, using that $p_j$ and $q_j$ are orthogonal projectors.
- Note that $\cup _{k=0}^N{\mathcal {S}}_k=\{0,1\}^N$, so $1=\sum _{k=0}^N P_k$. Using also $(q_j)^2=q_j$ and $q_j p_j=0$ we get
  $$\begin{aligned} \sum _{j=1}^Nq_j=\sum _{j=1}^Nq_j\sum _{k=0}^N P_k= \sum _{k=0}^N\sum _{j=1}^Nq_j P_k=\sum _{k=0}^Nk P_k=N\widehat{n^2}=N{\widehat{n}}^2. \end{aligned}$$
- Using the definitions above we have
  $$\begin{aligned} {\widehat{m}} Q_j f(x_1,x_2)Q_k&=\sum _{l=0}^N m(l)P_l Q_jf(x_1,x_2)Q_k. \end{aligned}$$
  The number of projectors $q_j$ in $P_l Q_j$ in the coordinates $j=3,\ldots ,N$ is equal to $l-j$. The $p_j$ and $q_j$ with $j=3,\ldots ,N$ commute with $Q_jf(x_1,x_2)Q_k$. Thus $P_l Q_jf(x_1,x_2)Q_k= Q_jf(x_1,x_2)Q_kP_{l-j+k}$ and
  $$\begin{aligned} {\widehat{m}} Q_j f(x_1,x_2)Q_k&= \sum _{l=0}^N m(l) Q_jf(x_1,x_2)Q_kP_{l-j+k} \\&= \sum _{{\widetilde{l}}=k-j}^{N+k-j} Q_jf(x_1,x_2)m({\widetilde{l}}+j-k)P_{{\widetilde{l}}} Q_k =Q_j f(x_1,x_2){\widehat{m}}_{j-k}Q_k. \end{aligned}$$
  Similarly one gets the second and third formula.
- First note that
  18 $$\begin{aligned}&[f(x_1,x_2),{\widehat{m}}]-\left[ f(x_1,x_2),p_1p_2({\widehat{m}} -{\widehat{m}}_2)+p_1q_2({\widehat{m}}-{\widehat{m}}_1) +q_1p_2({\widehat{m}}-{\widehat{m}}_1)\right] \nonumber \\&\quad =[f(x_1,x_2),q_1q_2{\widehat{m}}]+\left[ f(x_1,x_2),p_1p_2{\widehat{m}}_2 +p_1q_2{\widehat{m}}_1 +q_1p_2{\widehat{m}}_1\right] . \end{aligned}$$
  We will show that the right hand side is zero. Multiplying the right hand side with $p_1p_2$ from the left and using (c) one gets
  $$\begin{aligned}&p_1p_2f(x_1,x_2)q_1q_2{\widehat{m}}+p_1p_2f(x_1,x_2)p_1p_2 {\widehat{m}}_2-p_1p_2{\widehat{m}}_2f(x_1,x_2) \\&\qquad +\,p_1p_2f(x_1,x_2)p_1q_2{\widehat{m}}_1+p_1p_2f(x_1,x_2)q_1p_2{\widehat{m}}_1 \\&\quad =p_1p_2{\widehat{m}}_2f(x_1,x_2)q_1q_2+p_1p_2{\widehat{m}}_2 f(x_1,x_2)p_1p_2-p_1p_2{\widehat{m}}_2f(x_1,x_2) \\&\qquad +\,p_1p_2{\widehat{m}}_2 f(x_1,x_2)p_1q_2+p_1p_2{\widehat{m}}_2 f(x_1,x_2)q_1p_2 \\&\quad =0. \end{aligned}$$
  Multiplying (18) with $p_1q_2$ from the left one gets
  $$\begin{aligned}&p_1q_2f(x_1,x_2)q_1q_2{\widehat{m}}+p_1q_2f(x_1,x_2)p_1p_2{\widehat{m}}_2+ p_1q_2f(x_1,x_2)p_1q_2{\widehat{m}}_1\\&\quad +\,p_1q_2f(x_1,x_2)q_1p_2{\widehat{m}}_1 -p_1q_2{\widehat{m}}_1f(x_1,x_2). \end{aligned}$$
  Using (c) the latter is zero. Also multiplying with $q_1p_2$ yields zero due to symmetry in interchanging $x_1$ with $x_2$. Multiplying (18) with $q_1q_2$ from the left one gets
  $$\begin{aligned}&q_1q_2f(x_1,x_2){\widehat{m}}q_1q_2-q_1q_2{\widehat{m}}f(x_1,x_2)+ q_1q_2f(x_1,x_2)p_1p_2{\widehat{m}}_2 \\&\quad +\,q_1q_2f(x_1,x_2)p_1q_2{\widehat{m}}_1+q_1q_2f(x_1,x_2)q_1p_2{\widehat{m}}_1 \end{aligned}$$
  which is again zero and so is (18). By means of the identity $1 = p_1 + q_1$ one has
  $$\begin{aligned}{}[g(x_1),{\widehat{m}}]&= p_1 \big ( g(x_1) {\widehat{m}} - {\widehat{m}} g(x_1) \big ) p_1 + q_1 \big ( g(x_1) {\widehat{m}} - {\widehat{m}} g(x_1) \big ) q_1 \\&\quad +\, q_1 \big ( g(x_1) {\widehat{m}} - {\widehat{m}} g(x_1) \big ) p_1 + p_1 \big ( g(x_1) {\widehat{m}} - {\widehat{m}} g(x_1) \big ) q_1. \end{aligned}$$
  The second relation from part (d) then follows from (a) and (c).
- To show (15), note that
  19 $$\begin{aligned} p_j f(x_j-x_k)p_j=p_j (f *|\varphi |^2)(x_k). \end{aligned}$$
  It follows that
  $$\begin{aligned} \Vert p_j f(x_j-x_k)p_j\Vert _{\text {op}}\le \Vert f\Vert _1\Vert \varphi \Vert _\infty ^2. \end{aligned}$$
  For (16) we write
  $$\begin{aligned} \Vert g(x_j-x_k)p_j\Vert _{\text {op}}^2&=\sup _{\Vert \Psi \Vert =1}\Vert g(x_j-x_k)p_j\Psi \Vert ^2 \\&=\sup _{\Vert \Psi \Vert =1}\langle \!\langle \Psi ,p_j |g(x_j-x_k)|^2 p_j\Psi \rangle \!\rangle \\&\le \Vert p_j |g(x_j-x_k)|^2p_j\Vert _{\text {op}}. \end{aligned}$$
  With (15) we get (16). For (17) we use
  $$\begin{aligned} \Vert g(x_j-x_k) \nabla _j p_j \Vert _{\text {op}}^ 2&= \sup _{\Vert \Psi \Vert =1} \langle \!\langle \Psi , p_j (|g|^ 2 * |\nabla \varphi |^2)(x_k) \Psi \rangle \!\rangle \le \Vert |g|^ 2 * |\nabla \varphi |^2\Vert _{\infty } \\&\le \Vert g\Vert ^2 \Vert \nabla \varphi \Vert _\infty ^2. \end{aligned}$$
  The Lemma then follows from the fact that, for bounded operators *A*, $\Vert A \Vert _{\text {op}}=\Vert A^* \Vert _{\text {op}}$ holds, where $A^*$ is the adjoint operator of *A*. $\square$

Within our estimates we will encounter wave functions where some of the symmetry is broken (at this point the reader should exemplarily think of the wave function $V_\beta (x_1-x_2)\Psi$ which is not symmetric under exchange of the variables $x_1$ and $x_3$). This leads to the following definition

### Definition 4.3

For any finite set ${\mathcal {M}}\subset \{1,2,\ldots ,N\}$, define the space ${\mathcal {H}}_{{\mathcal {M}}}\subset L^2({\mathbb {R}}^{2N},{\mathbb {C}})$ as the set of functions which are symmetric in all variables in ${\mathcal {M}}$

$$\begin{aligned}&\Psi \in {\mathcal {H}}_{{\mathcal {M}}}\Leftrightarrow \Psi (x_1,\ldots ,x_j,\ldots ,x_k,\ldots ,x_N)=\Psi (x_1,\ldots ,x_k,\ldots ,x_j,\ldots ,x_N)\\&\quad \text { for all } j,k\in {\mathcal {M}}. \end{aligned}$$

Based on the combinatorics of the $p_j$ and $q_j$, we obtain the following

### Lemma 4.4

For any $f{:}\,{\mathbb {N}}_0\rightarrow {\mathbb {R}}^+_0$ and any finite set ${\mathcal {M}}_a\subset \{1,2,\ldots ,N\}$ with $1\in {\mathcal {M}}_a$ and any finite set ${\mathcal {M}}_b\subset \{1,2,\ldots ,N\}$ with $1,2\in {\mathcal {M}}_b$

20 $$\begin{aligned} \left\| {\widehat{f}} q_1\Psi \right\| ^2&\le \frac{N}{|{\mathcal {M}}_a|} \Vert {\widehat{f}}{\widehat{n}}\Psi \Vert ^2\;\;\;\;\text { for any }\Psi \in {\mathcal {H}}_{{\mathcal {M}}_a}, \end{aligned}$$

21 $$\begin{aligned} \left\| {\widehat{f}} q_1 q_2\Psi \right\| ^2&\le \frac{N^2}{|{\mathcal {M}}_b|(|{\mathcal {M}}_b|-1)} \Vert {\widehat{f}}({\widehat{n}})^2\Psi \Vert ^2\;\;\;\;\;\text { for any }\Psi \in {\mathcal {H}}_{{\mathcal {M}}_b}. \end{aligned}$$

### Proof

Let $\Psi \in {\mathcal {H}}_{{\mathcal {M}}_a}$ for some finite set $1\in {\mathcal {M}}_a\subset \{1,2,\ldots ,N\}$. By Lemma 4.2 (b), (20) can be estimated as

$$\begin{aligned}\Vert {\widehat{f}}{\widehat{n}}\Psi \Vert ^2&=\langle \!\langle \Psi ,({\widehat{f}})^{2}({\widehat{n}})^2\Psi \rangle \!\rangle =N^{-1}\sum _{k=1}^N\langle \!\langle \Psi ,({\widehat{f}})^{2}q_k\Psi \rangle \!\rangle \\&\ge N^{-1}\sum _{k\in {\mathcal {M}}_a}\langle \!\langle \Psi ,({\widehat{f}})^{2}q_k\Psi \rangle \!\rangle =\frac{|{\mathcal {M}}_a|}{N}\langle \!\langle \Psi ,({\widehat{f}})^{2}q_1\Psi \rangle \!\rangle \\&= \frac{|{\mathcal {M}}_a|}{N}\Vert {\widehat{f}} q_1\Psi \Vert ^2. \end{aligned}$$

Similarly, we obtain for $\Psi \in {\mathcal {H}}_{{\mathcal {M}}_b}$

$$\begin{aligned} \Vert {\widehat{f}} ({\widehat{n}})^2\Psi \Vert ^2&=\langle \!\langle \Psi ,({\widehat{f}} )^2({\widehat{n}})^4\Psi \rangle \!\rangle \ge N^{-2}\sum _{j,k\in {\mathcal {M}}_b}\langle \!\langle \Psi ,({\widehat{f}} )^2q_j q_k\Psi \rangle \!\rangle \\&=\frac{|{\mathcal {M}}_b|(|{\mathcal {M}}_b|-1)}{N^2}\langle \!\langle \Psi ,({\widehat{f}} )^2q_1 q_2\Psi \rangle \!\rangle +\frac{|{\mathcal {M}}_b|}{N^2}\langle \!\langle \Psi ,({\widehat{f}} )^2q_1\Psi \rangle \!\rangle \\&\ge \frac{|{\mathcal {M}}_b|(|{\mathcal {M}}_b|-1)}{N^2} \Vert {\widehat{f}} q_1q_2\Psi \Vert ^2 \end{aligned}$$

which concludes the Lemma. $\quad \square$

### Corollary 4.5

Let $\Psi \in L^2_{s}({\mathbb {R}}^{2N}, {\mathbb {C}})$. For any weight $m{:}\,{\mathbb {N}}_0\rightarrow {\mathbb {R}}^+_0$

22 $$\begin{aligned} \Vert \nabla _2 {\widehat{m}}q_2\Psi \Vert&\le 2\Vert {\widehat{m}}\Vert _{\text {op}}\Vert \nabla _2q_2\Psi \Vert , \end{aligned}$$

23 $$\begin{aligned} \Vert \nabla _2 {\widehat{m}}q_1q_2\Psi \Vert&\le C\Vert {\widehat{m}}{\widehat{n}}\Vert _{\text {op}}\Vert \nabla _2q_2\Psi \Vert . \end{aligned}$$

### Proof

Using $p_2+q_2=1$ and triangle inequality,

24 $$\begin{aligned} \Vert \nabla _2 {\widehat{m}}q_2\Psi \Vert&\le \Vert p_2\nabla _2 {\widehat{m}}q_2\Psi \Vert +\Vert q_2\nabla _2 {\widehat{m}}q_2\Psi \Vert , \end{aligned}$$

25 $$\begin{aligned} \Vert \nabla _2 {\widehat{m}}q_1q_2\Psi \Vert&\le \Vert p_2\nabla _2 {\widehat{m}}q_1q_2\Psi \Vert +\Vert q_2\nabla _2 {\widehat{m}}q_1q_2\Psi \Vert . \end{aligned}$$

With Lemma 4.2 (c) we get

$$\begin{aligned} (24)&=\Vert {\widehat{m}}_{1}p_2\nabla _2q_2\Psi \Vert +\Vert {\widehat{m}}q_2\nabla _2q_2\Psi \Vert \le (\Vert {\widehat{m}}_{1}\Vert _{\text {op}}+\Vert {\widehat{m}}\Vert _{\text {op}})\Vert \nabla _2q_2\Psi \Vert . \end{aligned}$$

Note that the wave function $p_2\nabla _2q_2\Psi$ is symmetric under the exchange of any two variables but $x_2$. Thus we can use Lemma 4.4 to get

$$\begin{aligned} (25)&=\Vert q_1{\widehat{m}}_{1}p_2\nabla _2q_2\Psi \Vert +\Vert q_1{\widehat{m}}q_2\nabla _2q_2\Psi \Vert \\&\le \frac{N}{N-1}(\Vert {\widehat{m}}_{1}{\widehat{n}}\Vert _{\text {op}}+\Vert {\widehat{m}}{\widehat{n}}\Vert _{\text {op}})\Vert \nabla _2q_2\Psi \Vert . \end{aligned}$$

Since $\sqrt{k}\le \sqrt{k+1}$ for $k\ge 0$ it follows that the latter is bounded by

$$\begin{aligned} C(\Vert {\widehat{m}}_{1}{\widehat{n}}_{1}\Vert _{\text {op}} +\Vert {\widehat{m}}{\widehat{n}}\Vert _{\text {op}})\Vert \nabla _2q_2\Psi \Vert . \end{aligned}$$

Using that $\Vert {\widehat{r}}\Vert _{\text {op}}=\sup _{0\le k \le N}\{r(k)\}=\Vert {\widehat{r}}_d\Vert _{\text {op}}$ for any $d\in {\mathbb {N}}$ and any weight *r*, the Corollary follows. $\quad \square$

### Lemma 4.6

Let $\Omega ,\chi \in {\mathcal {H}}_{{\mathcal {M}}}$ for some ${\mathcal {M}}$, let $1\notin {\mathcal {M}}$ and $2,3\in {\mathcal {M}}$. Let $O_{j,k}$ be an operator acting on the $j^{th}$ and $k^{th}$ coordinate. Then

$$\begin{aligned} |\langle \!\langle \Omega ,O_{1,2}\chi \rangle \!\rangle |&\le \Vert \Omega \Vert ^2+\left| \langle \!\langle O_{1,2}\chi ,O_{1,3}\chi \rangle \!\rangle \right| +(|{\mathcal {M}}|)^{-1}\Vert O_{1,2}\chi \Vert ^2. \end{aligned}$$

### Proof

Using symmetry and Cauchy Schwarz

$$\begin{aligned} |\langle \!\langle \Omega ,O_{1,2}\chi \rangle \!\rangle |&=|{\mathcal {M}}|^{-1}|\langle \!\langle \Omega ,\sum _{j\in {\mathcal {M}}} O_{1,j}\chi \rangle \!\rangle |\le |{\mathcal {M}}|^{-1}\Vert \Omega \Vert \;\Vert \sum _{j\in {\mathcal {M}}} O_{1,j}\chi \Vert . \end{aligned}$$

For the second factor we can write

$$\begin{aligned} \Vert \sum _{j\in {\mathcal {M}}} O_{1,j}\chi \Vert ^2&=\langle \!\langle \sum _{j\in {\mathcal {M}}} O_{1,j}\chi ,\sum _{k\in {\mathcal {M}}} O_{1,k}\chi \rangle \!\rangle \\&\le \sum _{j\in {\mathcal {M}}}|\langle \!\langle O_{1,j}\chi ,O_{1,j}\chi \rangle \!\rangle |+|\sum _{j\ne k\in {\mathcal {M}}}\langle \!\langle O_{1,j}\chi ,O_{1,k}\chi \rangle \!\rangle | \\&\le |{\mathcal {M}}||\langle \!\langle O_{1,2}\chi ,O_{1,2}\chi \rangle \!\rangle |+|{\mathcal {M}}|(|{\mathcal {M}}|-1)|\langle \!\langle O_{1,2}\chi ,O_{1,3}\chi \rangle \!\rangle |. \end{aligned}$$

Since $ab\le 1/2a^2+1/2b^2$ and $(a+b)^2\le 2a^2+2b^2$ holds for any real numbers *a* and *b*, the Lemma follows. $\quad \square$

In our estimates, we need the regularity conditions

$$\begin{aligned} \Vert \nabla \varphi _t \Vert _\infty< \infty , \qquad \Vert \varphi _t \Vert _\infty< \infty , \qquad \Vert \nabla \varphi _t \Vert< \infty , \qquad \Vert \Delta \varphi _t \Vert < \infty . \end{aligned}$$

That is, we need $\varphi _t \in H^2({\mathbb {R}}^2,{\mathbb {C}}) \cap W^{1,\infty }({\mathbb {R}}^2,{\mathbb {C}})$. Then, $\Vert \Delta |\varphi _t| ^2 \Vert , \Vert \Delta |\varphi _t| ^2 \Vert _1$ and $\Vert \varphi _t^ 2 \Vert$, which also appear in our estimates, can be bounded by

$$\begin{aligned} \Delta |\varphi _t|^ 2&= \varphi ^*_t \Delta \varphi _t + \varphi _t \Delta \varphi ^*_t + 2 (\nabla \varphi ^*_t) \cdot (\nabla \varphi _t) \\ \Vert \Delta |\varphi _t| ^2 \Vert&\le 2 \Vert \Delta \varphi _t \Vert \Vert \varphi _t \Vert _\infty + 2 \Vert \nabla \varphi _t \Vert \Vert \nabla \varphi _t \Vert _\infty \\ \Vert \Delta |\varphi _t| ^2 \Vert _1&\le 4 \Vert \Delta \varphi _t \Vert \\ \Vert \varphi _t^ 2 \Vert&\le \Vert \varphi _t \Vert _\infty \Vert \varphi _t \Vert . \end{aligned}$$

Recall the Sobolev embedding Theorem, which implies in particular $H^k({\mathbb {R}}^2,{\mathbb {C}})= W^{k,2}({\mathbb {R}}^ 2,{\mathbb {C}}) \subset C^{k-2}({\mathbb {R}}^2,{\mathbb {C}})$. If $\varphi \in C^1({\mathbb {R}}^2,{\mathbb {C}}) \cap H^1({\mathbb {R}}^2,{\mathbb {C}})$, then $\varphi \in W^{1,\infty }({\mathbb {R}}^2,{\mathbb {C}})$ follows since both $\varphi$ and $\nabla \varphi$ have to decay at infinity. Thus, $\varphi _t \in H^3({\mathbb {R}}^2,{\mathbb {C}})$ implies $\varphi _t \in H^2({\mathbb {R}}^2,{\mathbb {C}}) \cap W^{1,\infty }({\mathbb {R}}^2, {\mathbb {C}})$, which suffices for our estimates. Since $\varphi _t$ obeys a defocusing nonlinear Schrödinger equation, we expect the regularity of the solution $\varphi _t$ to follow from the regularity of the initial datum $\varphi _0$. For a certain class of external potentials $A_t$ this has been proven in [13]:

### Lemma 4.7

Let $\varphi _0 \in \Sigma ^k({\mathbb {R}}^2,{\mathbb {C}}) = \lbrace f \in L^2 ({\mathbb {R}}^2, {\mathbb {C}})| \sum _{ \alpha + \beta \le k} \Vert x^\alpha \partial _x^\beta f \Vert < \infty \rbrace$, for $k\ge 2$. Let, for $b>0$, $\varphi _t$ be the unique solution to

$$\begin{aligned} i \partial _t \varphi _t = (-\Delta +A_t+ b |\varphi _t|^2) \varphi _t. \end{aligned}$$

Let $A_{\cdot } \in L^\infty _{\text {loc}}({\mathbb {R}}_t \times {\mathbb {R}}^2_x, {\mathbb {C}})$ real valued and smooth with respect to the space variable: for (almost) all $t \in {\mathbb {R}}$, the map $x \mapsto A_t(x)$ is $C^\infty$. Moreover, $A_t$ is at most quadratic in space, uniformly w.r.t. time *t*:

$$\begin{aligned} \forall \alpha \in {\mathbb {N}}^2, | \alpha | \ge 2, \quad \partial _x^\alpha A_{\cdot } \in L^\infty ({\mathbb {R}}_t \times {\mathbb {R}}^d_x, {\mathbb {C}}). \end{aligned}$$

In addition, $t \mapsto \sup _{|x| \le 1} |A_t(x)|$ belongs to $L^{\infty }({\mathbb {R}}, {\mathbb {C}})$. Then

- $$ \varphi _t \in \Sigma ^k({\mathbb {R}}^2,{\mathbb {C}})$$, which implies $$ \varphi _t \in H^k({\mathbb {R}}^2,{\mathbb {C}})$$.
- $$\Vert \varphi _t\Vert =\Vert \varphi _0 \Vert $$.
- Let $\varphi _0 \in \Sigma ^3({\mathbb {R}}^2,{\mathbb {C}})$. Assume in addition that $A_{\cdot } \in C^1({\mathbb {R}},L^{\infty }({\mathbb {R}}^2,{\mathbb {R}}))$. Then, for any fixed $t \ge 0$, ${\mathcal {K}}(\varphi _t, A_t) < \infty$ follows.

### Proof

Part (a) is Corollary 1.4. in [13]. We like to remark that $\Vert \varphi _t \Vert _{H^k} \le C$ holds, if $A_t=0$, see Section 1.2. in [13]. The conditions on $A_t$ are for example satisfied if $A_t \in C^\infty _c ({\mathbb {R}}^2, {\mathbb {R}})$ for all $t \in {\mathbb {R}}$, $A_t(x)=0$, for all $|t| \ge T$. Part (b) can be verified directly, using the existence of global in time solutions. Part (c) follows from (a) and the embedding $H^3({\mathbb {R}}^2,{\mathbb {C}}) \subset H^2({\mathbb {R}}^2,{\mathbb {C}}) \cap W^{1,\infty }({\mathbb {R}}^2, {\mathbb {C}})$. $\quad \square$

## Microscopic Structure in 2 Dimensions

### The scattering state

In this section we analyze the microscopic structure which is induced by $V_N$. In particular, we explain why the dynamical properties of the system are determined by the low energy scattering regime.

#### Definition 5.1

Let $V_N \in {\mathcal {V}}_N$. For any $R \ge \text {diam}(\text {supp} (V_N))$, we define the zero energy scattering state $j_{N,R}\in C^1({\mathbb {R}}^2,{\mathbb {R}})$ by

26 $$\begin{aligned} {\left\{ \begin{array}{ll} \left( - \Delta _x + \frac{1}{2}e^{2N} V(e^N x) \right) j_{N,R}(x)=0, \\ j_{N,R}(x)=1 \; \text {for } |x| = R. \end{array}\right. } \end{aligned}$$

Next, we want to recall some important properties of the scattering state $j_{N,R}$, see also Appendix C of [38].

#### Lemma 5.2

Let $V_N \in {\mathcal {V}}_N$. Define $I_{R} = \int _{{\mathbb {R}}^2} d^2x V_N(x) j_{N,R}(x)$. For the scattering state defined previously the following relations hold:

- There exists a nonnegative number *a*, called scattering length of the potential *V*, such that
  $$\begin{aligned} I_{R}= \frac{4 \pi }{\ln \left( \frac{e^N R}{a}\right) } \end{aligned}$$
  (in the case $$a=0$$ we have $$I_R=0)$$. The scattering length *a* does not depend on *R* and fulfills $$a \le \text {diam}(\text {supp}(V))$$. Furthermore, $$I_R \ge 0$$ holds.
- $j_{N,R}$ is a nonnegative function which is spherically symmetric in |*x*|. For $|x| \ge \text {diam}(\text {supp}(V_N))$, $j_{N,R}$ is given by
  $$\begin{aligned} j_{N,R} (x) = 1 + \frac{1}{\ln \left( \frac{e^N R}{a}\right) } \ln \left( \frac{|x|}{R} \right) . \end{aligned}$$

#### Proof

- (a)+(b) Rescaling $x \rightarrow e^{N} x =y$, we obtain, setting ${\tilde{R}}=e^{N} R$ and $s_{{\tilde{R}}}(y)= j_{0, e^{N}R}(y)$, the unscaled scattering equation
  27 $$\begin{aligned} {\left\{ \begin{array}{ll} \left( - \Delta _y + \frac{1}{2}V(y) \right) s_{{\tilde{R}}}(y)=0, \\ s_{{\tilde{R}}}(y)=1 \; \text {for } |y| ={\tilde{R}}. \end{array}\right. } \; \end{aligned}$$
  Since we assume *V* to be nonnegative, one can define the scattering state $s_{{\tilde{R}}}$ by a variational principle. Theorem C.1 in [38] then implies that $s_{{\tilde{R}}}$ is a nonnegative, spherically symmetric function in |*y*|. It is then easy to verify that for $\text {diam (supp} (V)) \le |y|$ there exists a number $A\in {\mathbb {R}}$ such that
  28 $$\begin{aligned} s_{{\tilde{R}}}(y) = 1 + \frac{A}{4 \pi } \ln \left( \frac{|y|}{{\tilde{R}}} \right) . \end{aligned}$$
  Next, we show that $A= \int _{{\mathbb {R}}^2}d^2y V(y) s_{{\tilde{R}}} (y)$. This can be seen by noting that, for $r > \text {diam (supp} (V))$,
  $$\begin{aligned} \int _{{\mathbb {R}}^2}d^2y V(y) s_{{\tilde{R}}}(y)&= 2 \int _{B_r(0)}d^2y \Delta s_{{\tilde{R}}}(y) = 2 \int _{\partial B_r(0)} \nabla s_{{\tilde{R}}}(y) \cdot ds \\&= \frac{A}{2 \pi } \int _{\partial B_r(0)} \nabla \ln (|y|) \cdot ds = \frac{A}{2 \pi } \int _{0}^{2 \pi } \frac{1}{r} r d\varphi \\&= A. \end{aligned}$$
  By Theorem C.1 in [38], there exists a number $a \ge 0$, not depending on ${\tilde{R}}$, such that for all $|y| \ge \text {diam (supp} (V))$
  $$\begin{aligned} s_{{\tilde{R}}}(y)= \frac{\ln (|y|/a) }{\ln ({\tilde{R}}/a)}. \end{aligned}$$
  Comparing this with (28), we obtain
  $$\begin{aligned} \int _{{\mathbb {R}}^2} V(y) s_{{\tilde{R}}}(y) dy^2= \frac{ 4 \pi }{\ln \left( \frac{{\tilde{R}}}{a}\right) }. \end{aligned}$$
  Since $s_{{\tilde{R}}}$ is nonnegative, it furthermore follows that $a \le \text {diam (supp} (V))$. This directly implies $A \ge 0$. By scaling, we obtain
  $$\begin{aligned} I_R= \int _{{\mathbb {R}}^2} V_N(y) j_{N,R}(y) dy^2 = \int _{{\mathbb {R}}^2} V(y) s_{{\tilde{R}}}(y) dy^2 = \frac{ 4 \pi }{ \ln \left( \frac{e^N R}{a}\right) }. \end{aligned}$$

$$\quad \square $$

Assuming that the energy per particle ${\mathcal {E}}_{V_N}(\Psi )$ is of order one, the wave function $\Psi$ will have a microscopic structure near the interactions $V_N$, given by $j_{N,R}$. The interaction among two particles is then determined by $\frac{4 \pi }{N+ \ln \left( \frac{R}{a}\right) } \approx \frac{4 \pi }{N}$. Keeping in mind that each particle interacts with all other $N-1$ particles, we obtain the effective Gross–Pitaevskii equation, for $\varphi _t \in H^2( {\mathbb {R}}^2, {\mathbb {C}})$

$$\begin{aligned} i \partial _t \varphi _t(x) = (- \Delta +A_t+ 4 \pi | \varphi _t(x)|^2) \varphi _t(x). \end{aligned}$$

Thus, choosing $V_N(x)= e^{2N} V(e^Nx)$ leads in our setting to an effective one-particle equation which is determined by the low energy scattering behavior of the particles. We remark that, for any $s>0$, the potential $e^{2Ns} V(e^{Ns}x)$ yields to the coupling $4 \pi / s$.

### Properties of the scattering state

Note that the potential $V_N$ is strongly peaked within an exponentially small region. In order to control the short scale structure of $\Psi _t$, we define a potential $M_{\mu }$ with softer scaling behaviour in such a way that the potential $V_N -M_{\mu }$ has scattering length zero. This allows us to “replace” $V_N$ by $M_{\mu }$, which has better scaling behavior and is easier to control. In particular, $\Vert M_{\mu }\Vert \le CN ^{-1+\mu }$ can be controlled for $\mu$ sufficiently small.

#### Definition 5.3

Let $V_N \in {\mathcal {V}}_N$. For any $\mu >0$ and any $R_\mu \ge N^{- \mu }$ we define the potential $M_{\mu }$ via

29 $$\begin{aligned} M_{\mu }(x) = {\left\{ \begin{array}{ll} 4 \pi N^{-1+2 \mu } &{}\quad \text {if } N^{- \mu } < |x| \le R_\mu , \\ 0 &{}\quad \text {else }. \end{array}\right. } \end{aligned}$$

Furthermore, we define the zero energy scattering state $f_\mu \in C^1({\mathbb {R}}^2,{\mathbb {R}})$ of the potential $\frac{1}{2} (V_N-M_{\mu })$, that is

30 $$\begin{aligned} {\left\{ \begin{array}{ll} \left( - \Delta _x + \frac{1}{2} \left( V_N(x)-M_{\mu }(x) \right) \right) f_{\mu }(x)=0, \\ f_{\mu }(x)=1 \; \text {for } |x| = R_\mu . \end{array}\right. } \end{aligned}$$

Note that $M_{\mu }$ and $f_\mu$ depend on $R_\mu$.

#### Remark 5.4

In the following, we choose $R_\mu$ to be the smallest value such that the scattering length of the potential $(V_N-M_{\mu })$ is zero which is equivalent to the condition $\int _{{\mathbb {R}}} d^2x (V_N(x)-M_{\mu }(x)) f_\mu (x) =0$. The existence of such $R_\mu <\infty$ will be proven in Lemma 5.5.

Note, that choosing $R_\mu$ to be the minimal value such that $(V_N-M_{\mu })$ has scattering length zero excludes the possibility for bound states for the potential. This will be shown in Lemma 7.10 (a). Heuristically speaking, the absence of bound states can be seen in the following way: The attractive part of the potential, i.e. $-M_{\mu }$, is chosen to be as small as possible, i.e. just to compensates the repulsive part. Then, there is not enough attractiveness left to form a bound state.

#### Lemma 5.5

For the scattering state $f_\mu$, defined by (30), the following relations hold : 

- There exists a minimal value $R_\mu <\infty$ such that $\int _{{\mathbb {R}}^2} d^2x (V_N(x)-M_{\mu }(x)) f_\mu (x) =0$.

For the rest of the paper we assume that $R_\mu$ is the minimum we get in (*a*).

(b) There exists $K_{\mu } \in {\mathbb {R}}, \; K_{\mu }> 0$ such that $K_{\mu } f_{\mu }(x) = j_{N,R_\mu }(x) \; \forall |x| \le N^{-\mu }$.
(c) For *N* sufficiently large the supports of $V_N$ and $M_{\mu }$ do not overlap.
(d) $f_{\mu }$ is a positive, monotone nondecreasing function in |*x*|.
(e) 31 $$\begin{aligned} f_{\mu }(x)=1 \quad \text {for } |x| \ge R_\mu . \end{aligned}$$
(f) 32 $$\begin{aligned} 1 \ge K_\mu \ge 1+ \frac{1}{N+ \ln \left( \frac{R_\mu }{a}\right) } \ln \left( \frac{N^{-\mu }}{R_\mu } \right) . \end{aligned}$$
(g) $$ R_\mu \le CN ^{-\mu }$$.

For any fixed $0<\mu$, *N* sufficiently large such that $V_N$ and $M_{\mu }$ do not overlap, we obtain

(h) $$\begin{aligned}&| N \Vert V_{N}f_{\mu } \Vert _1 - 4 \pi | = | N \Vert M_{\mu }f_{\mu } \Vert _1 - 4 \pi | \le C \frac{\ln (N)}{N}. \end{aligned}$$
(i) Define
  $$\begin{aligned} g_\mu (x) = 1 - f_\mu (x). \end{aligned}$$
  Then,
  $$\begin{aligned} \Vert g_{\mu }\Vert _1&\le CN ^{-1-2\mu }\ln (N) ,\qquad \Vert g_{\mu }\Vert \le CN ^{-1-\mu }\ln (N),\qquad \Vert g_{\mu }\Vert _\infty \le 1. \end{aligned}$$
(j) $$\begin{aligned} | N \Vert M_{\mu }\Vert _1 - 4 \pi | \le C \frac{\ln (N)}{N}. \end{aligned}$$
(k) $$\begin{aligned} M_{\mu } \in {\mathcal {W}}_\mu , M_{\mu } f_\mu \in {\mathcal {W}}_\mu . \end{aligned}$$

#### Proof

- In the following, we will sometimes denote, with a slight abuse of notation, $f_{\mu }(x)=f_{\mu }(r)$ and $j_{N,R}(x)=j_{N,R}(r)$ for $r=|x|$ (for this, recall that $f_{\mu }$ and $j_{N,R}$ are radially symmetric). We further denote by $f'_{\mu }(r)$ the derivative of $f_{\mu }$ with respect to *r*. We first show by contradiction that there exists a $x_0 \in {\mathbb {R}}^2,\; |x_0|\le N^{-\mu }$, such that $f_\mu (x_0) \ne 0$. For this, assume that $f_{\mu } (x) =0$ for all $|x| \le N^{-\mu }$. Since $f_{\mu }$ is continuous, there exists a maximal value $r_0 \ge N^{-\mu }$ such that the scattering equation (30) is equivalent to
  33 $$\begin{aligned} {\left\{ \begin{array}{ll} \left( - \Delta _x - \frac{1}{2} M_{\mu }(x) \right) f_{\mu }(x)=0, \\ f_{\mu }(x)=1 \; \text {for } |x| = R_{\mu }, \\ f_{\mu }(x)=0 \; \text {for } |x| \le r_0. \end{array}\right. } \end{aligned}$$
  Using (30) and Gauss’-theorem, we further obtain
  34 $$\begin{aligned} f'_{\mu }(r) = \frac{1}{ 4 \pi r } \int _{B_r(0)} d^2 x (V_N(x)-M_{\mu }(x)) f_\mu (x). \end{aligned}$$
  (33) and (34) then imply for $r > r_0$
  $$\begin{aligned} \left| f'_{\mu }(r) \right|&= \frac{1}{ 4 \pi r } \left| \int _{B_r(0)} d^2 x M_{\mu }(x) f_{\mu }(x) \right| = \frac{2 \pi N^{-1+2 \mu } }{ r } \left| \int _{r_0}^r dr' r'f_{\mu }(r') \right| \\&\le \frac{2 \pi N^{-1+2 \mu } }{ r} \left| \int _{r_0}^r dr' r' (r'-r_0) \sup _{r_0 \le s \le r} |f'_{\mu }(s)| \right| . \end{aligned}$$
  Taking the supreme over the interval $[r_0,r]$, the inequality above then implies that there exists a constant $C(r,r_0) \ne 0$, $\lim \nolimits _{r \rightarrow r_0} C(r,r_0)=0$ such that
  $$\begin{aligned} \sup \limits _{r_0 \le s \le r} |f'_{\mu }(s)| \le C(r,r_0) N^{-1+ 2 \mu } \sup \limits _{r_0 \le s \le r} |f'_{\mu }(s)|. \end{aligned}$$
  Thus, for *r* close enough to $r_0$, the inequality above can only hold if $f'_{\mu }(s)=0$ for $s \in [r_0,r]$, yielding a contradiction to the choice of $r_0$. Consequently, there exists a $x_0 \in {\mathbb {R}}^2, |x_0| \le N^{-\mu }$, such that $f_{\mu }(x_0) \ne 0$. We can thus define
  $$\begin{aligned} h(x) = f_{\mu }(x)\frac{j_{N,R}(x_0)}{f_{\mu }(x_0)} \end{aligned}$$
  on the compact set $\overline{B_{x_0}(0)}$. One easily sees that $h(x)= j_{N,R}(x)$ on $\partial \overline{B_{x_0}(0)}$ and satisfies the zero energy scattering equation (26) for $x \in \overline{B_{N^{-\mu }}(0)}$. Note that the scattering equations (26) and (30) have a unique solution on any compact set. It then follows that $h(x) = j_{N, R}(x) \; \forall x \in \overline{B_{N^{-\mu }}(0)}$. Since $j_{N, R} (N^{-\mu }) \ne 0$, we then obtain $f_{\mu }(N^{-\mu }) \ne 0$. Applying Theorem C.1 in [38] once more, it then follows that either $f_\mu$ or $-f_\mu$ is a nonnegative, monotone nondecrasing function in |*x*| for all $|x| \le N^{-\mu }$. Recall that $M_{\mu }$ and hence $f_{\mu }(x)$ depend on $R_\mu \in [N^{-\mu }, \infty [$. For conceptual clarity, we denote $M_{\mu }^{(R_{\mu })}(x) =M_{\mu }(x)$ and $f_{\mu }^{(R_{\mu })}(x)= f_{\mu }(x)$ for the rest of the proof of part (a). For $\mu$ fixed, consider the function
  $$\begin{aligned}&s{:}\,[N^{-\mu },\infty [ \rightarrow {\mathbb {R}} \\&R_{\mu } \mapsto \int _{B_{R_{\mu }}(0)} d^2 x (V_N(x)-M^{(R_{\mu })}_{\mu }(x)) f^{(R_{\mu })}_{\mu } (x). \end{aligned}$$
  We show by contradiction that the function *s* has at least one zero. Assume $s \ne 0$ were to hold. We can assume w.l.o.g. $s >0$. It then follows from Gauss’-theorem that $f'^{(R_{\mu })}_{\mu }(R_{\mu })> 0$ for all $R_\mu \ge N^{-\mu }$. By uniqueness of the solution of the scattering equation (30), for ${\tilde{R}}_{\mu }<R_{\mu }$ there exists a constant $K_{{\tilde{R}}_{\mu },R_{\mu }} \ne 0$, such that for all $|x|\le {\tilde{R}}_{\mu }$ we have $f^{({\tilde{R}}_{\mu })}_{\mu }(x)= K_{{\tilde{R}}_{\mu },R_{{\mu }}}f^{(R_{\mu })}_{\mu }(x)$. Since $f^{(R_{\mu })}_{\mu }$ and *s* are continuous, we can further conclude $K_{{\tilde{R}}_{\mu },R_{{\mu }}}> 0$. From $s \ne 0$, it then follows that, for all $r \in [N^{-\mu },\infty [$ and for all $R_\mu \in [N^{-\mu },\infty [$, $f'^{(R_{\mu })}_{\mu }(r) \ne 0$. Thus, for all $r \in [N^{-\mu },\infty [$ and for all $R_\mu \in [N^{-\mu _1},\infty [$, the function $f^{(R_{\mu })}_{\mu }(r)$ doesn’t change sign. From Lemma 5.2, the assumption $s(N^{-\mu })>0$ and $K_{{\tilde{R}}_{\mu },R_{{\mu }}} > 0$, we obtain, for all $r \in [0, N^{-\mu } ]$ and for all $R_{\mu } \in [N^{-\mu },\infty [$, that $f^{(R_{\mu })}_{\mu } (r) \ge 0$ holds. This, however, implies $\lim \nolimits _{R_{\mu } \rightarrow \infty } s(R_{\mu })= - \infty$ yielding to a contradiction. By continuity of *s*, there exists thus a minimal value $R_{\mu } \ge N^{-\mu }$ such that $s(R_{\mu })=0$.

#### Remark 5.6

As mentioned, we will from now on fix $R_{\mu } \in [N^{- \mu }, \infty [$ as the minimal value such that $s(R_{\mu })=0$. Furthermore, we may assume $a>0$ and $R_{\mu } >N^{-\mu }$ in the following. For $a=0$, we can choose $R_{\mu }=N^{-\mu }$, such that $f_{\mu }(x)=j_{N,R}(x)$. It is then easy to verify that the Lemma stated is valid.

(b) From (a), we can conclude that
  35 $$\begin{aligned} K_{\mu } = \frac{j_{N,R_\mu }(N^{-\mu })}{f_\mu (N^{-\mu })}. \end{aligned}$$
  Next, we show that the constant $K_\mu$ is positive. Since $j_{N,R_\mu }(N^{-\mu })$ is positive, it follows from Eq. (35) that $K_\mu$ and $f_\mu (N^ {-\mu })$ have equal sign. By (a), the sign of $f_\mu$ is constant for $|x|\le R_\mu$. Since $j_{N,R_\mu }$ and $V_N$ are nonnegative functions, we obtain by Gauss-theorem and the scattering equation (30)
  36 $$\begin{aligned} \text {sgn} \left( \frac{\partial f_\mu }{\partial r}|_{r= N^{- \mu }}\right) = \text {sgn}(K_\mu ). \end{aligned}$$
  Recall that $R_\mu$ is the smallest value such that $\frac{\partial f_\mu }{\partial r} \big |_{r=R_\mu } = 0$. If it were now that $K_\mu$ is negative, we could conclude from (35) and (36) that $\frac{\partial f_\mu }{\partial r}|_{r= N^{- \mu }} <0$ and $f_\mu ( N^{- \mu }) <0$. Since $R_\mu$ is by definition the smallest value where $\frac{\partial f_\mu }{\partial r} = 0$, we were able to conclude from the continuity of the derivative that $\frac{\partial f_\mu }{\partial r} <0$ for all $r < R_\mu$ and hence $f(R_\mu ) <0$. However, this were in contradiction to the boundary condition of the zero energy scattering state [see (30)] and thus $K_\mu > 0$ follows.
(c) This directly follows from $e^{-N} < CN ^{- \mu }$ for *N* sufficiently large.
(d) From the proof of property (b), we see that $f_\mu$ and its derivative is positive at $N^{- \mu }$. From (34), we obtain $f'_\mu (r) = 0$ for all $r > R_\mu$. Further (34) gives that $R_\mu$ is the smallest value such that $f'_\mu (R_\mu ) = 0$. This and continuity imply that $f'_{\mu }(r) >0$ for all $r< R_\mu$. Since $f_\mu$ is continuous, positive at $N^{- \mu }$, and its derivative is a nonnegative function, it follows that $f_\mu$ is a positive, monotone nondecreasing function in |*x*|.
(e) By definition of $R_\mu$, it follows that ${\tilde{I}}=\int _{{\mathbb {R}}^2}d^2x (V_N(x)-M_\mu (x)) f_\mu (x) =0$. Therefore, for all $|x|\ge R_\mu$, $f_\mu$ solves $-\Delta f_\mu (x)=0$, which has the solution
  $$\begin{aligned} f_\mu (x)= 1 + \frac{{\tilde{I}}}{4 \pi } \ln \left( \frac{|x|}{R_\mu } \right) =1. \end{aligned}$$
(f) Since $f_\mu$ is a positive monotone nondecreasing function in |*x*|, we obtain
  $$\begin{aligned} 1 \ge f_\mu ( N^{- \mu }) =j_{N,R_\mu }( N^{- \mu }) /K_\mu = \left( 1+ \frac{1}{N+ \ln \left( \frac{R_\mu }{a}\right) } \ln \left( \frac{N^{-\mu }}{R_\mu } \right) \right) / K_\mu . \end{aligned}$$
  We obtain the lower bound
  $$\begin{aligned} K_\mu \ge 1+ \frac{1}{N+ \ln \left( \frac{R_\mu }{a}\right) }\ln \left( \frac{N^{-\mu }}{R_\mu } \right) . \end{aligned}$$
  For the upper bound we first prove that $f_{\mu }(x) \ge j_{N,R_\mu }(x)$ holds for all $|x | \le R_\mu$. Using the scatting equations (26) and (30) we obtain
  $$\begin{aligned} \Delta _x ( f_\mu (x)-j_{N,R_\mu }(x) ) = \frac{1}{2} V_N(x) ( f_\mu (x)-j_{N,R_\mu }(x) ) - \frac{1}{2} M_\mu (x) f_{\mu }(x) \end{aligned}$$
  as well as $f_{\mu }(R_\mu )- j_{N,R_\mu }(R_\mu )=0$. Since $M_\mu (x) f_{\mu }(x) \ge 0$, we obtain that $\Delta _x ( f_\mu (x)-j_{N,R_\mu }(x) ) \le 0$ for $N^{-\mu } \le |x|\le R_\mu$. That is, $f_\mu (x)-j_{N,R_\mu }(x)$ is superharmonic for $N^{-\mu }< |x|< R_\mu$. Using the minimum principle, we obtain, using that $f_\mu -j_{N,R_\mu }$ is spherically symmetric
  37 $$\begin{aligned} \min _{N^{-\mu } \le |x|\le R_\mu } ( f_\mu -j_{N,R_\mu }) = \min _{|x| \in \lbrace N^{-\mu }, R_\mu \rbrace } ( f_\mu -j_{N,R_\mu }). \end{aligned}$$
  If it were now that $\min _{|x| \in \lbrace N^{-\mu }, R_\mu \rbrace } ( f_\mu -j_{N,R_\mu }) =f_{\mu }(N^{-\mu })- j_{N,R_\mu }(N^{-\mu }) \le f_{\mu }(R_\mu )- j_{N,R_\mu }(R_\mu )=0$, we could conclude that $f_{\mu }(x)- j_{N,R_\mu }(x) \le 0$ for all $N^{-\mu } \le |x|\le R_\mu$. Since $f_\mu (x)-j_{N,R_\mu }(x)$ then obeys
  $$\begin{aligned} {\left\{ \begin{array}{ll} -\Delta ( f_\mu (x)-j_{N,R_\mu }(x) ) + \frac{1}{2} V_N(x) ( f_\mu (x)-j_{N,R_\mu }(x) ) = 0 \; &{}\text {for } |x| \le N^{-\mu }, \\ f_\mu (x)-j_{N,R_\mu }(x) \le 0 \; &{}\text {for } |x| = N^{-\mu }, \end{array}\right. } \end{aligned}$$
  we could then conclude that $f_\mu (x)-j_{N,R_\mu }(x) \le 0$ for all $|x| \le R_\mu$. From this, we obtain that $\Delta ( f_\mu (x)-j_{N,R_\mu }(x) ) \le 0$ for $|x|\le R_\mu$. That is, $f_\mu (x)-j_{N,R_\mu }(x)$ is superharmonic for all $|x|\le R_\mu$. Using the minimum principle once again, we then obtain
  $$\begin{aligned} \min _{\overline{B_{R_\mu }(0)}} ( f_\mu -j_{N,R_\mu }) = f_\mu (R_\mu )-j_{N,R_\mu }(R_\mu )=0 \end{aligned}$$
  which contradicts $f_{\mu }(x)- j_{N,R_\mu }(x) \le 0$ for $|x| \le R_\mu$. Therefore, we can conclude in (37) that $\min _{N^{-\mu } \le |x|\le R_\mu } ( f_\mu -j_{N,R_\mu }) = f_\mu (R_\mu )-j_{N,R_\mu }(R_\mu )=0$ holds. Then, it follows that $f_\mu (x)-j_{N,R_\mu }(x) \ge 0$ for all $N^{-\mu } \le |x| \le R_\mu$. Using the zero energy scattering equation $-\Delta ( f_\mu (x)-j_{N,R_\mu }(x) ) + \frac{1}{2} V_N(x) ( f_\mu (x)-j_{N,R_\mu }(x) ) = 0$ for $|x| \le N^{-\mu }$, we can, together with $f_\mu (N^{-\mu })-j_{N,R_\mu }(N^{-\mu }) \ge 0$, conclude that $f_{\mu }(x)- j_{N,R_\mu }(x) \ge 0$ for all $|x| \le R_\mu$. As a consequence, we obtain the desired bound $K_\mu = \frac{j_{N,R_\mu }(N^{-\mu })}{f_\mu (N^{-\mu })}\le 1$.
(g) Since $$f_\mu $$ is a nonnegative, monotone nondecreasing function in |*x*| with $$f_\mu (x)=1$$$$\forall |x| \ge R_\mu $$, it follows that
  $$\begin{aligned} C f_\mu (N^{-\mu })&= f_\mu (N^{-\mu }) \int _{{\mathbb {R}}^2} d^2 x V_N(x) \ge \int _{{\mathbb {R}}^2} d^2 x V_N(x) f_\mu (x) \\&= \int _{{\mathbb {R}}^2} d^2 x M_{\mu }(x) f_\mu (x) \ge f_{\mu } (N^{-\mu }) \int _{{\mathbb {R}}^2} d^2 x M_{\mu } (x). \end{aligned}$$
  Therefore, $\int _{{\mathbb {R}}^2} d^2 x M_{\mu } (x) \le C$ holds, which implies that $R_\mu \le CN ^{1/2- \mu }$. From
  $$\begin{aligned} \frac{1}{K_\mu } \frac{4 \pi }{N+ \ln \left( \frac{R_\mu }{a}\right) }&= \frac{1}{K_\mu } \int _{{\mathbb {R}}^2}d^2x V_N(x) j_{N,R_\mu }(x) = \int _{{\mathbb {R}}^2}d^2x V_N(x) f_\mu (x) \\&= \int _{{\mathbb {R}}^2}d^2x M_{\mu }(x) f_\mu (x) = 8 \pi ^2 N^{-1+2 \mu } \int _{N^{-\mu }}^{R_\mu } dr r f_\mu (r) \end{aligned}$$
  we conclude that
  $$\begin{aligned} \int _{N^{-\mu }}^{R_\mu } dr r f_\mu (r) = \frac{N^{1- 2 \mu }}{2 \pi K_\mu \left( N+ \ln \left( \frac{R_\mu }{a} \right) \right) }. \end{aligned}$$
  Since $f_\mu$ is a nonegative, monotone nondecreasing function in |*x*|,
  $$\begin{aligned} \frac{1}{2}( R_\mu ^2- N^{-2 \mu }) \frac{j_{N,R_\mu }(N^{-\mu })}{K_\mu } = \frac{1}{2}( R_\mu ^2- N^{-2 \mu }) f_\mu (N^{-\mu }) \le \int _{N^{-\mu }}^{R_\mu } dr r f_\mu (r) \end{aligned}$$
  which implies
  $$\begin{aligned} R_\mu ^2 N^{2 \mu } \le \frac{N}{\pi \left( N+ \ln \left( \frac{R_\mu }{a} \right) \right) j_{N,R_\mu }(N^{-\mu })}+1. \end{aligned}$$
  Using $R_\mu \le CN ^{1/2- \mu }$, it then follows
  $$\begin{aligned} j_{N,R_\mu }(N^{-\mu }) = 1+ \frac{1}{N+ \ln \left( \frac{R_\mu }{a}\right) } \ln \left( \frac{N^{-\mu }}{R_\mu } \right) \ge 1- \frac{C}{N}, \end{aligned}$$
  which implies $$R_\mu \le CN ^{-\mu }$$.
(h) Using
  $$\begin{aligned} \Vert M_{\mu } f_\mu \Vert _1&= \Vert V_N f_\mu \Vert _1 = K_\mu ^ {-1} \Vert V_N j_{N,R_\mu } \Vert _1 = K_\mu ^{-1} \frac{4 \pi }{N+ \ln \left( \frac{R_\mu }{a}\right) }, \end{aligned}$$
  we obtain
  $$\begin{aligned} |N\Vert V_N f_\mu \Vert _1- 4\pi |&= |N\Vert M_{\mu } f_\mu \Vert _1- 4\pi | = 4 \pi \left| K_\mu ^{-1} \frac{N}{N+ \ln \left( \frac{R_\mu }{a}\right) }-1 \right| \\&= \frac{4 \pi }{K_\mu } \left| \frac{ N-NK_\mu +K_\mu \ln \left( \frac{R_\mu }{a}\right) }{N+ \ln \left( \frac{R_\mu }{a}\right) } \right| \le C \frac{\ln (N)}{N}. \end{aligned}$$
(i) Using for $|x| \le R_\mu$ the inequalities $j_{N,R_\mu }(x) \ge 1+ \frac{1}{N+ \ln \left( \frac{R_\mu }{a}\right) } \ln \left( \frac{|x|}{R_\mu } \right)$ as well as $1\ge f_{\mu }(x) \ge j_{N,R_\mu }(x)$, it follows for $|x| \le R_\mu$
  $$\begin{aligned} 0&\le g_{\mu }(x) = 1- f_{\mu }(x) \le 1- j_{N,R_\mu }(x) \le - \frac{1}{N+ \ln \left( \frac{R_\mu }{a}\right) } \ln \left( \frac{|x|}{R_\mu } \right) \\&\le CN ^ {-1} | \ln \left( N |x| \right) |. \end{aligned}$$
  Since $g_{\mu }(x)=0$ for $\vert x\vert > R_{\mu }$, we conclude with $R_\mu \le CN ^{-\mu }$ that
  $$\begin{aligned} \Vert g_{\mu }\Vert _1&\le \frac{C}{N} \int _{0}^{R_{\mu }}dr r | \ln \left( N r\right) | \le CN ^{-1-2\mu } \ln N, \end{aligned}$$
  as well as
  $$\begin{aligned} \Vert g_{\mu }\Vert ^2&\le \frac{C}{N^2} \int _{0}^{R_{\mu }} dr r \left( \ln \left( Nr \right) \right) ^2 \\&= CN ^{-4} \Big [ r ^2 ( 2 (\ln (r))^2 - 2\ln (r)+1)\Big ]_0^{N R_{\mu }}\\&\le CN ^{-2-2\mu } \left( \ln (N)\right) ^2. \end{aligned}$$
  $\Vert g_{\mu }\Vert _\infty = \Vert 1- f_{\mu }\Vert _\infty \le 1$, since $f_\mu$ is a nonnegative, monotone nondecreasing function with $f_\mu (x) \le 1$.
(j) Using (h) and (i), we obtain with $\Vert M_{\mu }\Vert _1 \le CN ^{-1}$
  $$\begin{aligned} | N \Vert M_{\mu }\Vert _1 - 4 \pi |&\le | N \Vert M_{\mu }f_\mu \Vert _1 - 4 \pi | + N \Vert M_{\mu }g_\mu \Vert _1 \\&\le C \left( \frac{\ln (N)}{N} + \Vert \mathbb {1}_{|\cdot | \ge N^{-\mu }} g_\mu \Vert _\infty \right) . \end{aligned}$$
  Since $g_\mu (x)$ is a nonnegative, monotone nonincreasing function, it follows with $K_\mu \le 1$
  $$\begin{aligned} \Vert \mathbb {1}_{|\cdot | \ge N^{-\mu }} g_\mu \Vert _\infty&= g_\mu (N^{-\mu })=1-f_\mu (N^{-\mu }) = 1- \frac{j_{N,R_\mu }(N^{-\mu })}{K_\mu } \\&\le 1-\left( 1 + \frac{1}{N+ \ln \left( \frac{R_\mu }{a}\right) } \ln \left( \frac{N^{- \mu }}{R_ \mu } \right) \right) . \end{aligned}$$
  and (j) follows.
(k) $M_{\mu } \in \widetilde{{\mathcal {W}}}_\mu$ follows directly from $R_\mu \le CN ^{-\mu }$. From part (j) we then get $b_{M_{\mu }} = 4 \pi$ and $M_{\mu } \in {\mathcal {W}}_\mu$. By means of part (d) we conclude $0 \le M_{\mu } (x) f_\mu (x) \le M_{\mu }(x)$ which together with part (h) implies $M_{\mu } f_\mu \in \widetilde{{\mathcal {W}}}_\mu$, $b_{M_{\mu } f_{\mu }} = 4 \pi$ and $M_{\mu } f_\mu \in {\mathcal {W}}_\mu$. $\square$

## Proof of the Theorem

In this section, we present the proof of Theorem 2.4. We start with the NLS regime and then pursue with the exponential scaling. In both cases we follow the same strategy: After giving the precise definition of the functional we explain its connection to the notion of Bose–Einstein condensation in terms of reduced density matrices. Thereupon, we differentiate the functional with respect to its time variable, perform a Grönwall estimate and finally prove the respective part of the theorem.

### Proof for the NLS scaling $W_\beta$ with $\beta >0$

#### Definition of the functional

The goal of this section is to define a functional $\alpha {:}\,L^2({\mathbb {R}}^{2N},{\mathbb {C}})\times L^2({\mathbb {R}}^2,{\mathbb {C}})\rightarrow {\mathbb {R}}^+_0$ which is adapted to potentials with NLS scaling and which meets all the requirements stated in Sect. 3. In short, we demand the functional to converge to zero for properly chosen initial states and its time derivative to be controllable by means of a Grönwall estimate. Additionally, the functional should allow to prove both Bose–Einstein condensation and the convergence of the energy per particle of the many-body system to the effective energy functional.

While interactions in the mean-field scaling ($W_{\beta }$ with $\beta =0$) become weak for large particle numbers, potentials $W_{\beta }$ with $\beta > 1/2$ are getting peaked as $N\rightarrow \infty$. This fact needs to be taken into account when defining a suitable counting functional. For small $\beta$ and a large class of different choices of the weight ${\widehat{m}}^{\varphi _t}$ with $\varphi _t$ being a solution of (3), it is possible to show that

$$\begin{aligned}&\langle \!\langle \Psi _t,{\widehat{m}}^{\varphi _t}\Psi _t\rangle \!\rangle \le \langle \!\langle \Psi _0,{\widehat{m}}^{\varphi _0}\Psi _0\rangle \!\rangle +\,\int _0^ t ds \\&\quad \left( {\mathcal {K}}(\varphi _s, A_s)\left( \langle \!\langle \Psi _s,{\widehat{m}}^{\varphi _s}\Psi _s\rangle \!\rangle +{\mathcal {O}}(1)+\langle \!\langle \Psi _s,{\widehat{n}}^{\varphi _s}\Psi _s\rangle \!\rangle + \left| {\mathcal {E}}_{W_\beta }(\Psi _s)- {\mathcal {E}}_{b_{W_{\beta }}}^{GP}(\varphi _s) \right| \right) \right) . \end{aligned}$$

This enables us to perform an integral type Grönwall estimate if we choose

$$\begin{aligned} \alpha (\Psi _t,\varphi _t)= \langle \!\langle \Psi _t,{\widehat{n}}^{\varphi _t}\Psi _t\rangle \!\rangle +\left| {\mathcal {E}}_{W_\beta }(\Psi _t)- {\mathcal {E}}_{b_{W_{\beta }}}^{GP}(\varphi _t) \right| . \end{aligned}$$

Here, the smallness of the distance between the energies is used to control the kinetic energy per particle of the many-body system (Lemma 7.6). This prevents the wave function from being strongly localized in the support of the potential and in this way softens the effect of the interaction. Moreover, it allows us to bound the kinetic energy of the particles which are not in the condensate state $\varphi _t$ by $\alpha (\Psi _t,\varphi _t)$, see Lemma 7.9.

For large $$\beta $$, the interaction is harder to control and several estimates break down, if one defines $$\alpha $$ as above. It is therefore necessary to redefine the functional $$\alpha (\Psi _t,\varphi _t)$$ and to carefully choose a new weight function *m*. Let us explain why this is necessary. To obtain an integral type Grönwall estimate, we will calculate the time derivative of $$ \langle \!\langle \Psi _t,{\widehat{m}}^{\varphi _t}\Psi _t\rangle \!\rangle $$. This time derivative will contain contributions of the form $${\widehat{m}}-{\widehat{m}}_1$$ and $${\widehat{m}}-{\widehat{m}}_2$$. To obtain sufficient error estimates for large $$\beta $$, it is necessary to choose a weight function *m* such that $$\Vert {\widehat{m}} - {\widehat{m}}_i\Vert _{\text {op}}$$ with $$i=1, 2 $$ can be controlled sufficiently well (one can infer from the proof below that $$\Vert {\widehat{n}} - {\widehat{n}}_i\Vert _{\text {op}} = {\mathcal {O}}(N^{-1/2})$$ with $$i=1, 2$$ is not decaying sufficiently in *N*, see part (b) of Lemma 7.7). For the Grönwall estimate, we require in addition $$\Vert {\widehat{m}} - {\widehat{n}}\Vert _{\text {op}} \rightarrow 0 $$, as $$N \rightarrow \infty $$.

In total, this suggests the following form of the functional

##### Definition 6.1

For $$0<\xi <\frac{1}{3}$$ define

$$\begin{aligned} m(k)=\left\{ \begin{array}{ll} \sqrt{k/N}, &{}\quad \hbox {for }k\ge N^{1-2\xi }; \\ 1/2(N^{-1+\xi }k+N^{-\xi }), &{}\quad \hbox {else.} \end{array} \right. \end{aligned}$$

and

$$\begin{aligned} \alpha ^<(\Psi ,\varphi )=\langle \!\langle \Psi ,{\widehat{m}}^\varphi \Psi \rangle \!\rangle +\left| {\mathcal {E}}_{W_\beta }(\Psi )- {\mathcal {E}}_{b_{W_{\beta }}}^{GP}(\varphi ) \right| . \end{aligned}$$

##### Remark 6.2

It should be noted, that $\alpha ^<$ depends on the parameter $\xi$ which will be chosen later. For better readability, we disregard the $\xi$ dependence in the notation.

The counting measure can be related to the trace norm distance of the one-particle reduced density matrix.

##### Lemma 6.3

Let $0< \xi < 1/3$, $\Psi \in L^2_s({\mathbb {R}}^{2N},{\mathbb {C}})$, $\varphi \in L^2({\mathbb {R}}^2,{\mathbb {C}})$ and $\alpha ^<(\Psi ,\varphi )$ be defined as in Definition 6.1. Then,

38 $$\begin{aligned} \text {Tr} \left| \gamma ^{(1)}_{\Psi } - |\varphi \rangle \langle \varphi | \right|&\le \sqrt{8 \alpha ^<(\Psi ,\varphi )}, \end{aligned}$$

39 $$\begin{aligned} \alpha ^<(\Psi ,\varphi )&\le \sqrt{\text {Tr} \left| \gamma ^{(1)}_{\Psi } - |\varphi \rangle \langle \varphi | \right| } + \left| {\mathcal {E}}_{W_\beta }(\Psi )- {\mathcal {E}}_{b_{W_{\beta }}}^{GP}(\varphi ) \right| + \frac{1}{2} N^{-\xi }. \end{aligned}$$

##### Proof

We would like to mention, that this Lemma has been proven in [6, Lemma 3.3]. For sake of completeness, we briefly recall the argument. From [30, Lemma2.3] and [50, eq. (6)] one concludes

40 $$\begin{aligned} \left\langle \!\left\langle \Psi , \left( {\widehat{n}}^{\varphi }\right) ^2 \Psi \right\rangle \!\right\rangle&\le \text {Tr} \left| \gamma ^{(1)}_{\Psi } - |\varphi \rangle \langle \varphi | \right| \le \sqrt{8 \left\langle \!\left\langle \Psi , \left( {\widehat{n}}^{\varphi }\right) ^2 \Psi \right\rangle \!\right\rangle }. \end{aligned}$$

If one then uses that $n(k)^2 \le n(k) \le m(k)$ and $m(k) \le n(k) + \frac{1}{2} N^{- \xi }$ imply the relations

$$\begin{aligned} \left\langle \!\left\langle \Psi , \left( {\widehat{n}}^{\varphi }\right) ^2 \Psi \right\rangle \!\right\rangle&\le \langle \!\langle \Psi , {\widehat{m}}^{\varphi } \Psi \rangle \!\rangle \quad \text {and} \quad \langle \!\langle \Psi , {\widehat{m}}^{\varphi } \Psi \rangle \!\rangle \le \sqrt{\left\langle \!\left\langle \Psi , \left( {\widehat{n}}^{\varphi }\right) ^2 \Psi \right\rangle \!\right\rangle } + \frac{1}{2} N^{-\xi }, \end{aligned}$$

the Lemma follows. $\quad \square$

#### Preliminaries for the Grönwall estimate

Subsequently, we will perform a Grönwall estimate for $\alpha ^<$ and prove part (a) of Theorem 2.4. For this, we define

##### Definition 6.4

Let $0< \xi < 1/3$ and $W_\beta \in {\mathcal {W}}_\beta$. Define

41 $$\begin{aligned} Z^\varphi _\beta (x_j,x_k)=W_{\beta }(x_j-x_k) -\frac{N\Vert W_{\beta }\Vert _1}{N-1}|\varphi |^2(x_j)-\frac{N\Vert W_{\beta }\Vert _1}{N-1}|\varphi |^2(x_k). \end{aligned}$$

Note, for $W_\beta (x)= N^{-1+2 \beta } W(N^\beta x)$, we have $N \Vert W_\beta \Vert _1= \Vert W\Vert _1$. With

$$\begin{aligned} m^a(k)=m(k)-m(k+1), \;\;\; m^b(k)=m(k)-m(k+2) \end{aligned}$$

and

$$\begin{aligned} {\widehat{r}}={\widehat{m}}^bp_{1}p_{2}+{\widehat{m}}^a(p_{1}q_{2}+q_1p_2) , \end{aligned}$$

we define for $l \in \{a,b,c \}$ the functionals $\gamma ^<_{l}{:}\,L^2({\mathbb {R}}^{2N},{\mathbb {C}})\times L^2({\mathbb {R}}^2, {\mathbb {C}}) \rightarrow {\mathbb {R}}^+_0$ by

42 $$\begin{aligned} \gamma ^<_a(\Psi ,\varphi )&=\langle \!\langle \Psi ,\dot{A}_t(x_1)\Psi \rangle \!\rangle -\langle \varphi ,\dot{A}_t\varphi \rangle \end{aligned}$$

43 $$\begin{aligned} \gamma ^<_b(\Psi ,\varphi )&=N(N-1)\mathfrak {I}\left( \langle \!\langle \Psi ,Z^\varphi _\beta (x_1,x_2) {\widehat{r}} \Psi \rangle \!\rangle \right) \end{aligned}$$

44 $$\begin{aligned}&=-2N(N-1)\mathfrak {I}\left( \langle \!\langle \Psi ,p_{1}q_{2}{\widehat{m}}^a_{-1}Z^\varphi _\beta (x_1,x_2)p_{1}p_{2} \Psi \rangle \!\rangle \right) \nonumber \\&\quad -\,N(N-1)\mathfrak {I}\left( \langle \!\langle \Psi ,q_{1}q_{2}{\widehat{m}}^b_{-2}W_\beta (x_1-x_2) p_{1}p_{2} \Psi \rangle \!\rangle \right) \nonumber \\&\quad -\,2N(N-1)\mathfrak {I}\left( \langle \!\langle \Psi ,q_{1}q_{2}{\widehat{m}}^a_{-1}Z^\varphi _\beta (x_1,x_2)p_{1}q_{2} \Psi \rangle \!\rangle \right) \end{aligned}$$

45 $$\begin{aligned} \gamma _c^{<}(\Psi , \varphi )&= i N (N \Vert W_{\beta } \Vert _1 - b_{W_{\beta }} ) \langle \!\langle \Psi , (q_1 | \varphi (x_1) |^2 {\widehat{m}}^a p_1 - p_1 {\widehat{m}}^a |\varphi (x_1)|^2 q_1 ) \Psi \rangle \!\rangle . \end{aligned}$$

The value of the functional $\alpha ^<(\Psi _t,\varphi _t)$ at time *t* is then bounded by

##### Lemma 6.5

Let $W_\beta \in {\mathcal {W}}_\beta$. Let $\Psi _t$ the unique solution to $i \partial _t \Psi _t = H_{W_\beta } \Psi _t$ with initial datum $\Psi _0 \in L^2_{s}({\mathbb {R}}^{2N}, {\mathbb {C}}) \cap H^2({\mathbb {R}}^{2N}, {\mathbb {C}}),\; \Vert \Psi _0\Vert =1$. Let $\varphi _t$ the unique solution to $i \partial _t \varphi _t = h^{GP}_{b_{W_{\beta }}} \varphi _t$ with $\varphi _t \in H^3({\mathbb {R}}^2,{\mathbb {C}}) ,\Vert \varphi _0\Vert =1$. Let $\alpha ^<(\Psi _t,\varphi _t)$ be defined as in Definition 6.1. Then

46 $$\begin{aligned} \alpha ^<(\Psi _t,\varphi _t)\le \alpha ^<(\Psi _0,\varphi _0) + \int _0^ t ds \left( \left| \gamma _a^<(\Psi _s,\varphi _s) \right| + \left| \gamma _b^<(\Psi _s,\varphi _s) \right| + \left| \gamma _c^<(\Psi _s,\varphi _s) \right| \right) . \end{aligned}$$

##### Proof

For the proof of the Lemma we restore the upper index $\varphi _t$ in order to pay respect to the time dependence of ${\widehat{m}}^{\varphi _t}$. The time derivative of $\varphi _t$ is given by (3), i.e. $i\partial _t \varphi _t(x_j)= h^{GP}_{b_{W_{\beta }},j} \varphi _t(x_j)$. Here, $h^{GP}_{b_{W_{\beta }},j}$ denotes the operator $h^{GP}_{b_{W_{\beta }}}$ acting on the $j^{\text {th}}$ coordinate $x_j$. We then obtain

$$\begin{aligned} \frac{d}{dt}\langle \!\langle \Psi _t,{\widehat{m}}^{\varphi _t}\Psi _t\rangle \!\rangle&= i\langle \!\langle H_{W_\beta }\Psi _t,{\widehat{m}}^{\varphi _t}\;\Psi _t\rangle \!\rangle - i\langle \!\langle \Psi _t ,{\widehat{m}}^{\varphi _t}\;H_{W_\beta }\Psi _t\rangle \!\rangle \\&\quad -\,i\langle \!\langle \Psi _t ,[\sum _{j=1}^N h_{b_{W_{\beta }},j}^{GP},{\widehat{m}}^{\varphi _t}\;]\Psi _t\rangle \!\rangle \\&= i\langle \!\langle \Psi _t ,[H_{W_\beta }-\sum _{j=1}^N h_{b_{W_{\beta }},j}^{GP},{\widehat{m}}^{\varphi _t}\;]\Psi _t\rangle \!\rangle \\&= i \langle \!\langle \Psi _t, \Big [ \Big ( \frac{1}{2} N (N-1) W_{\beta }(x_1 - x_2) - N b_{W_{\beta }} | \varphi _t(x_1) |^2 \Big ), {\widehat{m}}^{\varphi _t} \;\Big ] \Psi _t \rangle \!\rangle \\&= i N ( N \Vert W_{\beta } \Vert _1 - b_{W_{\beta }} ) \langle \!\langle \Psi _t, [ | \varphi _t(x_1) |^2, {\widehat{m}}^{\varphi _t} \;] \Psi _t \rangle \!\rangle \\&\quad +\, i\frac{N(N-1)}{2}\langle \!\langle \Psi _t ,[Z^{\varphi _t}_\beta (x_1,x_2),{\widehat{m}}^{\varphi _t}\;]\Psi _t\rangle \!\rangle , \end{aligned}$$

where we used the symmetry of $\Psi _t$. Using Lemma 4.2 (d), it follows that (dropping the explicit dependence on $\varphi _t$ from now on)

$$\begin{aligned} \frac{d}{dt}\langle \!\langle \Psi _t,{\widehat{m}}^{\varphi _t}\Psi _t\rangle \!\rangle&= i N (N \Vert W_{\beta } \Vert _1 - b_{W_{\beta }} ) \langle \!\langle \Psi _t, (q_1 | \varphi _t(x_1) |^2 {\widehat{m}}^a p_1 \\&\quad -\,p_1 {\widehat{m}}^a |\varphi _t(x_1)|^2 q_1 ) \Psi _t \rangle \!\rangle \\&\quad +\,\,i\frac{N(N-1)}{2}\langle \!\langle \Psi _t ,[Z^{\varphi _t}_\beta (x_1,x_2),p_1p_2({\widehat{m}}-{\widehat{m}}_2)]\Psi _t\rangle \!\rangle \\&\quad +\,\,i\frac{N(N-1)}{2}\langle \!\langle \Psi _t ,[Z^{\varphi _t}_\beta (x_1,x_2),(p_1q_2+q_1p_2)({\widehat{m}}-{\widehat{m}}_1)]\Psi _t\rangle \!\rangle . \end{aligned}$$

Since $Z^{\varphi _t}_\beta$ and $p_1p_2({\widehat{m}}-{\widehat{m}}_2)$ as well as $p_1q_2({\widehat{m}}-{\widehat{m}}_1)$ are selfadjoint, we obtain

$$\begin{aligned} \frac{d}{dt}&\langle \!\langle \Psi _t,{\widehat{m}}^{\varphi _t}\Psi _t\rangle \!\rangle = \gamma _c^{<}(\Psi _t, \varphi _t) -N(N-1) \\&\quad \times \,\mathfrak {I}\left( \langle \!\langle \Psi _t ,(p_{1}p_{2}+p_{1}q_{2}+q_{1}p_{2}+q_{1}q_{2})Z^{\varphi _t}_\beta (x_1,x_2)( {\widehat{m}}^bp_{1}p_{2}+{\widehat{m}}^a(p_{1}q_{2}+q_1p_2)) \Psi _t\rangle \!\rangle \right) . \end{aligned}$$

Note that in view of Lemma 4.2 (c) ${\widehat{r}}Q_jZ^{\varphi _t}_\beta (x_1,x_2) Q_j= Q_jZ^{\varphi _t}_\beta (x_1,x_2) Q_j{\widehat{r}}$ for any $j\in \{0,1,2\}$ and any weight *r*. Therefore,

$$\begin{aligned} \mathfrak {I}\left( \langle \!\langle \Psi _t,p_{1}p_{2}Z^{\varphi _t}_\beta (x_1,x_2) {\widehat{m}}^bp_{1}p_{2} \Psi _t\rangle \!\rangle \right)&=0\\ \mathfrak {I}\left( \langle \!\langle \Psi _t ,(p_{1}q_{2}+q_{1}p_{2})Z^{\varphi _t}_\beta (x_1,x_2){\widehat{m}}^a(p_{1}q_{2}+q_1p_2) \Psi _t\rangle \!\rangle \right)&=0. \end{aligned}$$

Using Symmetry and Lemma 4.2 (c), we obtain the first line (43). Furthermore,

$$\begin{aligned} \frac{d}{dt}\langle \!\langle \Psi _t,{\widehat{m}}^{\varphi _t}\Psi _t\rangle \!\rangle&= \gamma _c^{<}(\Psi _t, \varphi _t) - 2N(N-1)\mathfrak {I}\left( \langle \!\langle \Psi _t, {\widehat{m}}^b_{-1}p_{1}q_{2}Z^{\varphi _t}_\beta (x_1,x_2)p_{1}p_{2} \Psi _t\rangle \!\rangle \right) \\&\quad -\,N(N-1)\mathfrak {I}\left( \langle \!\langle \Psi _t ,{\widehat{m}}^b_{-2}q_{1}q_{2}Z^{\varphi _t}_\beta (x_1,x_2)p_{1}p_{2} \Psi _t\rangle \!\rangle \right) \\&\quad -\,2N(N-1)\mathfrak {I}\left( \langle \!\langle \Psi _t ,p_{1}p_{2}Z^{\varphi _t}_\beta (x_1,x_2) {\widehat{m}}^ap_{1}q_{2} \Psi _t\rangle \!\rangle \right) \\&\quad -\,2N(N-1)\mathfrak {I}\left( \langle \!\langle \Psi _t ,{\widehat{m}}^a_{-1}q_{1}q_{2}Z^{\varphi _t}_\beta (x_1,x_2)p_{1}q_{2} \Psi _t\rangle \!\rangle \right) . \end{aligned}$$

Since $p_1p_2|\varphi _t^2|(x_1)q_1q_2=p_1p_2q_2|\varphi _t^2|(x_1)q_1=0=p_1p_2|\varphi _t^2|(x_2)q_1q_2$, we can replace $Z^{\varphi _t}_\beta (x_1,x_2)$ in the second line by $W_\beta (x_1-x_2)$.

The third line equals $2N(N-1)\mathfrak {I}\left( \langle \!\langle \Psi _t ,{\widehat{m}}^a p_{1}q_{2}Z^{\varphi _t}_\beta (x_1,x_2) p_{1}p_{2} \Psi _t \rangle \!\rangle \right)$. Since

$$\begin{aligned} m(k-1)-m(k+1)-\left( m(k)-m(k+1)\right) =m(k-1)-m(k) \end{aligned}$$

it follows that ${\widehat{m}}^b_{-1}-{\widehat{m}}^a={\widehat{m}}^a_{-1} - \left( m(0) - m(1) \right) P_0$ and we get

$$\begin{aligned} \frac{d}{dt}\langle \!\langle \Psi _t,{\widehat{m}}^{\varphi _t}\Psi _t\rangle \!\rangle&= \gamma _c^{<}(\Psi _t, \varphi _t) - 2N(N-1)\mathfrak {I}\left( \langle \!\langle \Psi _t ,p_{1}q_{2}{\widehat{m}}^a_{-1}Z^{\varphi _t}_\beta (x_1,x_2)p_{1}p_{2} \Psi _t \rangle \!\rangle \right) \\&\quad -\,N(N-1)\mathfrak {I}\left( \langle \!\langle \Psi _t ,q_{1}q_{2}{\widehat{m}}^b_{-2}W_\beta (x_1-x_2) p_{1}p_{2} \Psi _t \rangle \!\rangle \right) \\&\quad -\,2N(N-1)\mathfrak {I}\left( \langle \!\langle \Psi _t ,q_{1}q_{2}{\widehat{m}}^a_{-1}Z^{\varphi _t}_\beta (x_1,x_2)p_{1}q_{2} \Psi _t \rangle \!\rangle \right) \\&= \gamma _b^{<}(\Psi _t, \varphi _t) + \gamma _c^{<}(\Psi _t, \varphi _t). \end{aligned}$$

For the second summand of $\alpha ^<(\Psi _t,\varphi _t)$ we have

47 $$\begin{aligned} \frac{d}{dt}\left( {\mathcal {E}}_{W_\beta }(\Psi _t)-{\mathcal {E}}_{b_{W_{\beta }}}^{GP} (\varphi _t)\right)&=\langle \!\langle \Psi _t,\dot{A}_t(x_1)\Psi _t\rangle \!\rangle -\langle \varphi _t,\dot{A}_t\varphi _t\rangle \nonumber \\&\quad -\,i \left\langle \varphi _t,\left[ h_{b_{W_{\beta }}}^{GP}, \left( h_{b_{W_{\beta }}}^{GP}-\frac{b_{W_{\beta }}}{2}|\varphi _t|^2\right) \right] \varphi _t\right\rangle \nonumber \\&\quad -\,\left\langle \varphi _t,\frac{b_{W_{\beta }}}{2}\left( \frac{d}{dt}|\varphi _t|^2\right) \varphi _t\right\rangle \nonumber \\&= \langle \!\langle \Psi _t,\dot{A}_t(x_1)\Psi _t\rangle \!\rangle -\langle \varphi _t,\dot{A}_t\varphi _t\rangle \nonumber \\&\quad +\,i \left\langle \varphi _t,\left[ h_{b_{W_{\beta }}}^{GP}, \frac{b_{W_{\beta }}}{2}|\varphi _t|^2\right] \varphi _t\right\rangle \nonumber \\&\quad -\,i \left\langle \varphi _t,\left[ h_{b_{W_{\beta }}}^{GP}, \frac{b_{W_{\beta }}}{2}|\varphi _t|^2\right] \varphi _t\right\rangle \nonumber \\&= \gamma _a^<(\Psi _t,\varphi _t). \end{aligned}$$

By explicit estimates, one can show that the functions $\gamma _{j}^<(\Psi _{\cdot },\varphi _{\cdot }): {\mathbb {R}} \rightarrow {\mathbb {R}}, t \mapsto \gamma _j^<(\Psi _t,\varphi _t)$ with $j \in \{a, b, c \}$ are continuous if $A_{\cdot } \in C^1({\mathbb {R}},L^{\infty }({\mathbb {R}}^2,{\mathbb {R}}))$. The Lemma then follows using that $|f(x)| \le |f(0)|+ \int _0^x dy |f'(y)|$ holds for any $f \in C^1 ({\mathbb {R}}, {\mathbb {R}})$. $\quad \square$

#### The Grönwall estimate

In order to establish a Grönwall estimate for $\alpha ^<$, we have to find a suitable bound for the right hand side of (46).

##### Lemma 6.6

Let $W_\beta \in {\mathcal {W}}_\beta$. Let $\Psi _t$ the unique solution to $i \partial _t \Psi _t = H_{W_\beta } \Psi _t$ with initial datum $\Psi _0 \in L^2_{s}({\mathbb {R}}^{2N}, {\mathbb {C}}) \cap H^2({\mathbb {R}}^{2N}, {\mathbb {C}}), \Vert \Psi _0\Vert =1$. Let $\varphi _t$ the unique solution to $i \partial _t \varphi _t = h^{GP}_{b_{W_{\beta }}} \varphi _t$ with $\varphi _t \in H^{3}({\mathbb {R}}^2,{\mathbb {C}}), \Vert \varphi _0\Vert =1$. Let ${\mathcal {E}}_{W_\beta }(\Psi _0) \le C$.

- Let $\beta < 1/12$. Moreover, let $\alpha ^<(\Psi _t,\varphi _t)$, $\gamma ^<_{a}(\Psi _t,\varphi _t)$ and $\gamma ^<_{b}(\Psi _t,\varphi _t)$ be defined as in Definitions 6.1 and 6.4 with $\xi = 1/6$. Then
  48 $$\begin{aligned} | \gamma _a^<(\Psi _t,\varphi _t) + \gamma _b^<(\Psi _t,\varphi _t) + \gamma _c^<(\Psi _t,\varphi _t) |&\le {\mathcal {K}}(\varphi _t, A_t) \Big ( \alpha ^<(\Psi _t, \varphi _t) + N^{-2 \beta } \ln (N) \Big ). \end{aligned}$$
- Let $\beta \ge 1/12$. Moreover, let $\alpha ^<(\Psi _t,\varphi _t)$, $\gamma ^<_{a}(\Psi _t,\varphi _t)$ and $\gamma ^<_{b}(\Psi _t,\varphi _t)$ be defined as in Definitions 6.1 and 6.4 with $\xi = 1/10$. Then
  49 $$\begin{aligned} | \gamma _a^<(\Psi _t,\varphi _t) + \gamma _b^<(\Psi _t,\varphi _t) + \gamma _c^<(\Psi _t,\varphi _t) |&\le {\mathcal {K}}(\varphi _t, A_t) \Big ( \alpha ^<(\Psi _t, \varphi _t) + N^{-1/10} \Big ). \end{aligned}$$

The proof of Lemma 6.6 is given in Sect. 7.3.

At this point, we only consider the most relevant term $\gamma _b^<(\Psi _t,\varphi _t)$ and explain on a heuristic level why it is small. The principle argument follows the ideas and estimates of [49]. The first line in (44) is the most important one. This expression is only small if the correct coupling parameter $b_{W_{\beta }} \approx N \Vert W_\beta \Vert _1$ is used in the mean-field equation (3). Then,

$$\begin{aligned} N p_{1}^{\varphi _t} W_\beta (x_1-x_2)p_1^{\varphi _t} =N p_1^{\varphi _t} W_\beta *|\varphi |^2(x_2)p_1^{\varphi _t} \rightarrow p_1^{\varphi _t} |\varphi |^2(x_2) \Vert W\Vert _1 p_1^{\varphi _t} \end{aligned}$$

converges against the mean-field potential, and hence the first expression of (44) is small.

In order to estimate the second and third line of (44), one tries to bound $N^2\langle \!\langle \Psi _t ,q_{1}^{\varphi _t} q_{2}^{\varphi _t} {\widehat{m}}^b_{-2}W_\beta (x_1-x_2) p_{1}^{\varphi _t} p_{2}^{\varphi _t} \Psi _t \rangle \!\rangle$ and $N^2\langle \!\langle \Psi _t ,q_{1}^{\varphi _t}q_{2}^{\varphi _t} {\widehat{m}}^a_{-1}Z^{\varphi }_\beta (x_1-x_2) p_{1}^{\varphi _t} q_{2}^{\varphi _t} \Psi _t \rangle \!\rangle$ in terms of $\langle \!\langle \Psi _t, {\widehat{n}}^{\varphi _t} \Psi _t \rangle \!\rangle + {\mathcal {O}}(N^{-\eta })$ for some $\eta >0$. By means of

$$\begin{aligned} | \langle \!\langle \Psi _t,{\widehat{n}}^{\varphi _t}\Psi _t\rangle \!\rangle - \langle \!\langle \Psi _t,{\widehat{m}}^{\varphi _t}\Psi _t\rangle \!\rangle | \le \Vert {\widehat{n}}^{\varphi _t}-{\widehat{m}}^{\varphi _t}\Vert _{\text {op}} = N^{-\xi } \end{aligned}$$

this can then be bounded by $\alpha ^<(\Psi _t,\varphi _t) + {\mathcal {O}}(N^{-\eta })$ for some $\eta >0$.

With the help of Lemma 6.5, Lemma 6.6 and Grönwall’s Lemma, we obtain

##### Lemma 6.7

Let $W_\beta \in {\mathcal {W}}_\beta$. Let $\Psi _t$ the unique solution to $i \partial _t \Psi _t = H_{W_\beta } \Psi _t$ with initial datum $\Psi _0 \in L^2_{s}({\mathbb {R}}^{2N}, {\mathbb {C}}) \cap H^2({\mathbb {R}}^{2N}, {\mathbb {C}}),\; \Vert \Psi _0\Vert =1$. Let $\varphi _t$ the unique solution to $i \partial _t \varphi _t = h^{GP}_{b_{W_{\beta }}} \varphi _t$ with $\varphi _t \in H^3({\mathbb {R}}^2,{\mathbb {C}}),\Vert \varphi _0\Vert =1$.

- Let $\beta < 1/12$ and $\alpha ^<(\Psi _t,\varphi _t)$ be defined as in Definition 6.1 with $\xi = 1/6$. Then,
  50 $$\begin{aligned} \alpha ^<(\Psi _t,\varphi _t)&\le e^{\int _0^t ds \, {\mathcal {K}}(\varphi _s, A_s) } \big ( \alpha ^<(\Psi _0,\varphi _0) + N^{-2 \beta } \ln (N) \big ). \end{aligned}$$
- Let $\beta \ge 1/12$ and $\alpha ^<(\Psi _t,\varphi _t)$ be defined as in Definition 6.1 with $\xi = 1/10$. Then,
  51 $$\begin{aligned} \alpha ^<(\Psi _t,\varphi _t)&\le e^{\int _0^t ds \, {\mathcal {K}}(\varphi _s, A_s) } \big ( \alpha ^<(\Psi _0,\varphi _0) + N^{-1/10} \big ). \end{aligned}$$

##### Proof

From Lemmas 6.5 and 6.6, we have

$$\begin{aligned} \alpha ^<(\Psi _t,\varphi _t)&\le \alpha ^<(\Psi _0,\varphi _0) + \int _0^t ds \, {\mathcal {K}}(\varphi _s, A_s) \big ( \alpha ^<(\Psi _s,\varphi _s) + N^{-2 \beta } \ln (N) \big ) \end{aligned}$$

in the case of $\beta < 1/12$. Thus if we apply Grönwall’s Lemma, we get

$$\begin{aligned} \alpha ^<(\Psi _s,\varphi _s)&\le \alpha ^<(\Psi _0,\varphi _0) + \int _0^t ds \, {\mathcal {K}}(\varphi _s, A_s) N^{-2 \beta } \ln (N) \\&\quad +\,\int _0^t ds \, {\mathcal {K}}(\varphi _s, A_s) e^{\int _s^t d\tau \, {\mathcal {K}}(\varphi _{\tau }, A_{\tau })} \Big ( \alpha ^<(\Psi _0,\varphi _0) \\&\qquad +\,\int _0^s du \, {\mathcal {K}}(\varphi _u, A_u) N^{- 2 \beta } \ln (N) \Big ). \end{aligned}$$

With the help of the relation $|x| \le e^{|x|}$ this can be further simplified and one obtains (50). Part (b) of the Lemma is shown in complete analogy. $\quad \square$

##### Proof of Theorem 2.4: Part (a)

Note that under the assumptions $\varphi _t \in H^{3}({\mathbb {R}}^2,{\mathbb {C}})$ and $A_{\cdot } \in C^1({\mathbb {R}},L^{\infty }({\mathbb {R}}^2,{\mathbb {R}}))$ there exists a constant $C_t < \infty$, depending on *t*, $\varphi _0$ and $A_t$, such that $\int _0^t ds {\mathcal {K}}(\varphi _s, A_s) \le C_t$, see Sect. 4. Let $\beta < 1/12$ and $\xi = 1/6$. We now combine Lemmas 6.3 and 6.7 to estimate

$$\begin{aligned} \text {Tr} \left| \gamma ^{(1)}_{\Psi _t} - |\varphi _t \rangle \langle \varphi _t| \right|&\le C \sqrt{\alpha ^<(\Psi _t,\varphi _t)} \le e^{C_t} \sqrt{\alpha ^<(\Psi _0,\varphi _0) + N^{-2 \beta } \ln (N)} \\&\le e^{C_t} \Bigg ( \root 4 \of {\text {Tr} \left| \gamma ^{(1)}_{\Psi _0} - |\varphi _0 \rangle \langle \varphi _0| \right| } + \sqrt{\left| {\mathcal {E}}_{W_\beta }(\Psi _0)- {\mathcal {E}}_{b_{W_{\beta }}}^{GP}(\varphi _0) \right| } \\&\quad +\,N^{-\beta } \sqrt{\ln (N)} \Bigg ). \end{aligned}$$

Here, we have used $N^{-1/6} \le N^{-2 \beta } \ln (N)$ and $\sqrt{|a| + |b|} \le \sqrt{|a|} + \sqrt{|b|}$ to obtain the last line. In a similar way, one shows

$$\begin{aligned} \left| {\mathcal {E}}_{W_\beta }(\Psi _t)- {\mathcal {E}}_{b_{W_{\beta }}}^{GP}(\varphi _t) \right|&\le \alpha ^<(\Psi _t,\varphi _t) \\&\le e^{C_t} \Bigg ( \sqrt{\text {Tr} \left| \gamma ^{(1)}_{\Psi _0} - |\varphi _0 \rangle \langle \varphi _0| \right| } + \left| {\mathcal {E}}_{W_\beta }(\Psi _0)- {\mathcal {E}}_{b_{W_{\beta }}}^{GP}(\varphi _0) \right| \\&\quad +\,N^{- 2 \beta } \ln (N) \Bigg ). \end{aligned}$$

In total, this shows part (a) of Theorem 2.4 for $\beta < 1/12$. The estimates for $\beta \ge 1/12$ are shown in exactly the same manner. $\quad \square$

### Proof for the exponential scaling $V_N$

#### Definition of the functional

In case of the exponential scaling, the interaction is so strong such that the many-body wave function develops a non-negligible short scale correlation structure which prevents the particles from being localized too close to each other. These correlations determine the statical and dynamical properties of the condensate in a crucial manner and need to be taken into account explicitly. It is therefore reasonable to expect that the counting measure needs to be modified, too.

In order to motivate how the correlation structure will appear in the definition of the functional we think for the moment of the most simple counting measure, namely $\langle \!\langle \Psi _t, q_1^{\varphi _t} \Psi _t \rangle \!\rangle = 1 - \langle \!\langle \Psi _t, p_1^{\varphi _t} \Psi _t \rangle \!\rangle$. This functional counts the relative number of particles which are not in the state $\varphi _t$ and consequently measures if the many-body state is approximately given by the product state $\varphi _t^{\otimes N}$, in the sense of reduced density matrices. However, in the face of the exponential scaling, one should picture the many-body state not as the product of one-particle states but rather as a wave function of Jastrow-type, i.e.

$$\begin{aligned} \Psi _t(x_1, \ldots , x_N)&\approx \prod _{1 \le i< j \le N} j_{N,R}(x_i - x_j) \prod _{k=1}^N \varphi _t(x_k) \\&= \prod _{l=2}^N j_{N,R}(x_1 - x_l) \varphi _t(x_1) \Big ( \prod _{2 \le i < j \le N} j_{N,R}(x_i - x_j) \prod _{k=1}^N \varphi _t(x_k) \Big ) \end{aligned}$$

with $j_{N,R}$ being the zero energy scattering state as defined in (26).

In the following, we will consider the correlation structure to be induced by $f_{\mu }$ (see Definition 5.3) rather than by $j_{N,R}$. This replacement does not change the heuristic discussion above, since $f_{\mu }(x) \approx j_{N,R_\mu }(x) \; \forall |x| \le N^{-\mu }$ for *N* large (see Lemma 5.5), but will allow us to smoothen the singular interaction, as we will explain in the following.

Instead of projecting onto the state $\varphi _t$, the previous discussion suggests to replace $p_1^{\varphi _t}$ by $|\prod _{k=2}^N f_{\mu }(x_1 - x_k) \varphi _t(x_1) \rangle \langle \prod _{l=2}^Nf_{\mu }(x_1 - x_l) \varphi _t(x_1)|$. The counting measure would then be given by

$$\begin{aligned}&1 - \left\langle \!\left\langle \Psi _t, |\prod _{k=2}^Nf_{\mu }(x_1 - x_k) \varphi _t(x_1) \rangle \langle \prod _{l=2}^Nf_{\mu }(x_1 - x_l) \varphi _t(x_1)| \Psi _t\right\rangle \!\right\rangle \\&\quad = 1 - \left\langle \!\left\langle \Psi _t, \prod _{k=2}^N f_{\mu }(x_1 - x_k) p_1^{\varphi _t} \prod _{l=2}^N f_{\mu }(x_1 - x_l) \Psi _t\right\rangle \!\right\rangle . \end{aligned}$$

This expression can be further simplified, if we use $g_{\mu } = 1 - f_{\mu }$ and only keep the terms which are at most linear in $g_{\mu }$

52 $$\begin{aligned}&1 - \langle \!\langle \Psi _t, \Big ( 1- \sum _{k=2}^N g_{\mu }(x_1 - x_k) \Big ) p_1^{\varphi _t} \Big ( 1- \sum _{l=2}^N g_{\mu }(x_1 - x_l) \Big ) \Psi _t\rangle \!\rangle \nonumber \\&\quad \approx 1 - \langle \!\langle \Psi _t, p_1^{\varphi _t} \Psi _t \rangle \!\rangle + 2 (N-1) \mathfrak {R}\langle \!\langle \Psi _t, g_{\mu }(x_1 - x_2) p_1^{\varphi } \Psi _t \rangle \!\rangle \nonumber \\&\quad = \langle \!\langle \Psi _t, q_1^{\varphi _t} \Psi _t \rangle \!\rangle + 2 (N-1) \mathfrak {R}\langle \!\langle \Psi _t, g_{\mu }(x_1 - x_2) p_1^{\varphi } \Psi _t \rangle \!\rangle . \end{aligned}$$

With the help of the symmetry of the many-body wave function and the identity $q_1^{\varphi _t} = 1 - p_1^{\varphi _t}$, we compute

$$\begin{aligned} \frac{d}{dt} \langle \!\langle \Psi _t, q_1^{\varphi _t} \Psi _t \rangle \!\rangle&= 2 \mathfrak {I}\Big ( \langle \!\langle \Psi _t, \big ( (N-1) V_{N}(x_1-x_2) - 4 \pi | \varphi _t(x_1) |^2 \big ) p_1^{\varphi _t} \Psi _t \rangle \!\rangle \Big ). \end{aligned}$$

Defining $h_{4 \pi }^{GP}(x_1) = \left( -\Delta _1 +A_t(x_1)\right) + 4 \pi |\varphi _t(x_1)|^2$, we further compute

$$\begin{aligned}&\frac{d}{dt} 2 (N-1) \mathfrak {R}\Big ( \langle \!\langle \Psi _t, g_{\mu }(x_1- x_2) p_1^{\varphi _t} \Psi _t \rangle \!\rangle \Big ) \\&\quad = - 2 (N-1) \mathfrak {I}\Big ( \langle \!\langle \Psi _t, \big [ H_{V_N}, g_{\mu }(x_1-x_2) \big ]p_1^{\varphi _t} \Psi _t \rangle \!\rangle \Big ) \\&\qquad -\,2 (N-1) \mathfrak {I}\Big ( \langle \!\langle \Psi _t, g_{\mu }(x_1 -x_2) \Big [ \big ( H_{V_N} -h_{4 \pi }^{GP}(x_1) \big ), p_1^{\varphi _t} \Big ] \Psi _t \rangle \!\rangle \Big ). \end{aligned}$$

Using (30) and neglecting the mixed derivatives we get that $[H_{V_N}, g_{\mu }(x_1-x_2)] \approx (V_N - M_{\mu })(x_1-x_2) f_{\mu }(x_1- x_2)$. Further one can show that the leading order of $g_{\mu }(x_1 -x_2) \Big [ \big ( H_{V_N} -h_{4 \pi }^{GP}(x_1) \big ), p_1^{\varphi _t} \Big ]$ is given by $g_{\mu }(x_1 -x_2) V_N(x_1 -x_2) p_1^{\varphi _t}$. This is due to the smallness of the support of $g_{\mu }$ and $V_N$ which significantly overlap only for this term.

Hence the leading order of $\frac{d}{dt}$ (52) is given by

53 $$\begin{aligned} 2 \mathfrak {I}\Big ( \langle \!\langle \big ( (N-1) M_{\mu }(x_1-x_2) f_{\mu }(x_1-x_2) - 4 \pi |\varphi _t(x_1)|^2 \big ) p_1^{\varphi _t} \Psi _t \rangle \!\rangle \Big ). \end{aligned}$$

Summarizing we can say that due to this adjustment and by means of the scattering equation (30), the interaction $V_N$ got replaced by the less singular potential $M_{\mu }f_{\mu }$ in in the first line of the equation above. It is this less singular potential that can be controlled using the results from the previous chapter. $M_{\mu }f_{\mu }$ has the properties of the $W_\beta$ considered above [see Lemma 5.5 (k)], making (53) controllable. This explains why we chose to use $f_{\mu }$ in the definition of the modified counting functional instead of $j_{N, R}$. In return we obtain additional error terms, which, however, can be estimated sufficiently well, see Lemma 6.13.

Making use of Lemma 4.2 (c) and (d) this idea can also be used for weight functions different from $\frac{k}{N}$. Note that due to symmetry the correction term in (52) can be written as

$$\begin{aligned}&2 (N-1) \mathfrak {R}\langle \!\langle \Psi _t, g_{\mu }(x_1 - x_2) p_1^{\varphi } \Psi _t \rangle \!\rangle \\&\quad =-N(N-1)\mathfrak {R}\langle \!\langle \Psi _t, g_{\mu }(x_1 - x_2) \left( -N^{-1}\right) \left( p_1^{\varphi }q_2^{\varphi }+q_1^{\varphi }p_2^{\varphi } \right) \Psi _t \rangle \!\rangle \\&\quad -\,N(N-1)\mathfrak {R}\langle \!\langle \Psi _t, g_{\mu }(x_1 - x_2) \left( - 2 N^{-1}\right) p_1^{\varphi }p_2^{\varphi } \Psi _t \rangle \!\rangle . \end{aligned}$$

Moreover, $N^{-1}$ can be viewed as the discrete time derivative of the weight $\frac{k}{N}$, in other words

$$\begin{aligned} -N^{-1}= & {} \frac{k}{N}-\frac{k+1}{N} = n^2(k) - n^2(k+1) \quad \text {and} \quad \\ -2N^{-1}= & {} \frac{k}{N}-\frac{k+2}{N} = n^2(k) - n^2(k+2). \end{aligned}$$

We will use this insight to modify the functional $\alpha ^<(\Psi _t, \varphi _t)$ from Definition 6.1. We first compute the time derivative of $\langle \!\langle \Psi _t, {\widehat{m}}^{\varphi _t} \Psi _t \rangle \!\rangle$ and then add an additional term to the counting measure in a way such that the interaction $V_N$ gets replaced by the potential $M_{\mu } f_{\mu }$.

Pursuing this approach results in the following definition.

##### Definition 6.8

Let $0<\xi <\frac{1}{3}$, $\mu >0$ and *m*(*k*) be defined as in Definition 6.1. Moreover, let

54 $$\begin{aligned} m^a(k)&=m(k)-m(k+1), \end{aligned}$$

55 $$\begin{aligned} m^b(k)&= m(k)-m(k+2) \quad \text {and} \end{aligned}$$

56 $$\begin{aligned} {\widehat{r}}&={\widehat{m}}^b p_{1}p_{2}+{\widehat{m}}^a(p_{1}q_{2}+q_1p_2). \end{aligned}$$

Then, $\alpha {:}\,L^2({\mathbb {R}}^{2N},{\mathbb {C}})\times L^2({\mathbb {R}}^2,{\mathbb {C}})\rightarrow {\mathbb {R}}^+_0$ is defined by

57 $$\begin{aligned} \alpha (\Psi ,\varphi )&= \langle \!\langle \Psi , {\widehat{m}} \Psi \rangle \!\rangle + \left| {\mathcal {E}}_{V_N} (\Psi ) - {\mathcal {E}}^{GP}_{4 \pi } (\varphi ) \right| -N(N-1)\mathfrak {R}\left( \langle \!\langle \Psi , g_{\mu }(x_{1}-x_{2}) {\widehat{r}} \Psi \rangle \!\rangle \right) . \end{aligned}$$

##### Remark 6.9

It should be noted that ${\widehat{r}}$ depends on $\xi$ and the functional $\alpha$ depends on $\xi$ and $\mu$. Both parameters are later chosen in a way such that we can establish an integral type Grönwall estimate.

If one recalls Definition 6.3, one sees that $\alpha$ is obtained from $\alpha ^<$ by adding an additional correction term. It is important to note that (see proof of Lemma 6.10)

58 $$\begin{aligned} N(N-1)|\mathfrak {R}\left( \left\langle \!\left\langle \Psi , g_{\mu }(x_{1}-x_{2}) {\widehat{r}} \Psi \right\rangle \!\right\rangle \right) | \le C \Vert \varphi \Vert _{\infty } N^{-\mu +\xi } \ln (N). \end{aligned}$$

For $\mu$ chosen large enough this allows us to show that the convergence of $\alpha$ to zero can be related to the notion of complete Bose–Einstein condensation in terms of reduced density matrices.

##### Lemma 6.10

Let $0< \xi < 1/3$, $\mu >0$, $\Psi \in L^2_s({\mathbb {R}}^{2N},{\mathbb {C}})$, $\varphi \in L^2({\mathbb {R}}^2,{\mathbb {C}}) \cap L^{\infty }({\mathbb {R}}^2,{\mathbb {C}})$ and $\alpha (\Psi ,\varphi )$ be defined as in Definition 6.1. Then, there exists a constant $C \in (0, \infty )$ such that

59 $$\begin{aligned} \text {Tr} \left| \gamma ^{(1)}_{\Psi } - |\varphi \rangle \langle \varphi | \right|&\le \sqrt{8 \alpha (\Psi ,\varphi )} + C \Vert \varphi \Vert _{\infty } N^{-1/2(\mu - \xi )} \sqrt{\ln (N)}, \end{aligned}$$

60 $$\begin{aligned} \alpha (\Psi ,\varphi )&\le \sqrt{\text {Tr} \left| \gamma ^{(1)}_{\Psi } - |\varphi \rangle \langle \varphi | \right| } + \left| {\mathcal {E}}_{V_N} (\Psi ) - {\mathcal {E}}^{GP}_{4 \pi } (\varphi ) \right| \nonumber \\&\quad +\,\frac{1}{2} N^{-\xi } + C \Vert \varphi \Vert _{\infty } N^{- \mu + \xi } \ln (N). \end{aligned}$$

##### Proof

Using $\Vert {\widehat{m}}^a\Vert _{\text {op}} + \Vert {\widehat{m}}^b\Vert _{\text {op}} \le CN ^{-1+\xi }$, see (76), together with Eq. (16) and Lemma 5.5 (i), we obtain

$$\begin{aligned} \Vert g_{\mu }(x_{1}-x_{2}) {\widehat{r}}\Vert _{\text {op}}&\le \Vert g_{\mu }(x_{1}-x_{2})p_1( {\widehat{m}}^b p_2+ {\widehat{m}}^a q_2) \Vert _{\text {op}} + \Vert g_{\mu }(x_{1}-x_{2}) p_2 q_1 {\widehat{m}}^a\Vert _{\text {op}} \\&\le \Vert \varphi \Vert _{\infty } \Vert g_{\mu } \Vert ( \Vert {\widehat{m}}^a\Vert _{\text {op}} + \Vert {\widehat{m}}^b\Vert _{\text {op}} ) \\&\le \Vert \varphi \Vert _{\infty } N^{\xi -2- \mu } \ln (N). \end{aligned}$$

Therefore, we bound $N(N-1)|\mathfrak {R}\left( \langle \!\langle \Psi , g_{\mu }(x_{1}-x_{2}) {\widehat{r}} \Psi \rangle \!\rangle \right) | \le \Vert \varphi \Vert _{\infty } N^{-\mu +\xi } \ln (N).$ By means of Lemma 6.3 the Lemma follows. $\quad \square$

#### Preliminaries for the Grönwall estimate

##### Definition 6.11

Let $0< \xi < 1/3$, $\mu >0$ and ${\widehat{r}}$ be defined as in Definition 6.8. Then, $\gamma {:}\,L^2({\mathbb {R}}^{2N},{\mathbb {C}})\times L^2({\mathbb {R}}^2,{\mathbb {C}})\rightarrow {\mathbb {R}}$ is defined by

61 $$\begin{aligned} \gamma (\Psi ,\varphi )=|\gamma _a(\Psi ,\varphi )|+|\gamma _b(\Psi ,\varphi )|+|\gamma _c(\Psi ,\varphi )|+|\gamma _d(\Psi ,\varphi )|+|\gamma _e(\Psi ,\varphi )| + |\gamma _f (\Psi , \varphi )|, \end{aligned}$$

where the different summands are:

- The change in the energy-difference
  $$\begin{aligned} \gamma _a(\Psi ,\varphi )=\langle \!\langle \Psi ,\dot{A}_t(x_1)\Psi \rangle \!\rangle -\langle \varphi ,\dot{A}_t\varphi \rangle . \end{aligned}$$
- The new interaction term
  $$\begin{aligned}\gamma _b(\Psi ,\varphi )&=-N(N-1)\mathfrak {I}\left( \langle \!\langle \Psi , {\widetilde{Z}}_{\mu }^\varphi (x_1,x_2){\widehat{r}}\, \Psi \rangle \!\rangle \right) \\ {}&\quad -\, N(N-1)\mathfrak {I}\left( \langle \!\langle \Psi ,g_{\mu }(x_{1}-x_{2}) {\widehat{r}}\,{\mathcal {Z}}^\varphi (x_1,x_2) \Psi \rangle \!\rangle \right) , \end{aligned}$$
  where, using $M_{\mu }$ from Definition 5.3,
  62 $$\begin{aligned}&{\widetilde{Z}}_{\mu }^\varphi (x_1,x_2)=\left( M_{\mu }(x_1-x_2) - 4 \pi \frac{|\varphi |^2(x_1)+|\varphi |^2(x_2)}{N-1}\right) f_{\mu }(x_{1}-x_{2}) \nonumber \\&{\mathcal {Z}}^\varphi (x_1,x_2)= V_N(x_1-x_2)- \frac{4 \pi }{N-1} |\varphi |^ 2(x_1) - \frac{4 \pi }{N-1} |\varphi |^ 2(x_2). \end{aligned}$$
- The mixed derivative term
  $$\begin{aligned} \gamma _c(\Psi ,\varphi )&=-4N(N-1)\langle \!\langle \Psi , (\nabla _1g_{\mu }(x_1-x_2))\nabla _1 {\widehat{r}}\Psi \rangle \!\rangle . \end{aligned}$$
- Three particle interactions
  $$\begin{aligned} \gamma _d(\Psi ,\varphi )&=2N(N-1)(N-2)\mathfrak {I}\left( \langle \!\langle \Psi ,g_{\mu }(x_{1}-x_{2}) \left[ V_N(x_1-x_3), {\widehat{r}}\right] \Psi \rangle \!\rangle \right) \\&\quad -\,N(N-1)(N-2)\mathfrak {I}\left( \langle \!\langle \Psi ,g_{\mu }(x_{1}-x_{2}) \left[ 4 \pi |\varphi |^2(x_3), {\widehat{r}}\right] \Psi \rangle \!\rangle \right) . \end{aligned}$$
- Interaction terms of the correction
  $$\begin{aligned}&\gamma _e(\Psi ,\varphi )=\frac{1}{2}N(N-1)(N-2)(N-3) \mathfrak {I}\left( \langle \!\langle \Psi , g_{\mu }(x_{1}-x_{2})\left[ V_N(x_3-x_4), {\widehat{r}}\right] \Psi \rangle \!\rangle \right) . \end{aligned}$$
- Correction terms of the mean field
  $$\begin{aligned} \gamma _f(\Psi ,\varphi )= -2N (N-2)\mathfrak {I}\left( \langle \!\langle \Psi ,g_{\mu }(x_{1}-x_{2}) \left[ 4 \pi |\varphi |^2(x_1), {\widehat{r}}\right] \Psi \rangle \!\rangle \right) . \end{aligned}$$

The value of $\alpha (\Psi _t,\varphi _t)$ at time *t* is then bounded by

##### Lemma 6.12

Let $V_N \in {\mathcal {V}}_N$ and let $\Psi _t$ the unique solution to $i \partial _t \Psi _t = H_{V_N} \Psi _t$ with initial datum $\Psi _0 \in L^2_{s}({\mathbb {R}}^{2N}, {\mathbb {C}}) \cap H^2({\mathbb {R}}^{2N}, {\mathbb {C}}), \Vert \Psi _0\Vert =1$. Let $\varphi _t$ the unique solution to $i \partial _t \varphi _t = h^{GP}_{4 \pi } \varphi _t$ with $\varphi _t \in H^3({\mathbb {R}}^2,{\mathbb {C}}),\Vert \varphi _0\Vert =1$. Let $\alpha (\Psi _t,\varphi _t)$ and $\gamma (\Psi _t,\varphi _t)$ be defined as in (57) and (61). Then

$$\begin{aligned} \alpha (\Psi _t,\varphi _t)\le \alpha (\Psi _0,\varphi _0) + \int _0^t ds \gamma (\Psi _s,\varphi _s). \end{aligned}$$

##### Proof

We first calculate

$$\begin{aligned}&\frac{\text {d}}{\text {d}t}\left( \langle \!\langle \Psi , {\widehat{m}} \Psi \rangle \!\rangle -N(N-1)\mathfrak {R}\left( \langle \!\langle \Psi , g_{\mu }(x_{1}-x_{2}) {\widehat{r}} \Psi \rangle \!\rangle \right) \right) \\&\quad = -N(N-1)\mathfrak {I}\left( \langle \!\langle \Psi _t,{\mathcal {Z}}^{\varphi _t}(x_1,x_2) {\widehat{r}} \Psi _t\rangle \!\rangle \right) \\&\qquad -\,N(N-1)\mathfrak {R}\left( i\langle \!\langle \Psi _t, g_{\mu }(x_{1}-x_{2})\left[ H_{V_N}-\sum _{i=1}^N h^{GP}_{4 \pi ,i}, {\widehat{r}}\right] \Psi _t\rangle \!\rangle \right) \\&\qquad -\,N(N-1)\mathfrak {R}\left( i\langle \!\langle \Psi _t,\left[ H_{V_N}, g_{\mu }(x_{1}-x_{2}) \right] {\widehat{r}} \Psi _t\rangle \!\rangle \right) . \end{aligned}$$

Using symmetry and $\mathfrak {R}(iz)=-\mathfrak {I}(z)$, we obtain

$$\begin{aligned}&\frac{\text {d}}{\text {d}t}\left( \langle \!\langle \Psi , {\widehat{m}} \Psi \rangle \!\rangle -N(N-1)\mathfrak {R}\left( \langle \!\langle \Psi , g_{\mu }(x_{1}-x_{2}) {\widehat{r}} \Psi \rangle \!\rangle \right) \right) \\&\quad = -N(N-1)\mathfrak {I}\left( \langle \!\langle \Psi _t,{\mathcal {Z}}^{\varphi _t}(x_1,x_2) {\widehat{r}} \Psi _t\rangle \!\rangle \right) \\&\qquad +\,N(N-1)\mathfrak {I}\left( \langle \!\langle \Psi _t ,g_{\mu }(x_{1}-x_{2})\left[ {\mathcal {Z}}^{\varphi _t}(x_1,x_2), {\widehat{r}}\right] \Psi _t\rangle \!\rangle \right) \\&\qquad +\,2N(N-1)(N-2)\mathfrak {I}\left( \langle \!\langle \Psi _t,g_{\mu }(x_{1}-x_{2}) \left[ V_N(x_1-x_3), {\widehat{r}}\right] \Psi _t\rangle \!\rangle \right) \\&\qquad -\,N(N-1)(N-2)\mathfrak {I}\left( \langle \!\langle \Psi _t,g_{\mu }(x_{1}-x_{2}) \left[ 4 \pi |\varphi _t|^2(x_3), {\widehat{r}}\right] \Psi _t\rangle \!\rangle \right) \\&\qquad +\,\frac{1}{2}N(N-1)(N-2)(N-3)\mathfrak {I}\left( \langle \!\langle \Psi _t, g_{\mu }(x_{1}-x_{2})\left[ V_N(x_3-x_4), {\widehat{r}}\right] \Psi _t\rangle \!\rangle \right) \\&\qquad +\,N(N-1)\mathfrak {I}\left( \langle \!\langle \Psi _t,\left[ H_{V_N}, g_{\mu }(x_{1}-x_{2}) \right] {\widehat{r}} \Psi _t\rangle \!\rangle \right) . \\&\qquad -\, 2 N(N-2)\mathfrak {I}\left( \langle \!\langle \Psi _t,g_{\mu }(x_{1}-x_{2}) \left[ 4 \pi |\varphi _t|^2(x_1), {\widehat{r}}\right] \Psi _t\rangle \!\rangle \right) . \end{aligned}$$

The third and fourth lines equal $\gamma _d$ (recall that $\Psi$ is symmetric), the fifth line equals $\gamma _e$ and the seventh line equals $\gamma _f$. Using that $(1-g_{\mu }(x_{1}-x_{2})){\mathcal {Z}}^\varphi (x_1,x_2)={\widetilde{Z}}_{\mu }^\varphi (x_1,x_2)+(V_{N}(x_1-x_2)-M_{\mu }(x_1-x_2))f_{\mu }(x_{1}-x_{2})$ we get

63 $$\begin{aligned}&\frac{\text {d}}{\text {d}t}\left( \langle \!\langle \Psi , {\widehat{m}} \Psi \rangle \!\rangle -N(N-1)\mathfrak {R}\left( \langle \!\langle \Psi , g_{\mu }(x_{1}-x_{2}) {\widehat{r}} \Psi \rangle \!\rangle \right) \right) \nonumber \\&\quad \le \gamma _d(\Psi _t,\varphi _t)+\gamma _e(\Psi _t,\varphi _t)+\gamma _f(\Psi _t,\varphi _t) \nonumber \\&\qquad -\,N(N-1)\mathfrak {I}\left( \langle \!\langle \Psi _t,{\widetilde{Z}}_{\mu }^{\varphi _t}(x_1,x_2) {\widehat{r}} \Psi _t\rangle \!\rangle \right) \nonumber \\&\qquad -\,N(N-1)\mathfrak {I}\left( \langle \!\langle \Psi _t, (V_{N}(x_1-x_2)-M_{\mu }(x_1-x_2))f_{\mu }(x_1-x_2){\widehat{r}} \Psi _t\rangle \!\rangle \right) \nonumber \\&\qquad -\,N(N-1)\mathfrak {I}\left( \langle \!\langle \Psi _t,g_{\mu }(x_{1}-x_{2}) {\widehat{r}}{\mathcal {Z}}^{\varphi _t}(x_1,x_2) \Psi _t\rangle \!\rangle \right) \nonumber \\&\qquad +\,N(N-1)\mathfrak {I}\left( \langle \!\langle \Psi _t,\left[ H_{V_N}, g_{\mu }(x_{1}-x_{2}) \right] {\widehat{r}} \Psi _t\rangle \!\rangle \right) . \end{aligned}$$

The first, second and the fourth line give $\gamma _b+\gamma _d+\gamma _e+ \gamma _f$. Using Definition (5.3) the commutator in the fifth line equals

$$\begin{aligned} {[}H_{V_N},g_{\mu }(x_1-x_2)]&=-[H_{V_N},f_{\mu }(x_1-x_2)]\\&=[\Delta _1+\Delta _2,f_{\mu }(x_1-x_2)] \\&=(\Delta _1+\Delta _2)f_{\mu }(x_1-x_2)\\&\quad +\,(2\nabla _1f_{\mu }(x_1-x_2))\nabla _1+(2\nabla _2f_{\mu }(x_1-x_2))\nabla _2 \\&=(V_{N}(x_1-x_2)-M_{\mu }(x_1-x_2))f_{\mu }(x_1-x_2)\\&\quad -\,(2\nabla _1g_{\mu }(x_1-x_2))\nabla _1-(2\nabla _2g_{\mu }(x_1-x_2))\nabla _2. \end{aligned}$$

Using symmetry the third and fifth line in (63) give

$$\begin{aligned} -4N(N-1)\langle \!\langle \Psi _t, (\nabla _1g_{\mu }(x_1-x_2))\nabla _1 {\widehat{r}}\Psi _t\rangle \!\rangle =\gamma _c (\Psi _t,\varphi _t). \end{aligned}$$

By means of

$$\begin{aligned} \frac{d}{dt}\left( {\mathcal {E}}_{M_\mu }(\Psi _t)-{\mathcal {E}}_{N \Vert M_\mu \Vert _1}^{GP}(\varphi _t)\right) = \gamma _a (\Psi _t, \varphi _t) \end{aligned}$$

and the fundamental theorem of calculus the result follows. $\quad \square$

#### The Grönwall estimate

Again, we will bound the time derivative of $\alpha (\Psi _t,\varphi _t)$ such that we can employ a Grönwall estimate.

##### Lemma 6.13

Let $V_N \in {\mathcal {V}}_N$. Let $\Psi _t$ the unique solution to $i \partial _t \Psi _t = H_{V_N} \Psi _t$ with initial datum $\Psi _0 \in L^2_{s}({\mathbb {R}}^{2N}, {\mathbb {C}}) \cap H^2({\mathbb {R}}^{2N}, {\mathbb {C}})$ and $\Vert \Psi _0\Vert =1$. Let $\varphi _t$ the unique solution to $i \partial _t \varphi _t = h^{GP}_{4 \pi } \varphi _t$ with $\varphi _t \in H^{3}({\mathbb {R}}^2,{\mathbb {C}})$ and $\Vert \varphi _0\Vert =1$. Let ${\mathcal {E}}_{V_N}(\Psi _0) \le C$. Let $\alpha (\Psi _t,\varphi _t)$, $\gamma _{i}(\Psi _t,\varphi _t), i \in \{ a, b, c, d, e, f\}$ be defined as in Definitions 6.8 and 6.11 with $\xi = 1/10$ and $\mu = 10$. Then,

64 $$\begin{aligned} \sum _{i \in \{ a, b, c, d, e, f\}} |\gamma _i(\Psi _t,\varphi _t) |&\le {\mathcal {K}}(\varphi _t, A_t) \Big ( \alpha (\Psi _t, \varphi _t) + N^{-1/10} \Big ). \end{aligned}$$

The proof of the Lemma can be found in Sect. 7.4. By means of Lemma 5.5 (h) and (i), the terms $\gamma _a$ and $\gamma _b$ can be estimated in the same way as $\gamma _a^<$ and $\gamma _b^<$. The estimates for $\gamma _c$, $\gamma _d$, $\gamma _e$ and $\gamma _f$ are based on the smallness of the $L^p$-norms of $g_{\mu }$, see Lemma 5.5 (i).

Thus, combining Lemmas 6.12 and 6.13, we obtain the following estimate for $\alpha (\Psi _t,\varphi _t)$ by means of Grönwall’s Lemma

##### Lemma 6.14

Let $V_N \in {\mathcal {V}}_N$. Let $\Psi _t$ the unique solution to $i \partial _t \Psi _t = H_{V_N} \Psi _t$ with initial datum $\Psi _0 \in L^2_{s}({\mathbb {R}}^{2N}, {\mathbb {C}}) \cap H^2({\mathbb {R}}^{2N}, {\mathbb {C}})$ and $\Vert \Psi _0\Vert =1$. Let $\varphi _t$ the unique solution to $i \partial _t \varphi _t = h^{GP}_{4 \pi } \varphi _t$ with $\varphi _t \in H^{3}({\mathbb {R}}^2,{\mathbb {C}})$. Let ${\mathcal {E}}_{V_N}(\Psi _0) \le C$. Let $\alpha (\Psi _t,\varphi _t)$ be defined as in Definition 6.8 with $\xi = 1/10$ and $\mu = 10$. Then,

65 $$\begin{aligned} \alpha (\Psi _t,\varphi _t)&\le e^{\int _0^t ds \, {\mathcal {K}}(\varphi _s, A_s) } \big ( \alpha (\Psi _0,\varphi _0) + N^{-1/10} \big ). \end{aligned}$$

##### Proof

This is proven in the same way as Lemma 6.7. $\quad \square$

##### Proof of Theorem 2.4: Part (b)

Again, we note that under the assumptions $\varphi _t \in H^{3}({\mathbb {R}}^2,{\mathbb {C}})$ and $A_{\cdot } \in C^1({\mathbb {R}},L^{\infty }({\mathbb {R}}^2,{\mathbb {R}}))$ there exists a constant $C_t < \infty$, depending on *t*, $\varphi _0$ and $A_t$, such that $\int _0^t ds {\mathcal {K}}(\varphi _s, A_s) \le C_t$, see Sect. 4.

Let $\xi = 1/10$ and $\mu = 10$. If we then combine Lemmas 6.10 and 6.14 to estimate

$$\begin{aligned} \text {Tr} \left| \gamma ^{(1)}_{\Psi _t} - |\varphi _t \rangle \langle \varphi _t| \right|&\le C \sqrt{\alpha (\Psi _t,\varphi _t)} + C \Vert \varphi _t \Vert _{\infty } N^{-1} \le e^{C_t} \big ( \sqrt{\alpha (\Psi _0,\varphi _0)} + N^{-1/20} \big ) \\&\le e^{C_t} \Bigg ( \root 4 \of {\text {Tr} \left| \gamma ^{(1)}_{\Psi _0} - |\varphi _0 \rangle \langle \varphi _0| \right| } + \sqrt{\left| {\mathcal {E}}_{V_N} (\Psi _0) - {\mathcal {E}}^{GP}_{4 \pi } (\varphi _0) \right| } + N^{-1/20} \Bigg ). \end{aligned}$$

Moreover, one obtains

$$\begin{aligned} \left| {\mathcal {E}}_{V_N} (\Psi _t) - {\mathcal {E}}^{GP}_{4 \pi } (\varphi _t) \right|&\le \alpha (\Psi _t,\varphi _t) + C \Vert \varphi _t \Vert _{\infty } N^{-1} \\&\le e^{C_t} \Bigg ( \sqrt{\text {Tr} \left| \gamma ^{(1)}_{\Psi _0} - |\varphi _0 \rangle \langle \varphi _0| \right| } + \left| {\mathcal {E}}_{V_N} (\Psi _0) - {\mathcal {E}}^{GP}_{4 \pi } (\varphi _0) \right| + N^{-1/10} \Bigg ). \end{aligned}$$

Finally, this shows part (b) of Theorem 2.4.$\quad \square$

## Rigorous Estimates

### Smearing out the potential $W_\beta$

To control the potential $W_\beta$ for $\beta$ large, we use a technique which allows us to replace the potential $W_\beta$ by some potential $U_{\beta _1, \beta } \in {\mathcal {W}}_{\beta _1}, \beta _1 < \beta$ with $\Vert W_\beta \Vert _1 =\Vert U_{\beta _1,\beta } \Vert _1$. For this, define $h_{\beta _1,\beta }$ by $\Delta h_{\beta _1,\beta }=W_{\beta }-U_{\beta _1,\beta }$. The function $h_{\beta _1,\beta }$ can be thought as an electrostatic potential which is caused by the charge $W_{\beta }-U_{\beta _1,\beta }$. It is then possible to rewrite

$$\begin{aligned}&\langle \!\langle \chi , W_\beta (x_1-x_2) \Omega \rangle \!\rangle = \langle \!\langle \chi , U_{\beta _1,\beta }(x_1-x_2) \Omega \rangle \!\rangle \\&\quad -\,\langle \!\langle \nabla _1 \chi , (\nabla _1 h_{\beta _1,\beta }) (x_1-x_2) \Omega \rangle \!\rangle - \langle \!\langle \chi , (\nabla _1 h_{\beta _1,\beta }) (x_1-x_2) \nabla _1 \Omega \rangle \!\rangle , \end{aligned}$$

for $\chi , \omega \in L^2_s({\mathbb {R}}^{2N},{\mathbb {C}})$. We will verify that the $L^p$-norms of $h_{\beta _1,\beta }$ and $\nabla h_{\beta _1,\beta }$ are better to control than the respective $L^p$-norm of $W_\beta$. With additional control of $\nabla _1 \Omega$ and $\nabla _1 \chi$, it is therefore possible to obtain a sufficient bound for $\langle \!\langle \chi , W_\beta (x_1-x_2) \Omega \rangle \!\rangle$ for large $\beta$.

#### Definition 7.1

For any $0\le \beta _1< \beta$ and any $W_{\beta }\in {\mathcal {W}}_\beta$ we define

$$\begin{aligned} U_{\beta _1,\beta }(x)=\left\{ \begin{array}{ll} \frac{4}{\pi }\Vert W_{\beta }\Vert _1N^{2\beta _1} &{}\quad \hbox {for }\ |x|<1/2 N^{-\beta _1}, \\ 0 &{}\quad \hbox {else.} \end{array} \right. \end{aligned}$$

and

66 $$\begin{aligned} h_{\beta _1,\beta }(x)= \frac{1}{2\pi } \int _{{\mathbb {R}}^2} \ln |x-y| (W_{\beta }(y)-U_{\beta _1,\beta }(y))d^2y. \end{aligned}$$

#### Lemma 7.2

Let $0\le \beta _1< \beta$, $W_{\beta }\in {\mathcal {W}}_\beta$ and $N \in {\mathbb {N}}$ large enough such that $\text {supp}(W_{\beta }) \subseteq \text {supp}(U_{\beta _1,\beta })$. Then,

- $$\begin{aligned}&U_{\beta _1,\beta } \in {\mathcal {W}}_{\beta _1}, \\&\Delta h_{\beta _1,\beta }=W_{\beta }-U_{\beta _1,\beta }. \end{aligned}$$
- Pointwise estimates
  67 $$\begin{aligned} h_{\beta _1,\beta }(x)&= 0 \text { for } |x| \ge 1/2 \, N^{- \beta _1}, \qquad |h_{\beta _1,\beta }(x)| \le CN ^{-1} \ln (N), \end{aligned}$$
  68 $$\begin{aligned} |\nabla h_{\beta _1,\beta }(x)|&\le CN ^{-1}\left( \vert x\vert ^2+N^{-2\beta }\right) ^{-\frac{1}{2}}. \end{aligned}$$
- Norm estimates
  $$\begin{aligned} \Vert h_{\beta _1,\beta }\Vert _\infty&\le CN ^{-1} \ln (N),\\ \qquad \Vert h_{\beta _1,\beta }\Vert _{\lambda }&\le CN ^{-1-\frac{2}{\lambda }\beta _1} \ln (N)\;\text { for }1\le \lambda \le \infty , \\ \Vert \nabla h_{\beta _1,\beta }\Vert _{\lambda }&\le CN ^{-1+\beta -\frac{2}{\lambda }\beta _1} \quad \text { for }1\le \lambda \le \infty . \end{aligned}$$
  Furthermore, for $\lambda =2$, we obtain the improved bounds
  69 $$\begin{aligned} \Vert h_{0,\beta }\Vert&\le CN ^{-1}, \end{aligned}$$
  70 $$\begin{aligned} \Vert \nabla h_{\beta _1,\beta }\Vert&\le CN ^{-1} (\ln (N))^{1/2}. \end{aligned}$$

#### Proof

- $U_{\beta _1,\beta } \in \widetilde{{\mathcal {W}}}_{\beta _1}$ follows directly from the definition of $U_{\beta _1,\beta }$. Since $W_{\beta } \in {\mathcal {W}}_{\beta }$ one has $\big | N ||U_{\beta _1,\beta } ||_1 - b_{W_{\beta }} \big | \le CN ^{-1} \ln (N)$ and consequently $U_{\beta _1,\beta } \in {\mathcal {W}}_{\beta _1}$. Furthermore, $h_{\beta _1,\beta }$ is a solution of Poisson’s equation because $-\frac{1}{2\pi } \ln \vert x-y \vert$ is the radially symmetric Green’s function of the Laplacian in two dimensions [36, Theorem 6.21].
- The first statement is a well known result from standard electrodynamics. It follows from Newton’s theorem [36, Theorem 9.7] and $\Vert U_{\beta _1,\beta } \Vert _{1} = \Vert W_{\beta } \Vert _{1}$. Heuristically speaking, $W_{\beta }$ can be understood as a charge density and $- U_{\beta _1,\beta }$ as a smeared out charge density of opposite sign such that the "total charge" is zero. Moreover if we use that $W_{\beta }(x) = U_{\beta _1,\beta }(x) =0$ for all $|x| \ge 1/2 N^{-\beta _1}$, we obtain the pointwise estimate
  $$\begin{aligned} \vert h_{\beta _1,\beta }(x)|&\le \frac{1}{2 \pi } \int _{B_{1/2 N^{-\beta _1}(0)}} d^2y \left| \ln |x-y| W_\beta (y) \right| \\&\quad +\, \frac{1}{2 \pi } \int _{B_{1/2 N^{-\beta _1}(0)}} d^2y \left| \ln |x-y| U_{\beta _1, \beta } (y) \right| . \end{aligned}$$
  Subsequently, we estimate each term separately. Therefore, it is useful to recall that there exists an $R \in (0,\infty )$ such that $W_{\beta }(x) = 0$ for all $\vert x \vert \ge R N^{-\beta }$. This allows us to bound the first summand by
  $$\begin{aligned} \int _{B_{1/2 N^{-\beta _1}(0)}} d^2y |\ln |x-y| | W_{\beta }(y)&\le \int _{B_{R N^{-\beta }(0)}} d^2y |\ln |x-y| | W_{\beta }(y). \end{aligned}$$
  For $2R N^{-\beta }< \vert x \vert < 1/2 N^{-\beta _1}$ one has $\vert x-y \vert \le N^{-\beta _1} \le 1$ in the integral above. This implies $|\ln |x-y||= - \ln |x-y|$ and leads to
  $$\begin{aligned} \int _{B_{1/2 N^{-\beta _1}(0)}} d^2y |\ln |x-y| | W_{\beta }(y)&\le - \Vert W_\beta \Vert _1 \ln (\vert x \vert - R N^{-\beta } ) \le - \Vert W_\beta \Vert _1 \ln ( R N^{-\beta } ) \\&\le C \Vert W_\beta \Vert _1 \ln N^{\beta }\le CN ^{-1} \ln \left( N \right) \end{aligned}$$
  for all $2R N^{-\beta }< \vert x \vert < 1/2 N^{-\beta _1}$. Let next $|x|\le 2 RN ^{-\beta }$. We again have $|x-y| \le 1$ for all $y \in B_{1/2N^{-\beta _1}(0)}$ and obtain
  $$\begin{aligned} \int _{B_{1/2N^{-\beta _1}(0)}} |\ln |x-y| | W_{\beta }(y)d^2y&\le C \Vert W_{\beta } \Vert _{\infty } \int _{ B_{ RN ^{-\beta }(0)}} - \ln \vert x-y \vert d^2y \\&\le CN ^{-1 + 2 \beta } \int _{ B_{ RN ^{-\beta }(x)}} - \ln \vert y\vert d^2y \\&\le CN ^{-1 + 2 \beta } \int _{B_{4 RN ^{-\beta }(0)}} - \ln \vert y\vert d^2y \\&= CN ^{-1 + 2 \beta }\Big [- \vert y\vert ^2 (2\ln \vert y \vert -1) \Big ]_0^{4 RN ^{-\beta }} \\&\le CN ^{-1} \ln \left( N^{\beta } \right) \end{aligned}$$
  for all $|x|\le 2 RN ^{-\beta }$. If we repeat the same estimate for $\vert x \vert \le 1/2 N^{-\beta _1}$ and $U_{\beta _1, \beta }$ with $\Vert U_{\beta _1, \beta } \Vert _{\infty } \le CN ^{-1+ 2 \beta _1}$ we get
  $$\begin{aligned} \int _{B_{1/2N^{-\beta _1}(0)}} |\ln |x-y| | U_{\beta _1, \beta }(y)d^2y&\le C \Vert U_{\beta _1, \beta } \Vert _{\infty } \int _{B_{1/2N^{-\beta _1}(0)}} - \ln \vert x-y \vert d^2y \\&\le CN ^{-1} \ln \left( N^{\beta _1} \right) , \end{aligned}$$
  which proves the first statement. For the gradient, we estimate the two terms on the r.h.s. of
  $$\begin{aligned} |\nabla h_{\beta _1,\beta }(x)|\le \frac{1}{2\pi }\int \frac{1}{|x-y|} W_{\beta }(y)d^2y + \frac{1}{2\pi }\int \frac{1}{|x-y|} U_{\beta _1,\beta }(y)d^2y \end{aligned}$$
  separately. Let first $2R N^{-\beta } \le \vert x \vert$. Similarly as in the previous argument, one finds
  $$\begin{aligned} \int \frac{1}{|x-y|} W_{\beta }(y)d^2y&\le \int _{B_{R N^{-\beta }}(0)} \frac{1}{\vert x-y\vert } W_{\beta }(y)d^2y \le \frac{\Vert W_\beta \Vert _1}{\vert x \vert - RN ^{-\beta }} \end{aligned}$$
  for $R N^{-\beta } \le \vert x \vert$, which implies that
  $$\begin{aligned} \int \frac{1}{|x-y|} W_{\beta }(y)d^2y \le \frac{C \Vert W_{\beta }\Vert _1}{( \vert x \vert ^2 + N^{-2\beta })^{\frac{1}{2}}} \le \frac{ CN ^{-1}}{( \vert x \vert ^2 + N^{-2\beta })^{\frac{1}{2}}} \end{aligned}$$
  for all $2R N^{-\beta } \le \vert x \vert$. For $\vert x \vert \le 2R N^{-\beta }$, we make use of
  $$\begin{aligned} N^{\beta } \le \frac{C}{\left( \vert x \vert ^2 + N^{- 2 \beta } \right) ^{1/2}} \end{aligned}$$
  and estimate
  $$\begin{aligned} \int \frac{1}{|x-y|} W_{\beta }(y) d^2y&\le \Vert W_{\beta } \Vert _{\infty } \int _{ B_{ RN ^{-\beta }(0)}} \frac{1}{\vert x-y \vert } d^2y \\&\le CN ^{2\beta -1}\int _{0}^{ RN ^{-\beta }} d\vert y\vert = C N^{-1+\beta } \le \frac{ CN ^{-1}}{\left( \vert x \vert ^2 + N^{- 2 \beta } \right) ^{1/2}}. \end{aligned}$$
  Equivalently, we obtain
  $$\begin{aligned} \int \frac{1}{|x-y|} U_{\beta _1,\beta }(y) d^2y&\le \Vert U_{\beta _1, \beta } \Vert _{\infty } \int _{ B_{N^{-\beta _1}(0)}} \frac{1}{\vert x-y \vert } d^2y \\&= CN ^{-1+\beta _1} \le \frac{ CN ^{-1}}{\left( \vert x \vert ^2 + N^{- 2 \beta _1} \right) ^{1/2}} \le \frac{ CN ^{-1}}{( \vert x \vert ^2 + N^{-2\beta })^{\frac{1}{2}}}, \end{aligned}$$
  for $\vert x \vert \le N^{-\beta _1}$. Since $\nabla h_{\beta _1,\beta }(x) = 0$ for $\vert x \vert \ge N^{-\beta _1}$, the second statement of (b) follows.
- The first part of (c) follows from (b) and the fact that the support of $h_{\beta 1,\beta }$ and $\nabla h_{\beta _1,\beta }$ has radius $\le CN ^{-\beta _1}$. The bounds on the $L^2$-norm can be improved by
  $$\begin{aligned} \Vert \nabla h_{\beta _1,\beta }\Vert _{2}^2&\le C \int _0^{ CN ^{-\beta _1}} dr r |\nabla h_{\beta _1,\beta }(r)|^2 \le \frac{C}{N^2} \int _0^{C N^{-\beta _1}} dr \frac{r}{r^2+N^{-2\beta } } \\&= \frac{C}{N^2} \ln \left( \frac{N^{-2\beta _1 }+N^{-2\beta }}{N^{-2\beta }} \right) \le \frac{C}{N^ 2} \ln (N). \end{aligned}$$
  By means of [36, Theorem 9.7] we obtain
  $$\begin{aligned} | h_{0,\beta }(x) |&\le \frac{1}{2 \pi } |\ln (x) | \int \left( U_{0,\beta }(y) + W_{\beta }(y) \right) d^2y \le CN ^{-1} |\ln (x) | \end{aligned}$$
  and
  $$\begin{aligned} \Vert h_{0,\beta }\Vert _2^2 \le CN ^{-2} \int _0^1 dr r \ln ^2(r) \le C N^{-2}, \end{aligned}$$
  where we have used that $h_{0,\beta }(x) = 0$ for all $|x| \ge 1$. $\square$

### Estimates on the cutoff

In order to smear out singular potentials as explained in the previous section and to obtain sufficient bounds, it seems at first necessary to show that $\Vert \nabla _1 q_1\Psi _t\Vert$ decays in *N*. However, this term will in fact not be small for the dynamic generated by $V_N$. There, we rather expect that $\Vert \nabla _1q_1\Psi _t\Vert = {\mathcal {O}}(1)$ holds. It has been shown in [18, 37] that the interaction energy is purely kinetic in the Gross–Pitaevskii regime, which implies that a relevant part of the kinetic energy is concentrated around the scattering centers. We must thus cutoff the part which is used to form the microscopic structure. For this, we define the set $\overline{{\mathcal {A}}}^{(d)}_j$ which includes all configurations where the distance between particle $x_i$ and particle $x_j,j \ne i$ is smaller than $N^ {-d}$. It is then possible to prove that the kinetic energy concentrated on the complement of $\overline{{\mathcal {A}}}_j^{(d)}$, i.e. $\Vert \mathbb {1}_{{\mathcal {A}}^{(d)}_1}\nabla _1q_1\Psi \Vert$, is small, see Lemma 7.9.

#### Definition 7.3

For any $$j,k=1, \dots ,N$$ and $$ d>0$$ let

71 $$\begin{aligned} a^{(d)}_{j,k}= & {} \{(x_1,x_2,\ldots ,x_N)\in {\mathbb {R}}^{2N}: |x_j-x_k|<N^{-d}\} \subseteq {\mathbb {R}}^{2N}\nonumber \\ \overline{{\mathcal {A}}}^{(d)}_j= & {} \bigcup _{k\ne j}a^{(d)}_{j,k}\;\;\;\;\;\;\;{\mathcal {A}}^{(d)}_j={\mathbb {R}}^{2N}\backslash \overline{{\mathcal {A}}}_j^{(d)} \;\;\;\;\;\;\;\overline{{\mathcal {B}}}^{(d)}_{j}=\bigcup _{k\ne l\ne j}a^{(d)}_{k,l}\;\;\;\;\;\;\;{\mathcal {B}}^{(d)}_{j}={\mathbb {R}}^{2N}\backslash \overline{{\mathcal {B}}}^{(d)}_{j}.\nonumber \\ \end{aligned}$$

#### Lemma 7.4

- For all $j\ne k$ with $1 \le j,k \le N$,
  $$\begin{aligned} \Vert \mathbb {1}_{\overline{{\mathcal {A}}}^{(d)}_{j}}p_j\Vert _{\text {op}}&\le C \Vert \varphi \Vert _{\infty }N^{1/2-d}, \\ \Vert \mathbb {1}_{\overline{{\mathcal {A}}}^{(d)}_{j}} \nabla _j p_j\Vert _{\text {op}}&\le C\Vert \nabla \varphi \Vert _{\infty } N^{1/2-d}, \\ \Vert \mathbb {1}_{a^{(d)}_{j,k}}p_j\Vert _{\text {op}}&\le C \Vert \varphi \Vert _\infty N^{-d}. \end{aligned}$$
- Let $\Psi \in H^1({\mathbb {R}}^{2N}, {\mathbb {C}})$. For any $1<p< \infty$, there exists a positive constant $C_p$, such that
  $$\begin{aligned} \Vert \mathbb {1}_{\overline{{\mathcal {A}}}^{(d)}_{1}}\Psi \Vert ^2&\le C_p N^{(1-2d)\frac{p-1}{p}} \Vert \nabla _1 \Psi \Vert ^{2\frac{p-1}{p}} \Vert \Psi \Vert ^{\frac{2}{p}}, \end{aligned}$$
- Let $\Psi \in L^2_{s}({\mathbb {R}}^{2N}, {\mathbb {C}}) \cap H^1({\mathbb {R}}^{2N}, {\mathbb {C}}), \Vert \Psi \Vert _{H^1} \le C$. For any $\epsilon > 0$, there exists a positive constant $C_\epsilon$ such that
  $$\begin{aligned} \Vert \mathbb {1}_{\overline{{\mathcal {B}}}^{(d)}_{j}}\Psi \Vert&\le C_\epsilon N^{1-d+ \epsilon }. \end{aligned}$$
- For any $$k\ne j$$
  $$\begin{aligned} \Vert [\mathbb {1}_{\overline{{\mathcal {A}}}^{(d)}_{j}},p_k]\Vert _{\text {op}}=\Vert [\mathbb {1}_{a^{(d)}_{j,k}},p_k]\Vert _{\text {op}}=\Vert [\mathbb {1}_{{\mathcal {A}}^{(d)}_{j}},p_k]\Vert _{\text {op}}\le C \Vert \varphi \Vert _\infty N^{-d}. \end{aligned}$$

#### Proof

- First note that the volume of the sets $a^{(d)}_{j,k}$ introduced in Definition 7.3 are $|a^{(d)}_{j,k}|=\pi N^{-2d}$.
  $$\begin{aligned} \Vert \mathbb {1}_{\overline{{\mathcal {A}}}^{(d)}_{j}}p_j\Vert _{\text {op}}&= \Vert \mathbb {1}_{\overline{{\mathcal {A}}}^{(d)}_{1}}p_1\Vert _{\text {op}}= \Vert p_1\mathbb {1}_{\overline{{\mathcal {A}}}^{(d)}_{1}}p_1\Vert _{\text {op}}^{\frac{1}{2}} \le \left( \Vert \varphi \Vert _\infty ^2\Vert \mathbb {1}_{\overline{{\mathcal {A}}}^{(d)}_{1}}\Vert _{1,\infty }\right) ^{1/2} \end{aligned}$$
  where we defined
  $$\begin{aligned} \Vert f \Vert _{p, \infty } = \sup _{x_2, \dots , x_N \in {\mathbb {R}}^2} \left( \int dx_1 |f(x_1, \dots , x_N)|^{p} \right) ^{\frac{1}{p}}. \end{aligned}$$
  Using $\mathbb {1}_{\overline{{\mathcal {A}}}^{(d)}_{1}} \le \sum _{k=2}^N \mathbb {1}_{a_{1,k}^{(d)}}$ as well as $\left( \mathbb {1}_{\overline{{\mathcal {A}}}^{(d)}_{1}} \right) ^p =\mathbb {1}_{\overline{{\mathcal {A}}}^{(d)}_{1}}$, we obtain
  $$\begin{aligned} \Vert \mathbb {1}_{\overline{{\mathcal {A}}}^{(d)}_{1}} \Vert _{p, \infty }&\le \sup _{x_2, \dots , x_N \in {\mathbb {R}}^2} \left( \int dx_1 \sum _{k=2}^N \mathbb {1}_{a_{1,k}^{(d)}} \right) ^{\frac{1}{p}} \le \left( N |a_{1,k}| \right) ^{\frac{1}{p}} \le CN ^{(1-2d) \frac{1}{p}}. \end{aligned}$$
  This implies
  $$\begin{aligned} \Vert \mathbb {1}_{\overline{{\mathcal {A}}}^{(d)}_{j}}p_j\Vert _{\text {op}}&\le C \Vert \varphi \Vert _\infty N^{\frac{1}{2}-d}. \end{aligned}$$
  The second statement of (a) can be proven similarly. Analogously, we obtain
  $$\begin{aligned} \Vert \mathbb {1}_{a^{(d)}_{j,k}}p_j\Vert _{\text {op}}&\le \Vert \varphi \Vert _\infty |a^{(d)}_{j,k}|^{1/2} \le C \Vert \varphi \Vert _\infty N^{-d}. \end{aligned}$$
- Without loss of generality, we can set $j=1$. Recall the two-dimensional Sobolev inequality, for $\varrho \in H^1({\mathbb {R}}^2,{\mathbb {C}})$ and for any $2<m<\infty$, there exists a positive constant $C_m$, such that $\Vert \varrho \Vert _m \le C_m \Vert \nabla \varrho \Vert ^{\frac{m-2}{m}} \Vert \varrho \Vert ^{\frac{2}{m}}$ holds. Using Hölder and Sobolev for the $x_1$-integration, we get, for $p>1$
  $$\begin{aligned} \Vert \mathbb {1}_{\overline{{\mathcal {A}}}^{(d)}_{1}}\Psi \Vert ^2&= \langle \!\langle \Psi ,\mathbb {1}_{\overline{{\mathcal {A}}}^{(d)}_{1}}\Psi \rangle \!\rangle \\&= \int d^2 x_2\dots d^2x_N \int d^2x_1 |\Psi (x_1, \dots ,x_N)|^2\mathbb {1}_{\overline{{\mathcal {A}}}^{(d)}_{1}}(x_1,\dots ,x_N) \\&\le \Vert \mathbb {1}_{\overline{{\mathcal {A}}}^{(d)}_{1}} \Vert _{\frac{p}{p-1}, \infty } \int d^2 x_2\dots d^2x_N \left( \int d^2x_1 |\Psi (x_1, \dots ,x_N)|^{2p} \right) ^{1/p} \\&\le C_p N^{(1-2d)\frac{p-1}{p}} \int d^2 x_2\dots d^2x_N \left( \int d^2x_1 |\nabla _1 \Psi (x_1, \dots ,x_N)|^{2} \right) ^{\frac{p-1}{p}} \\&\quad \times \,\left( \int d^2 {\tilde{x}}_1 | \Psi ({\tilde{x}}_1, \dots ,x_N)|^{2} \right) ^{\frac{1}{p}}, \end{aligned}$$
  where $C_p$ denotes a positive constant, depending on *p*. Using Hölder for the $x_2,\dots x_N$-integration with the conjugate pair $r= \frac{p}{p-1}$ and $s =p$, we then obtain
  $$\begin{aligned} \Vert \mathbb {1}_{\overline{{\mathcal {A}}}^{(d)}_{1}}\Psi \Vert ^2&\le C_p N^{(1-2d)\frac{p-1}{p}} \Vert \nabla _1 \Psi \Vert ^{2\frac{p-1}{p}} \Vert \Psi \Vert ^{\frac{2}{p}}. \end{aligned}$$
- We use that $\overline{{\mathcal {B}}}^{(d)}_{j} \subset \bigcup _{k=1}^N \overline{{\mathcal {A}}}^{(d)}_{k}$. Hence one can find pairwise disjoint sets ${\mathcal {C}}_k\subset \overline{{\mathcal {A}}}^{(d)}_{k}$, $k=1,\ldots ,N$ such that $\overline{{\mathcal {B}}}^{(d)}_{j}\subset \bigcup _{k=1}^N {\mathcal {C}}_{k}$. Since the sets ${\mathcal {C}}_k$ are pairwise disjoint, the $\mathbb {1}_{ {\mathcal {C}}_{k}}\Psi$ are pairwise orthogonal and we get
  $$\begin{aligned} \Vert \mathbb {1}_{\overline{{\mathcal {B}}}^{(d)}_{j}}\Psi \Vert ^2=\sum _{k=1}\Vert \mathbb {1}_{ {\mathcal {C}}_{k}}\Psi \Vert ^2 \le \sum _{k=1}^N\Vert \mathbb {1}_{\overline{{\mathcal {A}}}^{(d)}_{k}}\Psi \Vert ^2. \end{aligned}$$
- $$\begin{aligned} \Vert [\mathbb {1}_{\overline{{\mathcal {A}}}^{(d)}_{1}},p_2]\Vert _{\text {op}}&\le \Vert [\mathbb {1}_{a_{1,2}},p_2]\Vert _{\text {op}} \le \Vert \mathbb {1}_{a_{1,2}}p_2\Vert _{\text {op}}+\Vert p_2\mathbb {1}_{a_{1,2}}\Vert _{\text {op}} \\&\le 2\Vert \varphi \Vert _\infty |a_{1,2}|^{\frac{1}{2}}\le C \Vert \varphi \Vert _\infty N^{-d}. \end{aligned}$$

$$\square $$

### Proof of Lemma 6.6

The goal of this section is to prove Lemma 6.6. To this end, we bound each of the functionals $\gamma _a^<$, $\gamma _b^<$ and $\gamma _c^<$ separately and then collect the estimates. In view of the conditions required in Lemma 6.6, the following is assumed in the rest of this section:

* Let*$\beta >0$, $W_\beta \in {\mathcal {W}}_\beta$, $\varphi \in H^{3}({\mathbb {R}}^2,{\mathbb {C}})$*with*$\Vert \varphi \Vert =1$*and*$\Psi \in L^2_{s}({\mathbb {R}}^{2N}, {\mathbb {C}}) \cap H^2({\mathbb {R}}^{2N}, {\mathbb {C}})$*with*$\Vert \Psi \Vert =1$*such that*${\mathcal {E}}_{W_\beta }(\Psi ) \le C$.

**Control of** $$\gamma ^<_a$$

#### Lemma 7.5

For any function $B \in L^{\infty }({\mathbb {R}}^2, {\mathbb {R}})$, any $\varphi \in L^2({\mathbb {R}}^ 2, {\mathbb {C}})$ with $\Vert \varphi \Vert =1$ and any $\Psi \in L_s^2({\mathbb {R}}^{2N},{\mathbb {C}})$ with $\Vert \Psi \Vert =1$ we have

$$\begin{aligned} |\langle \!\langle \Psi , B(x_1) \Psi \rangle \!\rangle -\langle \varphi ,B\varphi \rangle |\le C \Vert B\Vert _\infty ( \langle \!\langle \Psi , {\widehat{n}}^\varphi \Psi \rangle \!\rangle + N^{-\frac{1}{2}}). \end{aligned}$$

#### Proof

Using $$1=p_1+q_1$$,

$$\begin{aligned}&\langle \!\langle \Psi , B(x_1) \Psi \rangle \!\rangle -\langle \varphi ,B\varphi \rangle \\&\quad = \langle \!\langle \Psi , p_1B (x_1) p_1\Psi \rangle \!\rangle +2\mathfrak {R}\langle \!\langle \Psi , q_1B(x_1) p_1\Psi \rangle \!\rangle +\langle \!\langle \Psi , q_1B (x_1) q_1\Psi \rangle \!\rangle -\langle \varphi , B\varphi \rangle \\&\quad \le \langle \varphi , B\varphi \rangle (\Vert p_1\Psi \Vert ^2-1)+2\mathfrak {R}\langle \!\langle \Psi , {\widehat{n}}^{-1/2}q_1B(x_1) p_1{\widehat{n}}_1^{1/2}\Psi \rangle \!\rangle + \langle \!\langle \Psi , q_1B(x_1) q_1\Psi \rangle \!\rangle , \end{aligned}$$

where we used Lemma 4.2 (c). Since $\Vert p_1\Psi \Vert ^2-1 = - \Vert q_1\Psi \Vert ^2$ it follows that

72 $$\begin{aligned} |\langle \!\langle \Psi , B(x_1) \Psi \rangle \!\rangle -\langle \varphi ,B\varphi \rangle |&\le C\Vert B\Vert _\infty \left( \langle \!\langle \Psi ,{\widehat{n}}^2\Psi \rangle \!\rangle + \langle \!\langle \Psi ,{\widehat{n}}_1\Psi \rangle \!\rangle + \langle \!\langle \Psi ,{\widehat{n}}\Psi \rangle \!\rangle \right) \nonumber \\&\le C \Vert B\Vert _\infty ( \langle \!\langle \Psi ,{\widehat{n}}\Psi \rangle \!\rangle + N^{-\frac{1}{2}}). \end{aligned}$$

$$\square $$

Using Lemma 7.5, $\Vert {\widehat{n}} - {\widehat{m}} \Vert _{op} \le CN ^{- \xi }$ and setting $B=\dot{A}_t$, we get

73 $$\begin{aligned} | \gamma _a^<(\Psi ,\varphi ) | \le C \Vert \dot{A}_t\Vert _\infty ( \langle \!\langle \Psi ,{\widehat{n}} \Psi \rangle \!\rangle + N^{-\frac{1}{2}} ) \le C \Vert \dot{A}_t\Vert _\infty ( \alpha ^<(\Psi ,\varphi ) + N^{- \xi } ). \end{aligned}$$

**Control of**$\gamma ^<_b$ To control $\gamma _b^<$ we will first prove that $\Vert \nabla _1 \Psi _t \Vert$ is uniformly bounded in *N*, if initially the energy per particle ${\mathcal {E}}_{U}(\Psi _0)$ is of order one.

#### Lemma 7.6

Let $\Psi _0 \in L^2_{s}({\mathbb {R}}^{2N}, {\mathbb {C}}) \cap H^2({\mathbb {R}}^{2N}, {\mathbb {C}})$ with $\Vert \Psi _0\Vert =1$. For any $U \in L^2({\mathbb {R}}^2,{\mathbb {R}}),\; U(x) \ge 0$, let $\Psi _t$ the unique solution to $i \partial _t \Psi _t = H_{U} \Psi _t$ with initial datum $\Psi _0$. Let ${\mathcal {E}}_{U}(\Psi _0) \le C$. Then

$$\begin{aligned} \Vert \nabla _1 \Psi _t \Vert \le {\mathcal {K}}(\varphi _t, A_t). \end{aligned}$$

#### Proof

Using $\frac{d}{dt} {\mathcal {E}}_{U}(\Psi _t) \le \Vert \dot{A}_t\Vert _\infty$, we obtain ${\mathcal {E}}_{U}(\Psi _t) \le {\mathcal {K}}(\varphi _t, A_t)$. This yields

$$\begin{aligned} \Vert \nabla _1 \Psi _t \Vert ^2 \le {\mathcal {K}}(\varphi _t, A_t)- \frac{N-1}{2} \Vert \sqrt{U}(x_1-x_2) \Psi _t \Vert ^2 + \Vert A_t \Vert _\infty \le {\mathcal {K}}(\varphi _t, A_t). \end{aligned}$$

$$\square $$

Next, we control ${\widehat{m}}^a$ and ${\widehat{m}}^b$ which were defined in Definition 6.4. The difference $m(k)-m(k+1)$ and $m(k)-m(k+2)$ is of leading order given by the derivative of the function *m*(*k*)—*k* understood as real variable—with respect to *k*. The *k*-derivative of *m*(*k*) equals

74 $$\begin{aligned} m(k)^\prime =\left\{ \begin{array}{ll} 1/(2\sqrt{kN}), &{}\quad \hbox {for}\,k\ge N^{1-2\xi }; \\ 1/2(N^{-1+\xi }), &{}\quad \hbox {else.} \end{array} \right. \end{aligned}$$

It is then easy to show that, for any $j\in {\mathbb {Z}}$, there exists a $C_j<\infty$ such that

75 $$\begin{aligned} {\widehat{m}}_j^x&\le C_jN^{-1} {\widehat{n}}^{-1}\text { for }x\in \{a,b\} \end{aligned}$$

76 $$\begin{aligned} \Vert {\widehat{m}}_j^x\Vert _{\text {op}}&\le C_jN^{-1+\xi } \text { for }x\in \{a,b\} \end{aligned}$$

77 $$\begin{aligned} \Vert {\widehat{n}}{\widehat{m}}_j^x\Vert _{\text {op}}&\le C_jN^{-1} \text { for }x\in \{a,b\} \end{aligned}$$

78 $$\begin{aligned} \Vert {\widehat{r}}\Vert _{\text {op}}&\le \Vert {\widehat{m}}^a\Vert _{\text {op}}+\Vert {\widehat{m}}^b\Vert _{\text {op}} \le CN ^{-1+\xi }. \end{aligned}$$

Now, we prove some general bounds, which will allow us to estimate the different terms of $\gamma ^<_b$ in (44). In order to facilitate the notation, let ${\widehat{w}} \in \lbrace N {\widehat{m}}^a_{-1},N {\widehat{m}}^b_{-2} \rbrace$. Then $w(k) < C n(k)^{-1}$ and $\Vert {\widehat{w}}_1 \Vert _{\text {op}} \le C \Vert {\widehat{w}}\Vert _{\text {op}}\le CN ^{\xi }$ follows.

#### Lemma 7.7

Let $\beta >0$ and $W_\beta \in {\mathcal {W}}_\beta$. Let $\Psi \in L^2_{s}({\mathbb {R}}^{2N}, {\mathbb {C}}) \cap H^2({\mathbb {R}}^{2N}, {\mathbb {C}})$ with $\Vert \Psi \Vert =1$ and let $\Vert \nabla _1\Psi \Vert \le {\mathcal {K}}(\varphi , A_t)$. Let $w(k)<n(k)^{-1}$ and $\Vert {\widehat{w}}_1 \Vert _{\text {op}} \le C \Vert {\widehat{w}}\Vert _{\text {op}} \le CN ^{ \xi }$ for some $0<\xi < 1/3$. Then,

- $$\begin{aligned} N\left| \langle \!\langle \Psi , p_1p_2 Z^\varphi _\beta (x_1,x_2) q_1p_2{\widehat{w}}\Psi \rangle \!\rangle \right| \le {\mathcal {K}}(\varphi , A_t) \left( N^{-1}+ N^{- 2 \beta } \ln (N) \right) . \end{aligned}$$
- $$\begin{aligned}&N \left| \langle \!\langle \Psi , p_1p_2 W_{\beta }(x_1-x_2) {\widehat{w}}q_1q_2\Psi \rangle \!\rangle \right| \le {\mathcal {K}}(\varphi ,A_t) \bigg ( \langle \!\langle \Psi , {\widehat{n}} \Psi \rangle \!\rangle \\&\quad +\,\inf _{ \min \{\beta , 1/2 \}>\beta _1>0} \, \inf _{\eta >0} \left( N^{ \eta - 2 \beta _1} \ln (N)^2 + \Vert {\widehat{w}}\Vert _{\text {op}} N^{-1+2 \beta _1} + \Vert {\widehat{w}}\Vert _{\text {op}}^2 N^{ - \eta } \right) \bigg ). \end{aligned}$$
  In addition, we have the slightly improved bound
  79 $$\begin{aligned} N|\langle \!\langle \Psi ,p_1 p_2 W_\beta (x_1-x_2) q_1 q_2 {\widehat{w}}\Psi \rangle \!\rangle |\le {\mathcal {K}}(\varphi , A_t) \left( \langle \!\langle \Psi ,{\widehat{n}}\Psi \rangle \!\rangle + \Vert {\widehat{w}}\Vert _{\text {op}} N^{-1+2 \beta }\right) \end{aligned}$$
  for all $$\beta < 1/2$$.
- $$\begin{aligned}&N|\langle \!\langle \Psi , p_1q_2Z^\varphi _\beta (x_1,x_2){\widehat{w}}q_1q_2\Psi \rangle \!\rangle | \le {\mathcal {K}}(\varphi , A_t) \bigg ( \langle \!\langle \Psi ,{\widehat{n}}\Psi \rangle \!\rangle + N^{-1/6} \ln (N) \\&\quad +\,\inf \left\{ \left| {\mathcal {E}}_{V_N}(\Psi )-{\mathcal {E}}_{4 \pi }^{GP}(\varphi )\right| , \left| {\mathcal {E}}_{W_\beta }(\Psi )- {\mathcal {E}}_{b_{W_{\beta }}}^{GP}(\varphi ) \right| + N^{-2 \beta } \ln (N) \right\} \bigg ). \end{aligned}$$

#### Proof

Since the left hand sides of all these statements are bounded, it follows that all these estimates hold uniformly in *N* being in any finite subset of ${\mathbb {N}}$. Hence it suffices to prove the validity of (a), (b) and (c) for sufficiently large $N\in {\mathbb {N}}$.

- In view of Lemma 4.4, we obtain
  $$\begin{aligned} N \left| \langle \!\langle \Psi , p_1p_2 Z^\varphi _\beta (x_1,x_2) q_1p_2{\widehat{w}}\Psi \rangle \!\rangle \right|&\le N\Vert p_1p_2 Z^\varphi _\beta (x_1,x_2) q_1p_2\Vert _{\text {op}}\Vert {\widehat{n}}{\widehat{w}}\Psi \Vert \\&\le CN\Vert p_1p_2 Z^\varphi _\beta (x_1,x_2) q_1p_2\Vert _{\text {op}}. \end{aligned}$$
  $\Vert p_1p_2 Z^\varphi _\beta (x_1,x_2) q_1p_2\Vert _{\text {op}}$ can be estimated using $p_1q_1=0$ and (19):
  $$\begin{aligned}&N \left\| p_1p_2 \left( W_\beta (x_1-x_2)-\frac{N\Vert W_\beta \Vert _1}{N-1}|\varphi (x_1)|^2-\frac{N\Vert W_\beta \Vert _1}{N-1}| \varphi (x_2)|^2 \right) q_1p_2 \right\| _{\mathrm{{op}}} \\&\quad \le \Vert p_1p_2 ({ NW }_\beta (x_1-x_2)-N\Vert W_\beta \Vert _1|\varphi (x_1)|^2) p_2\Vert _{\mathrm{{op}}} +C \Vert \varphi \Vert _\infty ^2 N^{-1} \\&\quad \le \Vert \varphi \Vert _{\infty } \ \Vert N(W_\beta *|\varphi |^2)-\Vert { NW }_\beta \Vert _1|\varphi |^2\Vert +C \Vert \varphi \Vert _\infty ^2 N^{-1}. \end{aligned}$$
  Let *h* be given by
  $$\begin{aligned} h(x)=- \frac{1}{2 \pi }\int _{{\mathbb {R}}^2}d^2y \ln |x-y| NW _\beta (y) + \frac{1}{2\pi }\Vert NW _\beta \Vert _1\ln |x|, \end{aligned}$$
  which implies
  $$\begin{aligned} \Delta h(x)=N W_\beta (x) - \Vert NW _\beta \Vert _1 \delta (x). \end{aligned}$$
  As above (see Lemma 7.2), we obtain $h(x)=0$ for $x \notin B_{ RN ^{- \beta }}(0)$, where $R N^{-\beta }$ is the radius of the support of $W_\beta$. Thus,
  80 $$\begin{aligned} \Vert h\Vert _1&\le \frac{1}{2 \pi } \int _{{\mathbb {R}}^2}d^2x \int _{{\mathbb {R}}^2} d^2y|\ln |x-y| \, | \mathbb {1}_{B_{ RN ^{- \beta }}(0)}(x) NW _\beta (y) \nonumber \\&\quad +\,\frac{1}{2 \pi } N\Vert W_\beta \Vert _1\int _{{\mathbb {R}}^2} d^2x | \ln |x| \, | \mathbb {1}_{B_{ RN ^{- \beta }}(0)}(x) \le CN ^{-2 \beta } \ln (N). \end{aligned}$$
  Integration by parts and Young’s inequality give that
  $$\begin{aligned}&\Vert N(W_\beta *|\varphi |^2)-\Vert { NW }_\beta \Vert _1|\varphi |^2\Vert =\Vert (\Delta h)*|\varphi |^2\Vert \\&\quad \le \Vert h\Vert _1 \Vert \Delta |\varphi |^2\Vert _2 \le {\mathcal {K}}(\varphi , A_t) N^{-2\beta } \ln (N). \end{aligned}$$
  Thus, we obtain the bound
  81 $$\begin{aligned} N \left| \langle \!\langle \Psi , p_1p_2 Z^\varphi _\beta (x_1,x_2) q_1p_2{\widehat{w}}\Psi \rangle \!\rangle \right| \le {\mathcal {K}}(\varphi , A_t) \left( N^{-1}+ N^{- 2 \beta } \ln (N) \right) , \end{aligned}$$
  which then proves (a).
- We will first consider $\beta <1/2$. Using Lemmas 4.2 (c) and 4.6 with $O_{1,2}=q_2 W_\beta (x_1-x_2) p_2$, $\Omega =N^{-1/2}({\widehat{w}})^{1/2}q_1\Psi$ and $\chi =N^{1/2}p_1({\widehat{w}}_{2})^{1/2}\Psi$ we get
  $$\begin{aligned}&|\langle \!\langle \Psi ,p_1 p_2 W_\beta (x_1-x_2) q_1 q_2 {\widehat{w}}\Psi \rangle \!\rangle | \\&\quad =|\langle \!\langle \Psi ,({\widehat{w}})^{1/2}q_1 q_2 W_\beta (x_1-x_2)p_1 p_2({\widehat{w}}_{2})^{1/2}\Psi \rangle \!\rangle | \\&\quad \le N^{-1}\left\| ({\widehat{w}})^{1/2}q_1\Psi \right\| ^2+N\big |\langle \!\langle q_2({\widehat{w}}_{2})^{1/2}\,\Psi ,p_1\sqrt{W_\beta }(x_1-x_2) p_3 \sqrt{W_\beta }(x_1-x_3)\\&\qquad \sqrt{W_\beta }(x_1-x_2)p_2\sqrt{W_\beta }(x_1-x_3)p_1 q_3({\widehat{w}}_{2})^{1/2}\,\Psi \rangle \!\rangle \big | \\&\qquad +\,N(N-1)^{-1}\Vert q_2 W_\beta (x_1-x_2) p_2p_1({\widehat{w}}_{2})^{1/2}\Psi \Vert ^2 \\&\quad \le N^{-1}\left\| ({\widehat{w}})^{1/2}q_1\Psi \right\| ^2+N\Vert \sqrt{W_\beta }(x_1-x_2)p_1\Vert _{\text {op}}^4\;\Vert q_2({\widehat{w}}_{2})^{1/2}\,\Psi \Vert ^2 \\&\qquad +\,2 N(N-1)^{-1}\Vert p_1q_2({\widehat{w}}_{1})^{1/2} W_\beta (x_1-x_2) p_2p_1\Psi \Vert ^2 \\&\qquad +\,2 N(N-1)^{-1}\Vert q_1q_2 ({\widehat{w}})^{1/2} W_\beta (x_1-x_2) p_2p_1\Psi \Vert ^2. \end{aligned}$$
  With Lemma 4.2 (e) we get the bound
  $$\begin{aligned}&\le N^{-1}\Vert ({\widehat{w}})^{1/2}{\widehat{n}}\Psi \Vert ^2+N\Vert \varphi \Vert _\infty ^4\Vert W_\beta \Vert _1^2\;\Vert {\widehat{n}}({\widehat{w}}_{2})^{1/2}\,\Psi \Vert ^2 \\&\quad +\,2 N(N-1)^{-1}\Vert W_\beta \Vert ^2\Vert \varphi \Vert _\infty ^2 \left( \Vert {\widehat{w}}_{1}\Vert _{\text {op}} + \Vert {\widehat{w}}\Vert _{\text {op}} \right) . \end{aligned}$$
  Note, that $\Vert W_\beta \Vert _1\le CN ^{-1}$, $\Vert W_\beta \Vert ^2\le CN ^{-2+2\beta }$. Furthermore, using ${\widehat{w}}_2 \le ({\widehat{n}})^{-1}$, we have under the conditions on ${\widehat{w}}$
  $$\begin{aligned} \Vert {\widehat{n}} ({\widehat{w}}_2)^{1/2} \Psi \Vert&\le \Vert {\widehat{n}} ({\widehat{n}})^{-1/2} \Psi \Vert = \Vert ({\widehat{n}})^{1/2} \Psi \Vert = \sqrt{\langle \Psi ,{\widehat{n}}\Psi \rangle }. \end{aligned}$$
  In total, we obtain
  $$\begin{aligned} N|\langle \!\langle \Psi ,p_1 p_2 W_\beta (x_1-x_2) q_1 q_2 {\widehat{w}}\Psi \rangle \!\rangle |\le {\mathcal {K}}(\varphi , A_t) \left( \langle \!\langle \Psi ,{\widehat{n}}\Psi \rangle \!\rangle + \Vert {\widehat{w}}\Vert _{\text {op}} N^{-1+2 \beta }\right) \end{aligned}$$
  and we get (b) for the case $\beta <1/2$.
- We prove part (b) for general $\beta >0$. We use $U_{\beta _1,\beta }$ from Definition 7.1 for some $0<\beta _1< \min \{ \beta , 1/2 \}$. We then obtain
  82 $$\begin{aligned}&N \langle \!\langle \Psi , p_1p_2 W_{\beta }(x_1-x_2) {\widehat{w}}q_1q_2\Psi \rangle \!\rangle \nonumber \\&\quad = N\langle \!\langle \Psi , p_1p_2 U_{\beta _1,\beta }(x_1-x_2) {\widehat{w}}q_1q_2\Psi \rangle \!\rangle \end{aligned}$$
  83 $$\begin{aligned}&\qquad +\, N\langle \!\langle \Psi , p_1p_2 \left( W_{\beta }(x_1-x_2)-U_{\beta _1,\beta }(x_1-x_2)\right) {\widehat{w}}q_1q_2\Psi \rangle \!\rangle . \end{aligned}$$
  Term (82) has been controlled above. So we are left to control (83). Let $\Delta h_{\beta _1,\beta }=W_{\beta }-U_{\beta _1,\beta }$. Integrating by parts and using that $\nabla _1 h_{\beta _1,\beta }(x_1-x_2)=-\nabla _2 h_{\beta _1,\beta }(x_1-x_2)$ gives
  84 $$\begin{aligned}&N \left| \langle \!\langle \Psi , p_1p_2\left( W_{\beta }(x_1-x_2)-U_{\beta _1,\beta }(x_1-x_2)\right) {\widehat{w}}q_1q_2\Psi \rangle \!\rangle \right| \nonumber \\&\quad \le N\left| \langle \!\langle \nabla _1p_1\Psi , p_2\nabla _2 h_{\beta _1,\beta }(x_1-x_2){\widehat{w}} q_1q_2\Psi \rangle \!\rangle \right| \end{aligned}$$
  85 $$\begin{aligned}&\qquad +\, N\left| \langle \!\langle \Psi , p_1p_2\nabla _2 h_{\beta _1,\beta }(x_1-x_2) \nabla _1{\widehat{w}}q_1q_2\Psi \rangle \!\rangle \right| . \end{aligned}$$
  Let $t_1 \in \lbrace p_1, \nabla _1 p_1 \rbrace$ and let $\Gamma \in \lbrace {\widehat{w}}q_1\Psi , \nabla _1 {\widehat{w}}q_1\Psi \rbrace$. For both (84) and (85), we use Lemma 4.6 with $O_{1,2}=N^{1+ \eta /2} q_2\nabla _2 h_{\beta _1,\beta }(x_1-x_2)p_2$, $\chi =t_1\Psi$ and $\Omega = N^{- \eta /2} \Gamma$. This yields
  86 $$\begin{aligned} {}(84)+(85)&\le 2 \sup _{t_1 \in \lbrace p_1, \nabla _1 p_1 \rbrace , \Gamma \in \lbrace {\widehat{w}}q_1\Psi , \nabla _1 {\widehat{w}}q_1\Psi \rbrace } \Big ( N^{-\eta } \Vert \Gamma \Vert ^2 \end{aligned}$$
  87 $$\begin{aligned}&\quad +\, \frac{N^{2+\eta }}{N-1}\Vert q_2\nabla _2 h_{\beta _1,\beta }(x_1-x_2)t_1p_2\Psi \Vert ^2 \end{aligned}$$
  88 $$\begin{aligned}&\quad +\, N^{2+\eta }\left| \langle \!\langle \Psi ,t_1p_2 q_3 \nabla _2 h_{\beta _1,\beta }(x_1-x_2)\nabla _3 h_{\beta _1,\beta } (x_1-x_3)t_1q_2 p_3\Psi \rangle \!\rangle \right| \Big ). \end{aligned}$$
  The first term can be bounded using Corollary 4.5 by
  $$\begin{aligned} N^{-\eta }\Vert \nabla _1 {\widehat{w}}q_1\Psi \Vert ^2&\le 4 N^{-\eta } \Vert {\widehat{w}} \Vert ^2_{\text {op}} \Vert \nabla _1 q_1 \Psi \Vert ^2 \\ N^{-\eta }\Vert {\widehat{w}}q_1\Psi \Vert ^2&\le CN ^{-\eta }. \end{aligned}$$
  Thus (86) $\le {\mathcal {K}}(\varphi , A_t) N^{-\eta } \Vert {\widehat{w}} \Vert ^2_{\text {op}}$ using that $\Vert \nabla _1 q_1 \Psi \Vert \le {\mathcal {K}}(\varphi , A_t)$. By $\Vert t_1\Psi \Vert ^2 \le {\mathcal {K}}(\varphi , A_t)$, we obtain
  $$\begin{aligned} (87)&\le {\mathcal {K}}(\varphi , A_t) \frac{N^{2+\eta }}{N-1}\Vert \nabla _2 h_{\beta _1,\beta }(x_1-x_2)p_2\Vert _{\text {op}}^2 \le {\mathcal {K}}(\varphi , A_t) \frac{N^{2+\eta }}{N-1} \Vert \varphi \Vert _\infty ^2 \Vert \nabla h_{\beta _1,\beta }\Vert ^2 \\&\le {\mathcal {K}}(\varphi , A_t) N^{\eta -1}\ln (N), \end{aligned}$$
  where we used Lemma 7.2 in the last step. Next, we estimate
  $$\begin{aligned} (88)&\le N^{2+\eta }\Vert p_2 \nabla _2 h_{\beta _1,\beta }(x_1-x_2)t_1 q_2 \Psi \Vert ^2\ \\&\le 2N^{2+\eta }\Vert p_2 h_{\beta _1,\beta }(x_1-x_2)t_1 \nabla _2q_2 \Psi \Vert ^2 \\&\quad +\, 2N^{2+\eta }\Vert |\varphi (x_2)\rangle \langle \nabla \varphi (x_2)| h_{\beta _1,\beta }(x_1-x_2)t_1 q_2 \Psi \Vert ^2\ \\&\le 2N^{2+\eta }\Vert p_2 h_{\beta _1,\beta }(x_1-x_2) \Vert _{\text {op}}^2 \Vert t_1 \nabla _2 q_2 \Psi \Vert ^2\ \\&\quad +\, 2N^{2+\eta } \Vert |\varphi (x_2)\rangle \langle \nabla \varphi (x_2)| h_{\beta _1,\beta }(x_1-x_2) \Vert _{\text {op}}^2 \Vert t_1 q_2 \Psi \Vert ^2\ \\&\le {\mathcal {K}}(\varphi , A_t) N^{2+\eta } \Vert h_{\beta _1,\beta } \Vert ^2 \\&\le {\mathcal {K}}(\varphi , A_t) N^{\eta - 2\beta _1} \ln (N)^2. \end{aligned}$$
  Thus, for all $\eta \in {\mathbb {R}}$
  $$\begin{aligned}&N\langle \!\langle \Psi , p_1p_2 \left( W_{\beta }(x_1-x_2)-U_{\beta _1,\beta }(x_1-x_2)\right) {\widehat{w}}q_1q_2\Psi \rangle \!\rangle \\&\quad \le {\mathcal {K}}(\varphi ,A_t) \left( \Vert {\widehat{w}}\Vert _{\text {op}}^2 N^{ - \eta } + N^{ \eta -1} \ln (N) +N^{ \eta - 2 \beta _1} \ln (N)^2 \right) . \end{aligned}$$
  Combining the estimates and using $N^{ \eta -1} \ln (N) < N^{ \eta - 2 \beta _1} \ln (N)^2$, we obtain
  $$\begin{aligned}&N\langle \!\langle \Psi , p_1p_2 W_{\beta }(x_1-x_2) {\widehat{w}}q_1q_2\Psi \rangle \!\rangle \le {\mathcal {K}}(\varphi ,A_t) \bigg ( \langle \!\langle \Psi , {\widehat{n}} \Psi \rangle \!\rangle \\&\quad +\,\inf _{ \min \{\beta , 1/2 \}>\beta _1>0} \, \inf _{\eta >0} \left( N^{ \eta - 2 \beta _1} \ln (N)^2 + \Vert {\widehat{w}}\Vert _{\text {op}} N^{-1+2 \beta _1} + \Vert {\widehat{w}}\Vert _{\text {op}}^2 N^{ - \eta } \right) \bigg ). \end{aligned}$$
- We note that $q_1 p_2 |\varphi |^2(x_1) q_1q_2=0$ and estimate
  $$\begin{aligned}&N \left| \langle \!\langle \Psi , q_1p_2 \frac{N \Vert W_\beta \Vert _1}{N-1} |\varphi |^2(x_2) {\widehat{w}}q_1q_2\Psi \rangle \!\rangle \right| \le C \Vert \varphi \Vert _\infty ^2 \Vert {\widehat{w}} \, {\widehat{n}} \Vert _{\text {op}} \Vert q_1 \Psi \Vert ^2 \\&\quad \le {\mathcal {K}}(\varphi , A_t) \langle \!\langle \Psi , {\widehat{n}} \Psi \rangle \!\rangle . \end{aligned}$$
  Hence, it is left to estimate $N \left| \langle \!\langle \Psi , q_1p_2W_\beta (x_1-x_2){\widehat{w}}q_1q_2\Psi \rangle \!\rangle \right|$. Let $U_{0,\beta }$ be given as in Definition 7.1. Moreover, let ${\mathcal {A}}^{(d)}_{1}$ and $\overline{{\mathcal {A}}}^{(d)}_{1}$ be defined as in Definition 7.3 with $d \ge \max \{7, 3 + \beta \}$. We use Lemma 4.2 (c) and integrating by parts to get
  89 $$\begin{aligned}&N\left| \langle \!\langle \Psi , q_1p_2W_\beta (x_1-x_2){\widehat{w}}q_1q_2\Psi \rangle \!\rangle \right| \nonumber \\&\quad \le N\left| \langle \!\langle \Psi ,q_1p_2U_{0,\beta }(x_1-x_2)q_1q_2 {\widehat{w}}\Psi \rangle \!\rangle \right| \nonumber \\&\qquad + N\left| \langle \!\langle \Psi ,q_1p_2(\Delta _1 h_{0,\beta }(x_1-x_2))q_1q_2{\widehat{w}}\Psi \rangle \!\rangle \right| \nonumber \\&\quad \le \Vert U_{0,\beta }\Vert _\infty N\Vert q_1\Psi \Vert \;\Vert {\widehat{w}}q_1q_2\Psi \Vert \nonumber \\&\qquad +\,N\left| \langle \!\langle \nabla _1q_1 p_2\Psi ,(\nabla _1 h_{0,\beta }(x_1-x_2)){\widehat{w}}q_1q_2\Psi \rangle \!\rangle \right| \nonumber \\&\qquad +\, N\left| \langle \!\langle \Psi , {\widehat{w}}_1q_1p_2(\nabla _1 h_{0,\beta }(x_1-x_2))\nabla _1q_1q_2\Psi \rangle \!\rangle \right| \nonumber \\&\quad \le N \Vert U_{0,\beta }\Vert _\infty \Vert q_1\Psi \Vert \;\Vert {\widehat{w}}q_1q_2\Psi \Vert \end{aligned}$$
  90 $$\begin{aligned}&\qquad +\,N\left| \langle \!\langle \mathbb {1}_{{\mathcal {A}}^{(d)}_{1}}\nabla _1q_1\Psi ,p_2(\nabla _1 h_{0,\beta }(x_1-x_2)){\widehat{w}}q_1q_2\Psi \rangle \!\rangle \right| \end{aligned}$$
  91 $$\begin{aligned}&\qquad +\,N\left| \langle \!\langle \nabla _1q_1\Psi ,\mathbb {1}_{\overline{{\mathcal {A}}}^{(d)}_{1}}p_2(\nabla _1 h_{0,\beta }(x_1-x_2))q_1q_2{\widehat{w}}\Psi \rangle \!\rangle \right| \end{aligned}$$
  92 $$\begin{aligned}&\qquad +\, N\left| \langle \!\langle \Psi , {\widehat{w}}_1q_1p_2(\nabla _1 h_{0,\beta }(x_1-x_2))q_2\mathbb {1}_{\overline{{\mathcal {A}}}^{(d)}_{1}}\nabla _1q_1\Psi \rangle \!\rangle \right| \end{aligned}$$
  93 $$\begin{aligned}&\qquad +\, N\left| \langle \!\langle \Psi , {\widehat{w}}_1q_1p_2(\nabla _1 h_{0,\beta }(x_1-x_2))q_2\mathbb {1}_{{\mathcal {A}}^{(d)}_{1}}\nabla _1q_1\Psi \rangle \!\rangle \right| . \end{aligned}$$
  In the following, we will estimate each term separately.

**Estimate of** (89)**:**

Lemma 4.4 and Definition 7.1 yields the bound

$$\begin{aligned} (89)\le C \langle \!\langle \Psi ,{\widehat{n}}\Psi \rangle \!\rangle . \end{aligned}$$

**Estimate of** (90)**:**

For (90) we use that $\nabla _2 h_{0,\beta }(x_1-x_2)=-\nabla _1 h_{0,\beta }(x_1-x_2)$, Cauchy Schwarz and $ab\le a^2+b^2$ and get

94 $$\begin{aligned} (90)\le \Vert \mathbb {1}_{{\mathcal {A}}^{(d)}_{1}}\nabla _1q_1\Psi \Vert ^2+N^2\Vert p_2(\nabla _2 h_{0,\beta }(x_1-x_2)){\widehat{w}}q_1q_2\Psi \Vert ^2. \end{aligned}$$

$\Vert \mathbb {1}_{{\mathcal {A}}^{(d)}_{1}}\nabla _1q_1 \Psi \Vert ^2$ can be bounded using Lemma 7.9.

Integration by parts and Lemma 4.2 (c) as well as $(a+b)^2\le 2a^2+2b^2$ gives for the second summand

95 $$\begin{aligned} N^2&\Vert p_1(\nabla _1h_{0,\beta }(x_1-x_2))q_1q_2{\widehat{w}}\Psi \Vert ^2 \nonumber \\&\le 2N^2\Vert p_1h_{0,\beta }(x_1-x_2)\nabla _1q_1q_2{\widehat{w}}\Psi \Vert ^2 \nonumber \\&\quad +\,2N^2\Vert |\varphi (x_1)\rangle \langle \nabla _1\varphi (x_1)|h_{0,\beta }(x_1-x_2)q_1q_2{\widehat{w}}\Psi \Vert ^2 \nonumber \\&\le CN ^2\Vert p_1h_{0,\beta }(x_1-x_2)q_2(p_1{\widehat{w}}_1 +q_1{\widehat{w}})\mathbb {1}_{{\mathcal {A}}^{(d)}_{1}}\nabla _1q_1\Psi \Vert ^2 \end{aligned}$$

96 $$\begin{aligned}&\quad +\, CN ^2\Vert p_1h_{0,\beta }(x_1-x_2)q_2p_1{\widehat{w}}_1 \mathbb {1}_{\overline{{\mathcal {A}}}^{(d)}_{1}}\nabla _1q_1\Psi \Vert ^2 \end{aligned}$$

97 $$\begin{aligned}&\quad +\, CN ^2\Vert p_1h_{0,\beta }(x_1-x_2)q_2q_1{\widehat{w}} \mathbb {1}_{\overline{{\mathcal {A}}}^{(d)}_{1}}\nabla _1q_1\Psi \Vert ^2 \end{aligned}$$

98 $$\begin{aligned}&\quad +\,2N^2\Vert |\varphi (x_1)\rangle \langle \nabla _1\varphi (x_1)|h_{0,\beta } (x_1-x_2)q_1q_2{\widehat{w}}\Psi \Vert ^2. \end{aligned}$$

For (95) we use Lemma 4.4, Lemma 4.2 (e) with Lemma 7.2 (c) and then Lemma 7.9.

$$\begin{aligned} (95)&\le CN ^2\Vert p_1h_{0,\beta }(x_1-x_2)\Vert _{\text {op}}^2\Vert \mathbb {1}_{{\mathcal {A}}^{(d)}_{1}} \nabla _1q_1\Psi \Vert ^2 \\&\le {\mathcal {K}}(\varphi ,A_t) \Big ( \left\langle \!\left\langle \Psi ,{\widehat{n}}^{\varphi }\Psi \right\rangle \!\right\rangle +N^{-1/6} \ln (N) \\&\quad +\,\inf \left\{ \left| {\mathcal {E}}_{V_N}(\Psi )-{\mathcal {E}}_{4 \pi }^{GP}(\varphi )\right| , \left| {\mathcal {E}}_{W_\beta }(\Psi )- {\mathcal {E}}_{b_{W_{\beta }}}^{GP}(\varphi ) \right| + N^{-2 \beta } \ln (N) \right\} \Big ). \end{aligned}$$

Let $s_1 \in \lbrace p_1, q_1 \rbrace$ and let ${\widehat{d}} \in \lbrace {\widehat{w}}, {\widehat{w}}_1 \rbrace$. Note that $\Vert {\widehat{d}} \Vert _{\text {op}}= \Vert {\widehat{w}} \Vert _{\text {op}}$. Then, (96) and (97) can be estimated with help of Lemma 7.4, part (b)

$$\begin{aligned}&(96), (97) \le CN ^2 \Vert \nabla _1 q_1 \Psi \Vert \Vert \mathbb {1}_{\overline{{\mathcal {A}}}^{(d)}_{1}} {\widehat{d}} s_1 q_2 h_{0,\beta }(x_1-x_2) p_1h_{0,\beta }(x_1-x_2)q_2s_1{\widehat{d}} \mathbb {1}_{\overline{{\mathcal {A}}}^{(d)}_{1}}\nabla _1q_1\Psi \Vert \\&\quad \le C_p N^{2+ \frac{1-2d}{2}\frac{p-1}{p}} \Vert \nabla _1 q_1 \Psi \Vert \Vert \nabla _1{\widehat{d}} s_1 q_2 h_{0,\beta }(x_1-x_2) p_1h_{0,\beta }(x_1-x_2)q_2s_1{\widehat{d}}\mathbb {1}_{\overline{{\mathcal {A}}}^{(d)}_{1}}\nabla _1q_1\Psi \Vert ^{\frac{p-1}{p}} \\&\qquad \times \Vert {\widehat{d}} s_1 q_2 h_{0,\beta }(x_1-x_2) p_1h_{0,\beta }(x_1-x_2)q_2s_1{\widehat{d}}\mathbb {1}_{\overline{{\mathcal {A}}}^{(d)}_{1}}\nabla _1q_1\Psi \Vert ^{\frac{1}{p}} \\&\quad \le C_p N^{2+ \frac{1-2d}{2}\frac{p-1}{p}} \Vert \nabla _1 q_1 \Psi \Vert \Vert {\widehat{w}}\Vert _{\text {op}} \Vert p_1 h_{0,\beta }(x_1-x_2)\Vert _{\text {op}} \Vert \mathbb {1}_{\overline{{\mathcal {A}}}^{(d)}_{1}}\nabla _1q_1\Psi \Vert \\&\qquad \times \Vert \nabla _1{\widehat{d}} s_1 q_2 h_{0,\beta }(x_1-x_2) p_1\Vert _{\text {op}}^{\frac{p-1}{p}} \Vert {\widehat{d}} s_1 q_2 h_{0,\beta }(x_1-x_2) p_1\Vert _{\text {op}}^{\frac{1}{p}} \\&\quad \le C_p {\mathcal {K}}(\varphi ,A_t) N^{1+\frac{1-2d}{2}\frac{p-1}{p}} \Vert {\widehat{w}}\Vert _{\text {op}}^2 \Vert \nabla _1 s_1 h_{0,\beta }(x_1-x_2) p_1\Vert _{\text {op}}^{\frac{p-1}{p}} \Vert h_{0,\beta }(x_1-x_2) p_1\Vert _{\text {op}}^{\frac{1}{p}} \\&\quad \le C_p {\mathcal {K}}(\varphi ,A_t) N^{1+\frac{1-2d}{2}\frac{p-1}{p}} \Vert {\widehat{w}}\Vert _{\text {op}}^2 \left( \Vert \nabla \varphi \Vert \Vert h_{0,\beta }\Vert + \Vert \nabla _1 h_{0,\beta }\Vert \right) ^{\frac{p-1}{p}} \Vert h_{0,\beta }\Vert ^{\frac{1}{p}} \\&\quad \le C_p {\mathcal {K}}(\varphi ,A_t) \Vert {\widehat{w}}\Vert _{\text {op}}^2 (1 +\ln (N))^{ \frac{p-1}{2p}} N^{\frac{1-2d}{2}\frac{p-1}{p}}. \end{aligned}$$

Here, we used, for $s_1 \in \lbrace p_1, 1- p_1 \rbrace$,

$$\begin{aligned} \Vert \nabla _1 s_1 h_{0,\beta }(x_1-x_2) p_1\Vert _{\text {op}}&\le \Vert \nabla _1 p_1 h_{0,\beta }(x_1-x_2) p_1\Vert _{\text {op}} +\Vert \nabla _1 h_{0,\beta }(x_1-x_2) p_1\Vert _{\text {op}} \\&\le \Vert \varphi \Vert _\infty \left( \Vert \nabla \varphi \Vert \Vert h_{0,\beta }\Vert + \Vert \nabla h_{0,\beta }\Vert \right) \end{aligned}$$

and then applied Lemma 4.2 (e). With $\Vert {\widehat{w}}\Vert _{\text {op}} \le N^{1/3}$, we obtain

$$\begin{aligned} {}(96)+(97)&\le C_p {\mathcal {K}}(\varphi ,A_t) \ln (N)^{ \frac{p-1}{2p}} N^{\frac{2}{3}} N^{\frac{1-2d}{2}\frac{p-1}{p}} \\&\le C_p {\mathcal {K}}(\varphi ,A_t) \ln (N)^{ \frac{1}{2}} N^{2+\frac{d}{p}-d-\frac{1}{2p}}. \end{aligned}$$

For $p=2$ and $d \ge \max \{7, 3 + \beta \}$, we obtain

$$\begin{aligned} {}(96)+(97)&\le C_2 {\mathcal {K}}(\varphi ,A_t) N^{-1} \le {\mathcal {K}}(\varphi ,A_t) N^{-1}. \end{aligned}$$

Line (98) can be bounded by

$$\begin{aligned} (98)&\le C N^2\Vert h_{0,\beta }(x_1-x_2)\nabla _1p_1\Vert _{\text {op}}^2\;\Vert q_1q_2{\widehat{w}}\Psi \Vert ^2\\&\le CN ^2\Vert h_{0,\beta }\Vert ^2 \Vert \nabla \varphi \Vert _\infty ^2 \Vert {\widehat{n}} \, {\widehat{w}} \Vert _{\text {op}}^2 \Vert q_1 \Psi \Vert ^2 \\&\le C \Vert \nabla \varphi \Vert _\infty ^2 \langle \!\langle \Psi {\widehat{n}}\Psi \rangle \!\rangle . \end{aligned}$$

**Estimate of** (91) **and** (92)**:**

For (91) and (92) we use Cauchy–Schwarz and then Sobolev inequality as in Lemma 7.4 implies that for any $p > 1$, there exists a constant $C_p$ such that

$$\begin{aligned} (91)+(92)&\le N\left\| \nabla _1q_1\Psi \right\| \left\| \mathbb {1}_{\overline{{\mathcal {A}}}^{(d)}_{1}}p_2(\nabla _1 h_{0,\beta }(x_1-x_2))q_1q_2{\widehat{w}}\Psi \right\| \\&\quad +\,N\left\| \nabla _1q_1\Psi \right\| \left\| \mathbb {1}_{\overline{{\mathcal {A}}}^{(d)}_{1}}q_2(\nabla _1 h_{0,\beta }(x_1-x_2))q_1p_2{\widehat{w}}_1\Psi \right\| \\&\le C_p N\Vert \nabla _1q_1\Psi \Vert \; N^{\frac{1-2d}{2} \frac{p-1}{p}} \Vert \nabla _1 p_2(\nabla _1 h_{0,\beta }(x_1-x_2))q_1q_2{\widehat{w}}\Psi \Vert ^{\frac{p-1}{p} } \\&\quad \times \Vert p_2(\nabla _1 h_{0,\beta }(x_1-x_2))q_1q_2{\widehat{w}}\Psi \Vert ^{1/p} \\&\quad +\,C_pN\Vert \nabla _1q_1\Psi \Vert \; N^{\frac{1-2d}{2} \frac{p-1}{p}} \Vert \nabla _1 q_2(\nabla _1 h_{0,\beta }(x_1-x_2))q_1p_2{\widehat{w}}_1\Psi \Vert ^{ \frac{p-1}{p} } \\&\quad \times \Vert q_2(\nabla _1 h_{0,\beta }(x_1-x_2))q_1p_2{\widehat{w}}_1\Psi \Vert ^{1/p}. \end{aligned}$$

Using Lemma 4.2, Lemma 4.4, Corollary 4.5 and Lemma 7.2, we obtain

$$\begin{aligned}&\Vert \nabla _1 p_2(\nabla _1 h_{0,\beta }(x_1-x_2))q_1q_2{\widehat{w}}\Psi \Vert \\&\quad \le \Vert p_2(\Delta _1 h_{0,\beta }(x_1-x_2))q_1q_2{\widehat{w}}\Psi \Vert + \Vert p_2 (\nabla _1 h_{0,\beta }(x_1-x_2))\nabla _1q_1q_2{\widehat{w}}\Psi \Vert \\&\quad \le C \left( \Vert p_2(W_\beta -U_{0,\beta })(x_1-x_2)\Vert _{\text {op}}+ \Vert p_2 ( \nabla _1 h_{0,\beta }(x_1-x_2))\Vert _{\text {op}}\right) \\&\quad \le C \Vert \varphi \Vert _\infty \left( N^{-1+\beta } + N^{-1} (\ln (N))^{1/2} \right) , \end{aligned}$$

and similarly

$$\begin{aligned}&\Vert \nabla _1q_2(\nabla _1 h_{0,\beta }(x_1-x_2))q_1p_2{\widehat{w}}_1\Psi \Vert \\&\quad \le \Vert q_2(\Delta _1 h_{0,\beta }(x_1-x_2))q_1p_2{\widehat{w}}_1\Psi \Vert + \Vert q_2(\nabla _1 h_{0,\beta }(x_1-x_2))\nabla _1q_1p_2{\widehat{w}}_1\Psi \Vert \\&\quad \le C \left( \Vert p_2(W_\beta -U_{0,\beta })(x_1-x_2)\Vert _{\text {op}}+ \Vert {\widehat{w}}_1\Vert _{\text {op}} \Vert p_2 ( \nabla _1 h_{0,\beta }(x_1-x_2))\Vert _{\text {op}}\right) \\&\quad \le C \Vert \varphi \Vert _\infty \left( N^{-1+\beta } + \Vert {\widehat{w}}\Vert _{\text {op}} N^{-1} (\ln (N))^{1/2} \right) . \end{aligned}$$

Moreover, we estimate

$$\begin{aligned} \Vert p_2 (\nabla _1 h_{0,\beta }(x_1-x_2))q_1q_2{\widehat{w}}\Psi \Vert&\le C\Vert \varphi \Vert _\infty \Vert \nabla _1 h_{0,\beta }\Vert _2 \le C\Vert \varphi \Vert _\infty N^{-1} (\ln (N))^{1/2} \\ \Vert q_2 (\nabla _1 h_{0,\beta }(x_1-x_2))q_1p_2{\widehat{w}}_1 \Psi \Vert&\le C\Vert \varphi \Vert _\infty \Vert \nabla _1 h_{0,\beta }\Vert _2 \le C \Vert \varphi \Vert _\infty N^{-1} (\ln (N))^{1/2}. \end{aligned}$$

Thus if we choose $p=2$ and recall that $\xi < 1/3$ and $d \ge \max \{7, 3 + \beta \}$, we obtain

$$\begin{aligned} (91)+(92)&\le C_2 \Vert \varphi \Vert _\infty N^{1+\frac{1-2d}{4} } \left( N^{-1+\beta } + \Vert {\widehat{w}}\Vert _{\text {op}} N^{-1} (\ln (N))^{1/2} \right) ^{\frac{1}{2}} \left( N^{-1} (\ln (N))^{1/2} \right) ^{1/2} \\&\le C_2 \Vert \varphi \Vert _\infty \left( N^{\frac{1}{2} + \beta - d} (\ln (N))^{1/2} + N^{\frac{1}{3} + \frac{1}{2} - d} \ln (N) \right) ^{\frac{1}{2}} \le C \Vert \varphi \Vert _\infty N^{-1}. \end{aligned}$$

**Estimate of** (93)**:**

For (93) we use Lemma 4.6 with $\Omega =\mathbb {1}_{{\mathcal {A}}^{(d)}_{1}}\nabla _1q_1\Psi$, $O_{1,2}=Nq_2(\nabla _2 h_{0,\beta }(x_1-x_2))p_2$ and $\chi = {\widehat{w}}_1 q_1\Psi$.

99 $$\begin{aligned} (93)&\le \Vert \mathbb {1}_{{\mathcal {A}}^{(d)}_{1}}\nabla _1q_1\Psi \Vert ^2 \end{aligned}$$

100 $$\begin{aligned}&\quad +\, 2 N\Vert q_2(\nabla _2 h_{0,\beta }(x_1-x_2)) {\widehat{w}}_1 q_1p_2\Psi \Vert ^2 \end{aligned}$$

101 $$\begin{aligned}&\quad +\,N^2\big |\langle \!\langle \Psi ,q_1q_3 {\widehat{w}}_1 (\nabla _2 h_{0,\beta }(x_1-x_2)) p_2p_3(\nabla _3 h_{0,\beta }(x_1-x_3)) {\widehat{w}}_1 q_1q_2\Psi \rangle \!\rangle \big |. \end{aligned}$$

Line (100) is bounded by

$$\begin{aligned} {}(100)&\le CN \Vert (\nabla _2 h_{0,\beta }(x_1-x_2))p_2 \Vert _{\text {op}}^2 \Vert {\widehat{w}}_1 {\widehat{n}} \Vert _{\text {op}}^2 \\&\le C \Vert \varphi \Vert _\infty ^2 N \Vert \nabla _2 h_{0,\beta }(x_1-x_2) \Vert ^2 \le C \Vert \varphi \Vert _\infty ^2 N^{-1} \ln (N). \end{aligned}$$

(99) $+$ (101) is bounded by

$$\begin{aligned} \Vert \mathbb {1}_{{\mathcal {A}}^{(d)}_{1}}\nabla _1q_1\Psi \Vert ^2+N^2\Vert p_2 (\nabla _2 h_{0,\beta }(x_1-x_2)) {\widehat{w}}_1 q_1 q_2\Psi \Vert ^2. \end{aligned}$$

Both terms can be controlled analogously to (94).

**Complete estimate:**

In total, we obtain

$$\begin{aligned}&N|\langle \!\langle \Psi , p_1q_2Z^\varphi _\beta (x_1,x_2) {\widehat{w}} q_1q_2\Psi \rangle \!\rangle | \le {\mathcal {K}}(\varphi ,A_t) \Big ( \langle \!\langle \Psi ,{\widehat{n}}\Psi \rangle \!\rangle + N^{-1/6} \ln (N) \\&\quad +\,\inf \left\{ \left| {\mathcal {E}}_{V_N}(\Psi )-{\mathcal {E}}_{4 \pi }^{GP}(\varphi )\right| , \left| {\mathcal {E}}_{W_\beta }(\Psi )- {\mathcal {E}}_{b_{W_{\beta }}}^{GP}(\varphi ) \right| + N^{-2 \beta } \ln (N) \right\} \Big ). \end{aligned}$$

$$\square $$

To estimate $\gamma _b^<$ we recall that ${\widehat{w}} \in \lbrace N {\widehat{m}}^a_{-1},N {\widehat{m}}^b_{-2} \rbrace$ with $w(k) < n(k)^{-1}$ and $\Vert {\widehat{w}}_1 \Vert _{\text {op}} \le C \Vert {\widehat{w}}\Vert _{\text {op}}\le CN ^{\xi }$. Lemma 7.7, $\Vert {\widehat{n}} - {\widehat{m}} \Vert _{op} \le CN ^{- \xi }$ and $\xi < 1/3$ imply

$$\begin{aligned}&\gamma _b^< (\Psi ,\varphi ) \le {\mathcal {K}}(\varphi , A_t) \Big ( \alpha ^<(\Psi , \varphi ) + N^{-1/3} \\&\quad +\, \inf _{ \min \{\beta , 1/2 \}>\beta _1>0} \, \inf _{\eta >0} \left( N^{ \eta - 2 \beta _1} \ln (N)^2 + N^{-1+ \xi + 2 \beta _1} + N^{ 2 \xi - \eta } \right) \Big ). \end{aligned}$$

In addition, we have the improved bound

$$\begin{aligned}&\gamma _b^< (\Psi ,\varphi ) \le {\mathcal {K}}(\varphi , A_t) \left( \alpha ^<(\Psi , \varphi ) + N^{-\xi } + N^{-1+ \xi + 2 \beta } + (N^{-1/6} + N^{- 2 \beta }) \ln (N) \right) \end{aligned}$$

for al $$\beta < 1/2$$.

**Control of**$\gamma _c^<$ With Definition 2.1 and (76) we estimate

$$\begin{aligned} | \gamma _c^<(\Psi ,\varphi ) |&\le N \big | N \Vert W_{\beta } \Vert _1 - b_{W_{\beta }} \big | \; \big | \langle \!\langle \Psi , (q_1 | \varphi (x_1) |^2 {\widehat{m}}^a p_1 - p_1 {\widehat{m}}^a | \varphi (x_1) |^2 q_1 ) \Psi \rangle \!\rangle \big | \\&\le N \big | N \Vert W_{\beta } \Vert _1 - b_{W_{\beta }} \big | \; \Vert \varphi \Vert _{\infty }^2 \Vert {\widehat{m}}^a \Vert _{\text {op}} \Vert q_1 \Psi \Vert \\&\le {\mathcal {K}}(\varphi ,A_t) N^{-1 + \xi } \ln (N). \end{aligned}$$

Collecting all the estimates for $\gamma _a^<$, $\gamma _b^<$ and $\gamma _c^<$ then proves Lemma 6.6.

#### Proof of Lemma 6.6

Let the assumptions of Lemma 6.6 be satisfied. By the previous we have

$$\begin{aligned}&\sum _{k \in \{a, b, c\}} | \gamma ^<_k(\Psi _t,\varphi _t) | \le {\mathcal {K}}(\varphi _t,A_t) \Big ( \alpha ^<(\Psi _t, \varphi _t) + N^{- \xi } + \left( N^{-1/6} + N^{- 2 \beta } \right) \ln (N) \\&\quad +\, \inf _{ \min \{\beta , 1/2 \}>\beta _1>0} \, \inf _{\eta >0} \left( N^{ \eta - 2 \beta _1} \ln (N)^2 + N^{-1+ \xi + 2 \beta _1} + N^{ 2 \xi - \eta } \right) \Big ) \end{aligned}$$

and the slightly stronger estimate

$$\begin{aligned}&\sum _{k \in \{a, b, c\}} | \gamma ^<_k(\Psi _t,\varphi _t) | \le {\mathcal {K}}(\varphi _t,A_t) \left( \alpha ^<(\Psi _t, \varphi _t) + N^{- \xi } + N^{-1 + \xi + 2 \beta } \right. \\&\quad \left. +\,\left( N^{-1/6} + N^{- 2 \beta } \right) \ln (N) \right) \end{aligned}$$

if $\beta < 1/2$. Inequality (49) follows for $1/3 \le \beta$ from the first bound (with $\beta _1 = 3/10$ and $\eta = 3/10$) and for $1/12 \le \beta < 1/3$ from the second relation. Moreover, if we choose $\beta < 1/12$ and $\xi = 1/6$ we obtain (48). $\quad \square$

### Proof of Lemma 6.13

Next, we prove Lemma 6.13. We will proceed in a similar way as in the previous section and consecutively estimate the functionals $\gamma _i$ with $i \in \{ a,b,c,d,e,f \}$. In the rest of this section we assume that $V_N \in {\mathcal {V}}_N$, $\varphi \in H^{3}({\mathbb {R}}^2,{\mathbb {C}})$ with $\Vert \varphi \Vert =1$ and that $\Psi \in L^2_{s}({\mathbb {R}}^{2N}, {\mathbb {C}}) \cap H^2({\mathbb {R}}^{2N}, {\mathbb {C}})$ with $\Vert \Psi \Vert =1$ such that ${\mathcal {E}}_{V_N}(\Psi ) \le C$.

For the most involved scaling which is induced by $V_N$, we need to control $\Vert p_1 V_N (x_1 - x_2) \Psi \Vert$.

#### Lemma 7.8

Let $V_N \in {\mathcal {V}}_N$, $\Psi \in L_s^2 ({\mathbb {R}}^{2N}, {\mathbb {C}}) \cap H^1({\mathbb {R}}^{2N}, {\mathbb {C}})$, $\varphi \in H^{3}({\mathbb {R}}^2,{\mathbb {C}})$ with $\Vert \varphi \Vert = 1$ and ${\mathcal {E}}_{V_N}(\Psi ) \le C$. Then

102 $$\begin{aligned} \Vert p_1 V_N (x_1 - x_2) \Psi \Vert \le {\mathcal {K}}(\varphi , A_t) N^{-\frac{1}{2}}. \end{aligned}$$

#### Proof

We estimate

$$\begin{aligned} \Vert p_1 V_N(x_1-x_2) \Psi \Vert&= \Vert p_1 \mathbb {1}_{\text {supp}(V_N)}(x_1-x_2) V_N(x_1-x_2) \Psi \Vert \\&\le \Vert p_1 \mathbb {1}_{\text {supp}(V_N)}(x_1-x_2) \Vert _{\text {op}} \Vert V_N(x_1-x_2) \Psi \Vert . \end{aligned}$$

With Lemma 4.2 (e) we get

$$\begin{aligned} \Vert p_1 \mathbb {1}_{\text {supp}(V_N)} (x_1-x_2)\Vert _{\text {op}}^2\le \Vert \varphi \Vert _\infty ^2 \Vert \mathbb {1}_{\text {supp}(V_N)} \Vert _1 \le C \Vert \varphi \Vert _\infty ^2 e^{-2N}. \end{aligned}$$

Using

$$\begin{aligned} C \ge {\mathcal {E}}_{V_N}(\Psi )= \Vert \nabla \Psi \Vert ^2 + \frac{(N-1)}{2} \Vert \sqrt{V_N}(x_1-x_2) \Psi \Vert ^2 + \langle \!\langle \Psi , A_t(x_1) \Psi \rangle \!\rangle \end{aligned}$$

as well as

$$\begin{aligned} \Vert V_N(x_1-x_2) \Psi \Vert ^2&= \Vert \sqrt{V_N} (x_1-x_2)\sqrt{ V_N}(x_1-x_2) \Psi \Vert ^2 \\&\le \Vert \sqrt{V_N}\Vert _{\infty }^2 \Vert \sqrt{V_N}(x_1-x_2) \Psi \Vert ^2 \\&\le C e^{2N} \frac{{\mathcal {E}}_{V_N}(\Psi )+ \Vert A_t \Vert _\infty }{N} \le C (1+ \Vert A_t \Vert _\infty ) \frac{e^{2N}}{N}, \end{aligned}$$

we obtain

$$\begin{aligned} \Vert p_1 V_N (x_1 - x_2) \Psi \Vert \le {\mathcal {K}}(\varphi , A_t) N^{-\frac{1}{2}}. \end{aligned}$$

$$\square $$

**Control of** $$\gamma _a$$

In total analogy to (73) we get

$$\begin{aligned} | \gamma _a(\Psi ,\varphi ) | \le C \Vert \dot{A}_t\Vert _\infty ( \langle \!\langle \Psi ,{\widehat{n}} \Psi \rangle \!\rangle + N^{-\frac{1}{2}} ) \le C \Vert \dot{A}_t\Vert _\infty ( \langle \!\langle \Psi ,{\widehat{m}} \Psi \rangle \!\rangle + N^{- \xi } ). \end{aligned}$$

With Definition 6.8 and (58) we have

$$\begin{aligned} | \gamma _a(\Psi ,\varphi ) |&\le {\mathcal {K}}(\varphi , A_t) ( \alpha (\Psi , \varphi ) + N^{- \xi } + N^{- \mu + \xi } \ln (N) ). \end{aligned}$$

**Control of**$$\gamma _b$$

Recall that

$$\begin{aligned} \gamma _b(\Psi , \varphi )&= -N(N-1)\mathfrak {I}\left( \langle \!\langle \Psi , {\widetilde{Z}}_{\mu }^{\varphi }(x_1,x_2){\widehat{r}}\, \Psi \rangle \!\rangle \right) \\&\quad -\,N(N-1)\mathfrak {I}\left( \langle \!\langle \Psi ,g_{\mu }(x_{1}-x_{2}) {\widehat{r}}\,{\mathcal {Z}}^{\varphi } (x_1,x_2) \Psi \rangle \!\rangle \right) . \end{aligned}$$

Estimate (102) yields to the bound $\Vert p_1 {\mathcal {Z}}^{\varphi } (x_1,x_2)\Psi \Vert \le {\mathcal {K}}(\varphi , A_t) N^{-1/2}$. Thus, if we use Lemma 5.5 and $|| {\widehat{m}}^a ||_{\text {op}} + || {\widehat{m}}^b ||_{\text {op}} \le CN ^{-1+ \xi }$ [see (76)] the second line is controlled by

$$\begin{aligned}&N^2 \big ( \Vert {\widehat{m}}^a \Vert _{\text {op}} + \Vert {\widehat{m}}^b \Vert _{\text {op}} \big ) \Vert g_{\mu }(x_{1}-x_{2}) p_{1}\Vert _{\text {op}} \Vert p_1{\mathcal {Z}}^{\varphi } (x_1,x_2)\Psi \Vert \\&\quad \le {\mathcal {K}}(\varphi , A_t) N^{1/2 + \xi }\Vert g_{\mu }\Vert \le {\mathcal {K}}(\varphi , A_t) N^{\xi -1/2- \mu } \ln (N). \end{aligned}$$

The first line of $\gamma _b$ can be bounded with (62) and $f_{\mu }=1-g_{\mu }$ by

103 $$\begin{aligned}&N(N-1)|\mathfrak {I}\left( \langle \!\langle \Psi , {\widetilde{Z}}_{\mu }^{\varphi }(x_1,x_2){\widehat{r}}\, \Psi \rangle \!\rangle \right) | \nonumber \\&\quad \le N^2 | \mathfrak {I}\left( \langle \!\langle \Psi , \left( M_{\mu }(x_1-x_2)f_{\mu }(x_{1}-x_{2})\right. \right. \nonumber \\&\qquad \left. \left. -\,\frac{N}{N-1} \Vert M_{\mu }f_{\mu } \Vert _1 \left( |\varphi (x_1)|^2 +|\varphi (x_2)|^2 \right) \right) {\widehat{r}} \Psi \rangle \!\rangle \right) | \end{aligned}$$

104 $$\begin{aligned}&\qquad +\,\frac{N^2}{N-1} |\langle \!\langle \Psi , \left( \Vert N M_{\mu }f_{\mu } \Vert _1- 4\pi \right) \left( |\varphi (x_1)|^2+|\varphi (x_2)|^2\right) {\widehat{r}} \Psi \rangle \!\rangle | \end{aligned}$$

105 $$\begin{aligned}&\qquad +\,\frac{N^2}{N-1}|\langle \!\langle \Psi , 4\pi \left( |\varphi (x_1)|^2+ |\varphi (x_2)|^2\right) g_{\mu }(x_{1}-x_{2}) {\widehat{r}}\Psi \rangle \!\rangle |. \end{aligned}$$

Since $M_{\mu }f_{\mu } \in {\mathcal {W}}_\mu$, (103) is of the same form as $\gamma ^<_b(\Psi ,\varphi )$. By means of Lemma 7.7, $\Vert {\widehat{n}} - {\widehat{m}} \Vert _{op} \le C N^{- \xi }$ and (58), we obtain

$$\begin{aligned} | (103) |&\le {\mathcal {K}}(\varphi , A_t) \Big ( \alpha (\Psi , \varphi ) + N^{- \xi } + \left( N^{-1/6} + N^{- \mu + \xi } \right) \ln (N) \\&\quad +\, \inf _{ \min \{\beta , 1/2 \}>\beta _1>0} \, \inf _{\eta >0} \left( N^{ \eta - 2 \beta _1} \ln (N)^2 + N^{-1+2 \beta _1 + \xi } + N^{ 2 \xi - \eta } \right) \Big ). \end{aligned}$$

Using Lemma 5.5 (h), the second term is controlled by

$$\begin{aligned} {}(104) \le C \Vert \varphi \Vert _\infty ^2 N \, \big | N\Vert M_{\mu } f_\mu \Vert _1-4 \pi \big | \, \Vert {\hat{r}}\Vert _{\text {op}} \le C \Vert \varphi \Vert _\infty ^2 N^{-1+ \xi } \ln (N). \end{aligned}$$

The last term is controlled by

$$\begin{aligned} {}(105)&\le CN \Vert \varphi \Vert _\infty ^2\Vert g_{\mu }(x_{1}-x_{2}) p_{1}\Vert _{\text {op}} \big ( \Vert {\widehat{m}}^a\Vert _{\text {op}} + \Vert {\widehat{m}}^b\Vert _{\text {op}} \big ) \le C\Vert \varphi \Vert _\infty ^3N^{-1-\mu +\xi } \ln (N) \end{aligned}$$

which implies the bound

$$\begin{aligned} |\gamma _b(\Psi , \varphi )|&\le {\mathcal {K}}(\varphi , A_t) \Big ( \alpha (\Psi , \varphi ) + N^{- \xi } + \left( N^{-1 + \xi } + N^{-1/6} + N^{- \mu + \xi } \right) \ln (N) \\&\quad +\, \inf _{ \min \{\beta , 1/2 \}>\beta _1>0} \, \inf _{\eta >0} \left( N^{ \eta - 2 \beta _1} \ln (N)^2 + N^{-1+2 \beta _1 + \xi } + N^{ 2 \xi - \eta } \right) \Big ). \end{aligned}$$

**Control of**$$\gamma _c$$

Recall that

$$\begin{aligned} \gamma _c(\Psi , \varphi )&=-4N(N-1) \langle \!\langle \Psi , (\nabla _1g_{\mu }(x_1-x_2))\nabla _1 {\widehat{r}}\Psi \rangle \!\rangle . \end{aligned}$$

Using ${\widehat{r}}=(p_2+q_2){\widehat{r}}=p_2{\widehat{r}}+p_1q_2{\widehat{m}}^a$ and $\nabla _1g_{\mu }(x_1-x_2)=-\nabla _2g_{\mu }(x_1-x_2)$, integration by parts yields to

106 $$\begin{aligned} |\gamma _c(\Psi ,\varphi )|&\le 4N^2|\langle \!\langle \Psi , g_{\mu }(x_1-x_2)\nabla _1\nabla _2 ( p_2{\widehat{r}}+p_1q_2{\widehat{m}}^a ) \Psi \rangle \!\rangle | \end{aligned}$$

107 $$\begin{aligned}&\quad +\, 4N^2|\langle \!\langle \nabla _2\Psi , g_{\mu }(x_1-x_2)\nabla _1 p_2{\widehat{r}}\Psi \rangle \!\rangle | \end{aligned}$$

108 $$\begin{aligned}&\quad +\,4N^2|\langle \!\langle \nabla _2\Psi , g_{\mu }(x_1-x_2)\nabla _1 p_1q_2{\widehat{m}}^a\Psi \rangle \!\rangle |. \end{aligned}$$

We begin with

$$\begin{aligned} (106)&\le CN ^2 \Vert g_\mu \Vert \Vert \nabla \varphi \Vert _\infty \left( \Vert \nabla _1 {\widehat{r}} \Psi \Vert + \Vert \nabla _ 2 q_2 {\widehat{m}}^a\Psi \Vert \right) \\&\le CN ^{1-\mu } \ln (N) \Vert \nabla \varphi \Vert _\infty \left( \Vert \nabla _1 {\widehat{r}} \Psi \Vert + \Vert \nabla _ 2 q_2 {\widehat{m}}^a\Psi \Vert \right) . \end{aligned}$$

Let $s_1,t_1\in \lbrace p_1,q_1 \rbrace$. Inserting the identity $1 = p_1 + q_1$, we obtain for $a \in \lbrace -1,0,1 \rbrace$,

$$\begin{aligned} \Vert \nabla _1{\widehat{r}}\Psi \Vert&\le C \sup _{s_1,t_1, a} \Vert {\widehat{r}}_a s_1 \nabla _1 t_1 \Psi \Vert \le C \sup _{t_1, a} \Vert {\widehat{r}}_a\Vert _{\text {op}} \Vert \nabla _1 t_1 \Psi \Vert \le CN ^{-1 + \xi }. \end{aligned}$$

In analogy $\Vert \nabla _ 2 q_2 {\widehat{m}}^a\Psi \Vert \le C \Vert {\widehat{m}}^a \Vert _{\text {op}} \le CN ^{-1 + \xi }$. This yields the bound

$$\begin{aligned} (106)\le {\mathcal {K}}(\varphi , A_t) N^{-\mu + \xi } \ln ( N). \end{aligned}$$

Furthermore, (107) is bounded by

109 $$\begin{aligned} (107)&\le 4N^2\Vert \nabla _2 \Psi \Vert \; \Vert g_{\mu }\Vert \;\Vert \varphi \Vert _\infty \Vert \nabla _1{\widehat{r}}\Psi \Vert \le C \Vert \varphi \Vert _\infty \ N^{\xi -\mu } \ln (N). \end{aligned}$$

Similarly, we obtain

$$\begin{aligned} (108)&\le 4N^2\Vert \nabla _2\Psi \Vert \; \Vert g_{\mu }\Vert \;\Vert \nabla \varphi \Vert _\infty \Vert q_2 {\widehat{m}}^a \Psi \Vert \le C \Vert \nabla \varphi \Vert _\infty \ N^{ \xi -\mu } \ln (N). \end{aligned}$$

It follows that $|\gamma _c(\Psi ,\varphi )|\le {\mathcal {K}}(\varphi , A_t) N^{ \xi -\mu } \ln (N)$.

**Control of** $$\gamma _d$$

To control $\gamma _d$ and $\gamma _e$ we will use the notation

110 $$\begin{aligned} \begin{array}{cc} m^c(k)=m^a(k)-m^a(k+1) &{}\quad m^d(k)=m^a(k)-m^a(k+2) \\ m^e(k)=m^b(k)-m^b(k+1) &{}\quad m^f(k)=m^b(k)-m^b(k+2). \\ \end{array} \end{aligned}$$

Since the second *k*-derivative of *m* is given by (see (74) for the first derivative)

$$\begin{aligned} m(k)^{\prime \prime }=\left\{ \begin{array}{ll} -1/(4\sqrt{k^3N}), &{}\quad \hbox {for }k\ge N^{1-2\xi }; \\ 0, &{}\quad \hbox {else.} \end{array} \right. \end{aligned}$$

it is easy to verify that

111 $$\begin{aligned} \Vert {\widehat{m}}_j^x\Vert _{\text {op}} \le CN ^{-2+3\xi } \text { for }x\in \{c,d,e,f\}. \end{aligned}$$

Recall that

$$\begin{aligned} \gamma _d(\Psi ,\varphi )&=2N(N-1)(N-2)\mathfrak {I}\left( \langle \!\langle \Psi ,g_{\mu }(x_{1}-x_{2}) \left[ V_N(x_1-x_3), {\widehat{r}}\right] \Psi \rangle \!\rangle \right) \\&\quad -\,N(N-1)(N-2)\mathfrak {I}\left( \langle \!\langle \Psi ,g_{\mu }(x_{1}-x_{2}) \left[ 4 \pi |\varphi |^2(x_3), {\widehat{r}}\right] \Psi \rangle \!\rangle \right) . \end{aligned}$$

Since $p_j+q_j=1$, we can rewrite ${\widehat{r}}$ as

$$\begin{aligned} {\widehat{r}}= {\widehat{m}}^bp_{1}p_{2}+{\widehat{m}}^a(p_{1}q_{2}+q_1p_2) =({\widehat{m}}^b-2{\widehat{m}}^a)p_{1}p_{2}+{\widehat{m}}^a(p_{1}+p_2). \end{aligned}$$

Thus,

112 $$\begin{aligned} |\gamma _d(\Psi ,\varphi )|&\le CN ^3\left| \langle \!\langle \Psi ,g_{\mu }(x_{1}-x_{2}) \left[ V_N(x_1-x_3), ({\widehat{m}}^b-2{\widehat{m}}^a)p_{1}p_{2}\right. \right. \nonumber \\&\quad \left. \left. +\,{\widehat{m}}^a(p_{1}+p_2)\right] \Psi \rangle \!\rangle \right| \nonumber \\&\quad +\, CN ^3\left| \langle \!\langle \Psi ,g_{\mu }(x_{1}-x_{2}) \left[ 4\pi |\varphi |^2(x_3), {\widehat{r}}\right] \Psi \rangle \!\rangle \right| \nonumber \\&\le CN ^3\left| \langle \!\langle \Psi ,g_{\mu }(x_{1}-x_{2})p_2 \left[ V_N(x_1-x_3), {\widehat{m}}^a\right] \Psi \rangle \!\rangle \right| \end{aligned}$$

113 $$\begin{aligned}&\quad +\, CN ^3\left| \langle \!\langle \Psi ,g_{\mu }(x_{1}-x_{2}) V_N(x_1-x_3) ({\widehat{m}}^b-2{\widehat{m}}^a)p_{1}p_{2}\Psi \rangle \!\rangle \right| \end{aligned}$$

114 $$\begin{aligned}&\quad +\, CN ^3\left| \langle \!\langle \Psi ,g_{\mu }(x_{1}-x_{2}) ({\widehat{m}}^b-2{\widehat{m}}^a)p_{1}p_{2}V_N(x_1-x_3) \Psi \rangle \!\rangle \right| \end{aligned}$$

115 $$\begin{aligned}&\quad +\, CN ^3\left| \langle \!\langle \Psi ,g_{\mu }(x_{1}-x_{2}) {\widehat{m}}^ap_{1} V_N(x_1-x_3)\Psi \rangle \!\rangle \right| \end{aligned}$$

116 $$\begin{aligned}&\quad +\, CN ^3\left| \langle \!\langle \Psi ,g_{\mu }(x_{1}-x_{2})V_N(x_1-x_3) {\widehat{m}}^ap_{1} \Psi \rangle \!\rangle \right| \end{aligned}$$

117 $$\begin{aligned}&\quad +\, CN ^3\left| \langle \!\langle \Psi ,g_{\mu }(x_{1}-x_{2}) \left[ 4 \pi |\varphi |^2(x_3), {\widehat{r}}\right] \Psi \rangle \!\rangle \right| . \end{aligned}$$

Using Lemma 4.2 (d), we obtain the following estimate:

$$\begin{aligned} (112)&= CN ^3\left| \langle \!\langle \Psi ,g_{\mu }(x_{1}-x_{2})p_2 \left[ V_N(x_1-x_3), p_1p_3{\widehat{m}}^d+p_1q_3{\widehat{m}}^c+q_1p_3{\widehat{m}}^c\right] \Psi \rangle \!\rangle \right| \\&\le CN ^3\big |\langle \!\langle \Psi ,V_N(x_1-x_3)g_{\mu }(x_{1}-x_{2})p_2\mathbb {1}_{\text {supp}(V_N)}(x_1-x_3) \\&\quad \left( p_1p_3{\widehat{m}}^d+p_1q_3{\widehat{m}}^c +q_1p_3{\widehat{m}}^c\right) \Psi \rangle \!\rangle \big | \\&\quad +\, CN ^3\left| \langle \!\langle \Psi ,g_{\mu }(x_{1}-x_{2})p_2\left( p_1p_3{\widehat{m}}^d+p_1q_3{\widehat{m}}^c+q_1p_3{\widehat{m}}^c\right) V_N(x_1-x_3)\Psi \rangle \!\rangle \right| . \end{aligned}$$

Both lines are bounded by

$$\begin{aligned}&CN ^3\Vert V_N(x_1-x_3)\Psi \Vert \;\Vert g_{\mu }(x_{1}-x_{2})p_2\Vert _{\text {op}} \\&\quad \times \left( 2\Vert \mathbb {1}_{\text {supp}(V_N)}(x_1-x_3)p_1\Vert _{\text {op}} +\Vert \mathbb {1}_{\text {supp}(V_N)}(x_1-x_3)p_3\Vert _{\text {op}}\right) \left( \Vert {\widehat{m}}^d\Vert _{\text {op}}+\Vert {\widehat{m}}^c\Vert _{\text {op}}\right) . \end{aligned}$$

In view of Lemmas 4.2 (e) and 5.5 (i), $\Vert g_{\mu }(x_{1}-x_{2})p_2\Vert _{\text {op}} \le \Vert \varphi \Vert _\infty \Vert g_\mu \Vert \le C \Vert \varphi \Vert _\infty N^{-1-\mu } \ln (N)$. Using (111), together with $\Vert \mathbb {1}_{\text {supp}(V_N)}(x_1-x_3)p_1\Vert _{\text {op}} \Vert V_N(x_1-x_3)\Psi \Vert \le {\mathcal {K}}(\varphi , A_t) N^{-1/2}$, we obtain, using $\xi <1/2$,

$$\begin{aligned} (112)\le {\mathcal {K}}(\varphi ,A_t) N^{-1/2+3\xi -\mu }\ln (N) \le {\mathcal {K}}(\varphi ,A_t) N^{1/2+\xi -\mu }\ln (N). \end{aligned}$$

We continue with

$$\begin{aligned} (113)+(114)+(115)&\le C N^3\Vert V_N(x_1-x_3)\Psi \Vert \Vert g_{\mu }(x_{1}-x_{2})p_2\Vert _{\text {op}} \\&\quad \times \,\Vert \mathbb {1}_{\text {supp}(V_N)}(x_1-x_3)p_1\Vert _{\text {op}}\Vert ({\widehat{m}}^b-2{\widehat{m}}^a)\Vert _{\text {op}} \\&\quad +\, CN ^3\Vert g_{\mu }(x_{1}-x_{2})p_2\Vert _{\text {op}} \Vert {\widehat{m}}^b-2{\widehat{m}}^a\Vert _{\text {op}}\Vert p_{1}V_N(x_1-x_3) \Psi \Vert \\&\quad +\, CN ^3\Vert g_{\mu }(x_{1}-x_{2})p_1\Vert _{\text {op}}\Vert {\widehat{m}}^a\Vert _{\text {op}}\Vert p_{1} V_N(x_1-x_3)\Psi \Vert \\&\le {\mathcal {K}}(\varphi ,A_t) N^{1/2+\xi -\mu } \ln (N). \end{aligned}$$

Next, we estimate (116). The support of the function $g_{\mu }(x_{1}-x_{2})V_N(x_1-x_3)$ is such that $|x_1-x_2| \le CN ^ {-\mu }$, as well as $|x_1-x_3| \le C e^ {-N}$. Therefore, $g_{\mu }(x_{1}-x_{2})V_N(x_1-x_3) \ne 0$ implies $|x_2-x_3| \le C N^ {- \mu }$. We estimate

$$\begin{aligned} {}(116)&= CN ^3\left| \langle \!\langle \Psi ,g_{\mu }(x_{1}-x_{2})V_N(x_1-x_3)p_{1} \mathbb {1}_{ B_{ CN ^{- \mu }}(0)} (x_2-x_3) {\widehat{m}}^a \Psi \rangle \!\rangle \right| \\&\le CN ^3 \Vert p_{1} V_N(x_1-x_3) g_{\mu }(x_{1}-x_{2}) \Psi \Vert \Vert \mathbb {1}_{ B_{ CN ^{- \mu }}(0)} (x_2-x_3) {\widehat{m}}^a \Psi \Vert \\&\le CN ^3 \Vert p_1 \mathbb {1}_{\text {supp}(V_N)} (x_1-x_3)\Vert _{\text {op}} \Vert g_{\mu }(x_{1} -\,x_{2}) V_N(x_1-x_3) \Psi \Vert \\&\quad \times \Vert \mathbb {1}_{ B_{ CN ^{- \mu }}(0)} (x_2-x_3) {\widehat{m}}^a \Psi \Vert \\&\le C_p N^{5/2} \Vert g_{\mu }\Vert _\infty \Vert \mathbb {1}_{ B_{ CN ^{- \mu }}(0)} \Vert ^{\frac{1}{2}}_{\frac{p}{p-1}} \Vert \nabla _1 {\widehat{m}}^a \Psi \Vert ^{ \frac{p-1}{ p}} \Vert {\widehat{m}}^a \Psi \Vert ^{\frac{1}{p}} \\&\le CN ^{5/2} \Vert g_{\mu }\Vert _\infty N^{- \mu /2} \Vert \nabla _1 {\widehat{m}}^a \Psi \Vert ^{1/2} \Vert {\widehat{m}}^a \Psi \Vert ^{1/2} \\&\le CN ^{3/2+\xi - \mu /2}. \end{aligned}$$

In the fourth line, we applied Sobolev inequality as in the proof of Lemma 7.4, then setting $p=2$. Furthermore, we used $\Vert \nabla _1 {\widehat{m}}^a \Psi \Vert ^{1/2} \Vert {\widehat{m}}^a \Psi \Vert ^{1/2} \le CN ^{-1 + \xi }$, as well as $\Vert g_{\mu }\Vert _\infty \le C$, see Lemma 5.5.

Using Lemma 4.2 (d), (117) can be bounded by

$$\begin{aligned}&CN ^3\left| \langle \!\langle \Psi ,g_{\mu }(x_{1}-x_{2}) \left[ 4 \pi |\varphi |^2(x_3), p_1p_2({\widehat{r}}-{\widehat{r}}_2)+(p_1q_2+q_1p_2)({\widehat{r}} -{\widehat{r}}_1)\right] \Psi \rangle \!\rangle \right| \\&\quad \le CN ^3\Vert \varphi \Vert _\infty ^2 \left( \Vert {\widehat{r}}-{\widehat{r}}_2\Vert _{\text {op}}+\Vert {\widehat{r}} -{\widehat{r}}_1\Vert _{\text {op}}\right) \Vert g_{\mu }(x_{1}-x_{2})p_2\Vert _{\text {op}}. \end{aligned}$$

Note that $\Vert {\widehat{r}}-{\widehat{r}}_2\Vert _{\text {op}}+\Vert {\widehat{r}}-{\widehat{r}}_1\Vert _{\text {op}}\le \sum _{j\in \{c,d,e,f\}}\Vert {\widehat{m}}^j\Vert _{\text {op}} \le CN ^{-2+ 3 \xi }$ holds. With $\Vert g_{\mu }(x_{1}-x_{2})p_2\Vert _{\text {op}}\le CN ^{-1-\mu } \ln (N)$, it then follows that

$$\begin{aligned} |(117)|\le C \Vert \varphi \Vert _\infty ^2 N^{3\xi -\mu } \ln (N). \end{aligned}$$

In total, we obtain

$$\begin{aligned} |\gamma _d (\Psi ,\varphi )| \le {\mathcal {K}}(\varphi ,A_t) \left( N^{3/2+\xi -\mu /2}+N^{1/2+3\xi -\mu }\ln (N) \right) . \end{aligned}$$

**Control of**$$\gamma _e$$

Recall that

$$\begin{aligned}&\gamma _e(\Psi ,\varphi )=-\frac{1}{2}N(N-1)(N-2)(N-3) \mathfrak {I}\left( \langle \!\langle \Psi , g_{\mu }(x_{1}-x_{2})\left[ V_N(x_3-x_4), {\widehat{r}}\right] \Psi \rangle \!\rangle \right) . \end{aligned}$$

Using symmetry, Lemma 4.2 (d) and notation (110), $\gamma _e$ is bounded by

$$\begin{aligned} \gamma _e(\Psi ,\varphi )&\le N^4\big |\langle \!\langle \Psi , g_{\mu }(x_{1}-x_{2})\big [V_N(x_3-x_4), {\widehat{m}}^cp_1p_2p_3p_4+2{\widehat{m}}^dp_1p_2p_3q_4\\&\quad +\,2{\widehat{m}}^ep_1q_2p_3p_4+4{\widehat{m}}^fp_1q_2p_3q_4\big ] \Psi \rangle \!\rangle \big | \\&\le 4N^4\Vert V_N(x_3-x_4)\Psi \Vert \Vert \mathbb {1}_{\text {supp}(V_N)} (x_3-x_4)p_3\Vert _{\text {op}}\Vert g_{\mu }(x_{1}-x_{2})p_1\Vert _{\text {op}} \\&\quad \times (\Vert {\widehat{m}}^c\Vert _{\text {op}}+\Vert {\widehat{m}}^d\Vert _{\text {op}} +\Vert {\widehat{m}}^e\Vert _{\text {op}}+\Vert {\widehat{m}}^f\Vert _{\text {op}}). \end{aligned}$$

We get with (111), Lemma 5.5 and Lemma 4.2 that

$$\begin{aligned} |\gamma _e(\Psi ,\varphi )|\le {\mathcal {K}}(\varphi ,A_t) N^{1/2+3\xi - \mu } \ln (N). \end{aligned}$$

**Control of**$$\gamma _f$$

Recall that

$$\begin{aligned} \gamma _f(\Psi ,\varphi ) = 2 N(N-1)\frac{N-2}{N-1}\mathfrak {I}\left( \langle \!\langle \Psi ,g_{\mu }(x_{1}-x_{2}) \left[ 4 \pi |\varphi |^2(x_1), {\widehat{r}}\right] \Psi \rangle \!\rangle \right) . \end{aligned}$$

We obtain the estimate

$$\begin{aligned} |\gamma _f(\Psi ,\varphi )| \le {\mathcal {K}}(\varphi ,A_t) N^2 \Vert g_\mu \Vert \Vert {\widehat{r}} \Vert _{\text {op}} \le {\mathcal {K}}(\varphi ,A_t) N^{\xi -\mu } \ln (N). \end{aligned}$$

#### Proof of Lemma 6.13

Let the assumptions of Lemma 6.13 be satisfied. With the previous estimates and $\xi <1/3$ we get

$$\begin{aligned}&\sum _{k \in \{a, b, c, d, e, f \}} \left| \gamma _k(\Psi _t,\varphi _t) \right| \le {\mathcal {K}}(\varphi _t, A_t) \Big ( \alpha (\Psi _t, \varphi _t) + N^{- \xi } + \left( N^{2 - \mu /2} + N^{-1/6} \right) \ln (N) \\&\quad +\, \inf _{ \min \{\beta , 1/2 \}>\beta _1>0} \, \inf _{\eta >0} \left( N^{ \eta - 2 \beta _1} \ln (N)^2 + N^{-1+2 \beta _1 + \xi } + N^{ 2 \xi - \eta } \right) \Big ). \end{aligned}$$

Choosing $\xi = 1/10$, $\mu =10$, $\eta = 3/10$ and $\beta _1= 3/10$, we obtain (64). $\quad \square$

### Energy estimates

In this section we show that $\Vert \mathbb {1}_{{\mathcal {A}}^{(d)}_{1}}\nabla _1q_1 \Psi \Vert ^2$ can be controlled sufficiently well in terms of the counting functionals $\alpha ^<$ and $\alpha$. If $\Psi _t$ is evolving according to $W_{\beta }$, one could actually show that $\Vert \nabla _1q_1\Psi _t \Vert ^2$ is small already without cutoff. While such a proof would be less involved, we chose a unified presentation which both covers the Gross–Pitaevskii scaling and the NLS scaling.

#### Lemma 7.9

Let $W_\beta \in {\mathcal {W}}_\beta$, $V_N \in {\mathcal {V}}_N$ and $A_t \in L^{\infty }({\mathbb {R}}^2, {\mathbb {R}})$. Let $\Psi \in L^2_s( {\mathbb {R}}^{2N}, {\mathbb {C}}) \cap H^1( {\mathbb {R}}^{2N}, {\mathbb {C}})$, $\Vert \Psi \Vert =1$ with $\Vert \nabla _1\Psi \Vert \le C$. Let $\varphi \in H^3({\mathbb {R}}^2,{\mathbb {C}}), \; \Vert \varphi \Vert =1$. For $d\ge 3$, define the sets ${\mathcal {A}}^{(d)}_{1},\overline{{\mathcal {B}}}^{(d)}_{1}$ as in Definition 7.3. Then, for *N* large enough and $d \ge 3$,

$$\begin{aligned} \Vert \mathbb {1}_{{\mathcal {A}}^{(d)}_{1}}\nabla _1q_1 \Psi \Vert ^2&\le {\mathcal {K}}(\varphi , A_t) \Big ( \langle \!\langle \Psi ,{\widehat{n}}^{\varphi }\Psi \rangle \!\rangle +N^{-1/6} \ln (N) \\&\quad +\, \inf \left\{ \left| {\mathcal {E}}_{V_N}(\Psi )-{\mathcal {E}}_{4 \pi }^{GP}(\varphi )\right| , \left| {\mathcal {E}}_{W_\beta }(\Psi )- {\mathcal {E}}_{b_{W_{\beta }}}^{GP}(\varphi ) \right| + N^{-2 \beta } \ln (N) \right\} \Big ). \end{aligned}$$

#### Proof

We start with expanding ${\mathcal {E}}_{W_\beta }(\Psi )-{\mathcal {E}}_{N \Vert W_\beta \Vert _1}^{GP}(\varphi )$. This yields

$$\begin{aligned} {\mathcal {E}}_{W_\beta }(\Psi )-{\mathcal {E}}_{N \Vert W_\beta \Vert _1}^{GP}(\varphi )&= \Vert \nabla _1\Psi \Vert ^2+\frac{N-1}{2}\Vert \sqrt{W_\beta }(x_1-x_2)\Psi \Vert ^2 \\&-\Vert \nabla \varphi \Vert ^2-\frac{1}{2} N\Vert W_\beta \Vert _1 \Vert \varphi ^2\Vert ^2+ \langle \!\langle \Psi , A_t (x_1) \Psi \rangle \!\rangle - \langle \varphi , A_t \varphi \rangle \\&= \Vert \mathbb {1}_{{\mathcal {A}}^{(d)}_{1}}\nabla _1q_1\Psi \Vert ^2+ M( \Psi , \varphi )+Q_\beta ( \Psi , \varphi ), \end{aligned}$$

where we have defined

118 $$\begin{aligned} M( \Psi , \varphi )&= 2 \mathfrak {R}\left( \langle \!\langle \nabla _1q_1\Psi , \mathbb {1}_{{\mathcal {A}}^{(d)}_{1}} \nabla _1p_1\Psi \rangle \!\rangle \right) \end{aligned}$$

119 $$\begin{aligned}&\quad +\,\Vert \mathbb {1}_{{\mathcal {A}}^{(d)}_{1}}\nabla _1p_1\Psi \Vert ^2-\Vert \nabla \varphi \Vert ^2 \end{aligned}$$

120 $$\begin{aligned}&\quad +\,\langle \!\langle \Psi , A_t(x_1)\Psi \rangle \!\rangle -\langle \varphi , A_t\varphi \rangle , \nonumber \\ Q_\beta ( \Psi , \varphi )&= \Vert \mathbb {1}_{\overline{{\mathcal {A}}}^{(d)}_{1}}\nabla _1\Psi \Vert ^2 \nonumber \\&\quad +\, \frac{N-1}{2}\langle \!\langle \Psi ,(1-p_1p_2)W_{\beta }(x_1-x_2)(1-p_1p_2)\Psi \rangle \!\rangle \nonumber \\&\quad +\, \frac{N-1}{2}\langle \!\langle \Psi ,p_1p_2W_{\beta }(x_1-x_2)p_1p_2\Psi \rangle \!\rangle -\frac{1}{2}N\Vert W_\beta \Vert _1\Vert \varphi ^2\Vert ^2 \nonumber \\&\quad +\, (N-1)\mathfrak {R}\langle \!\langle \Psi ,(1-p_1p_2)W_{\beta }(x_1-x_2)p_1p_2\Psi \rangle \!\rangle . \end{aligned}$$

Notice that the first two terms in $Q_\beta ( \Psi , \varphi )$ are nonnegative. This yields to the bound

121 $$\begin{aligned} S_\beta ( \Psi , \varphi )&= (N-1)|\langle \!\langle \Psi ,(1-p_1p_2)W_{\beta }(x_1-x_2)p_1p_2\Psi \rangle \!\rangle | \end{aligned}$$

122 $$\begin{aligned}&\quad +\, \left| \frac{N-1}{2}\langle \!\langle \Psi ,p_1p_2W_{\beta }(x_1-x_2)p_1p_2\Psi \rangle \!\rangle -\frac{1}{2}N\Vert W_\beta \Vert _1\Vert \varphi ^2\Vert ^2 \right| \nonumber \\&\ge -Q_\beta ( \Psi , \varphi ). \end{aligned}$$

We therefore obtain

123 $$\begin{aligned} \Vert \mathbb {1}_{{\mathcal {A}}^{(d)}_{1}}\nabla _1q_1\Psi \Vert ^2&\le \left| {\mathcal {E}}_{W_\beta }(\Psi )-{\mathcal {E}}_{N \Vert W_\beta \Vert _1}^{GP}(\varphi ) \right| + |M( \Psi , \varphi )|+|S_\beta ( \Psi , \varphi )|. \end{aligned}$$

Thus if we use that Definition 2.1 implies the estimate

$$\begin{aligned} \left| {\mathcal {E}}_{b_{W_{\beta }}}^{GP}(\varphi ) - {\mathcal {E}}_{N \Vert W_{\beta } \Vert _1}^{GP}(\varphi ) \right|&\le \frac{1}{2} \big | b_{W_{\beta }} - N \Vert W_{\beta } \Vert _1 \big | \; \Vert \varphi ^2 \Vert ^2 \le {\mathcal {K}}(\varphi , A_t) N^{-1} \ln (N), \end{aligned}$$

we get the bound:

$$\begin{aligned} \Vert \mathbb {1}_{{\mathcal {A}}^{(d)}_{1}}\nabla _1q_1\Psi \Vert ^2&\le \left| {\mathcal {E}}_{W_\beta }(\Psi )-{\mathcal {E}}_{b_{W_{\beta }}}^{GP}(\varphi ) \right| + {\mathcal {K}}(\varphi , A_t) N^{-1} \ln (N) \\&\quad +\, |M( \Psi , \varphi )|+|S_\beta ( \Psi , \varphi )|. \end{aligned}$$

Next, we split up the energy difference ${\mathcal {E}}_{V_N}(\Psi )-{\mathcal {E}}_{4 \pi }^{GP}(\varphi )$,

$$\begin{aligned} {\mathcal {E}}_{V_N}(\Psi )-{\mathcal {E}}_{4 \pi }^{GP}(\varphi )&= \Vert \nabla _1\Psi \Vert ^2+\frac{N-1}{2}\Vert \sqrt{V_N}(x_1-x_2)\Psi \Vert ^2 -\Vert \nabla \varphi \Vert ^2 \\&\quad -\,2\pi \Vert \varphi ^2\Vert ^2+ \langle \!\langle \Psi , A_t (x_1) \Psi \rangle \!\rangle - \langle \varphi , A_t \varphi \rangle . \end{aligned}$$

In order to better estimate the terms corresponding to the two-particle interactions, we introduce, for $\nu >d$, the potential $M_{\nu } (x)$, defined in Definition 5.3. Note, that $\nu >d$ assures that that part of the interaction $M_{\nu }$ which lies within the set ${\mathcal {A}}^{(d)}_{1}$ will be negligible. We continue with

$$\begin{aligned} {\mathcal {E}}_{V_N}(\Psi )-{\mathcal {E}}_{4 \pi }^{GP}(\varphi )&= \Vert \mathbb {1}_{{\mathcal {A}}^{(d)}_{1}}\nabla _1\Psi \Vert ^2 + \Vert \mathbb {1}_{\overline{{\mathcal {A}}}^{(d)}_{1}} \nabla _1\Psi \Vert ^2 \\&\quad +\,\frac{N-1}{2}\Vert \mathbb {1}_{\overline{{\mathcal {B}}}^{(d)}_{1}} \sqrt{V_N}(x_1-x_2)\Psi \Vert ^2 \\&\quad +\, \frac{1}{2} \langle \!\langle \Psi ,\sum _{j\ne 1}\mathbb {1}_{{\mathcal {B}}^{(d)}_{1}}\left( V_N-M_{\nu }\right) (x_1-x_j)\Psi \rangle \!\rangle \\&\quad +\, \frac{1}{2} \langle \!\langle \Psi ,\sum _{j\ne 1}\mathbb {1}_{{\mathcal {B}}^{(d)}_{1}}M_{\nu }(x_1-x_j)\Psi \rangle \!\rangle -\Vert \nabla \varphi \Vert ^2-2\pi \Vert \varphi ^2\Vert ^2 \\ {}&\quad +\,\langle \!\langle \Psi , A_t(x_1)\Psi \rangle \!\rangle -\langle \varphi , A_t\varphi \rangle . \end{aligned}$$

After reordering, the identity $q_1=1-p_1$, together with the symmetry of $\Psi \in L^2_s( {\mathbb {R}}^{2N}, {\mathbb {C}})$ gives

$$\begin{aligned} {\mathcal {E}}_{V_N}(\Psi )-{\mathcal {E}}_{4 \pi }^{GP}(\varphi )&= \Vert \mathbb {1}_{{\mathcal {A}}^{(d)}_{1}}\nabla _1q_1\Psi \Vert ^2+ \Vert \mathbb {1}_{\overline{{\mathcal {B}}}^{(d)}_{1}} \mathbb {1}_{\overline{{\mathcal {A}}}^{(d)}_{1}} \nabla _1\Psi \Vert ^2 \\&\quad +\,\frac{N-1}{2}\Vert \mathbb {1}_{\overline{{\mathcal {B}}}^{(d)}_{1}}\sqrt{V_N}(x_1-x_2)\Psi \Vert ^2 \\&\quad +\,\frac{N-1}{2}\langle \!\langle \Psi ,\mathbb {1}_{{\mathcal {B}}^{(d)}_{1}}(1-p_1p_2)M_{\nu }(x_1-x_2) (1-p_1p_2)\mathbb {1}_{{\mathcal {B}}^{(d)}_{1}}\Psi \rangle \!\rangle \\&\quad +\,\Vert \mathbb {1}_{{\mathcal {B}}^{(d)}_{1}} \mathbb {1}_{\overline{{\mathcal {A}}_1}^{(d)}}\nabla _1\Psi \Vert ^2 + \frac{1}{2} \langle \!\langle \Psi ,\sum _{j\ne 1}\mathbb {1}_{{\mathcal {B}}^{(d)}_{1}}\left( V_N-M_{\nu }\right) (x_1-x_j)\Psi \rangle \!\rangle \\&\quad +\,\frac{N-1}{2}\langle \!\langle \Psi ,\mathbb {1}_{{\mathcal {B}}^{(d)}_{1}}p_1p_2M_{\nu } (x_1-x_2)p_1p_2\mathbb {1}_{{\mathcal {B}}^{(d)}_{1}}\Psi \rangle \!\rangle -2\pi \Vert \varphi ^2\Vert ^2 \\&\quad +\,2 \mathfrak {R}\left( \langle \!\langle \nabla _1q_1\Psi , \mathbb {1}_{{\mathcal {A}}^{(d)}_{1}} \nabla _1p_1\Psi \rangle \!\rangle \right) \\&\quad +\,(N-1)\mathfrak {R}\langle \!\langle \Psi ,\mathbb {1}_{{\mathcal {B}}^{(d)}_{1}}(1-p_1p_2)M_{\nu }(x_1-x_2) p_1p_2\mathbb {1}_{{\mathcal {B}}^{(d)}_{1}}\Psi \rangle \!\rangle \\&\quad +\,\Vert \mathbb {1}_{{\mathcal {A}}^{(d)}_{1}}\nabla _1p_1\Psi \Vert ^2-\Vert \nabla \varphi \Vert ^2 \\&\quad +\,\langle \!\langle \Psi , A_t(x_1)\Psi \rangle \!\rangle -\langle \varphi , A_t\varphi \rangle \\&= \Vert \mathbb {1}_{{\mathcal {A}}^{(d)}_{1}}\nabla _1q_1\Psi \Vert ^2 + M( \Psi , \varphi )+{\tilde{Q}}_\nu ( \Psi , \varphi ). \end{aligned}$$

with

124 $$\begin{aligned} {\tilde{Q}}_\nu ( \Psi , \varphi )&= \Vert \mathbb {1}_{\overline{{\mathcal {B}}}^{(d)}_{1}} \mathbb {1}_{\overline{{\mathcal {A}}}^{(d)}_{1}} \nabla _1\Psi \Vert ^2 \nonumber \\&\quad +\, \frac{N-1}{2}\langle \!\langle \Psi ,\mathbb {1}_{{\mathcal {B}}^{(d)}_{1}}(1-p_1p_2)M_{\nu } (x_1-x_2)(1-p_1p_2)\mathbb {1}_{{\mathcal {B}}^{(d)}_{1}}\Psi \rangle \!\rangle \nonumber \\&\quad +\, \frac{N-1}{2}\Vert \mathbb {1}_{\overline{{\mathcal {B}}}^{(d)}_{1}}\sqrt{V_N}(x_1-x_2)\Psi \Vert ^2 \nonumber \\&\quad +\, \Vert \mathbb {1}_{{\mathcal {B}}^{(d)}_{1}}\mathbb {1}_{\overline{{\mathcal {A}}}^{(d)}_{1}}\nabla _1\Psi \Vert ^2 + \frac{1}{2} \langle \!\langle \Psi ,\sum _{j\ne 1}\mathbb {1}_{{\mathcal {B}}^{(d)}_{1}}\left( V_N-M_{\nu }\right) (x_1-x_j)\Psi \rangle \!\rangle \nonumber \\&\quad +\,(N-1)\mathfrak {R}\langle \!\langle \Psi ,\mathbb {1}_{{\mathcal {B}}^{(d)}_{1}}(1-p_1p_2)M_{\nu }(x_1-x_2) p_1p_2\mathbb {1}_{{\mathcal {B}}^{(d)}_{1}}\Psi \rangle \!\rangle \nonumber \\&\quad +\,\frac{N-1}{2}\langle \!\langle \Psi ,\mathbb {1}_{{\mathcal {B}}^{(d)}_{1}}p_1p_2M_{\nu }(x_1-x_2)p_ 1p_2\mathbb {1}_{{\mathcal {B}}^{(d)}_{1}}\Psi \rangle \!\rangle -2\pi \Vert \varphi ^2\Vert ^2. \end{aligned}$$

The first three terms in ${\tilde{Q}}_\nu ( \Psi , \varphi )$ are nonnegative. For $\nu >d$ and *N* large enough, Lemma 7.10 implies that (124) is also nonnegative. Thus, for $\nu >d$, we obtain the bound

125 $$\begin{aligned} {\tilde{S}}_\nu ( \Psi , \varphi )&= (N-1)\left| \langle \!\langle \Psi ,\mathbb {1}_{{\mathcal {B}}^{(d)}_{1}}(1-p_1p_2)M_{\nu }(x_1-x_2) p_1p_2\mathbb {1}_{{\mathcal {B}}^{(d)}_{1}}\Psi \rangle \!\rangle \right| \end{aligned}$$

126 $$\begin{aligned}&\quad +\,\left| \frac{N-1}{2}\langle \!\langle \Psi ,\mathbb {1}_{{\mathcal {B}}^{(d)}_{1}}p_1p_2M_{\nu } (x_1-x_2)p_1p_2\mathbb {1}_{{\mathcal {B}}^{(d)}_{1}}\Psi \rangle \!\rangle -2\pi \Vert \varphi ^2\Vert ^2 \right| \nonumber \\&\ge -{\tilde{Q}}_\nu (\Psi , \varphi ). \end{aligned}$$

In total, we obtain

127 $$\begin{aligned} \Vert \mathbb {1}_{{\mathcal {A}}^{(d)}_{1}}\nabla _1q_1\Psi \Vert ^2 \le |M( \Psi , \varphi )|+{\tilde{S}}_\nu ( \Psi , \varphi )+ \left| {\mathcal {E}}_{V_N}(\Psi )-{\mathcal {E}}_{4 \pi }^{GP}(\varphi )\right| . \end{aligned}$$

It is therefore left to estimate $M( \Psi , \varphi ),S_\beta ( \Psi , \varphi )$ and ${\tilde{S}}_\nu ( \Psi , \varphi )$.

**Estimate of** $S_\beta ( \Psi , \varphi )$ **and** ${\tilde{S}}_\nu ( \Psi , \varphi )$ **.**

The contributions (121) and (125) are estimated in Lemma 7.11.

$$\begin{aligned} {}(121), (125) \le {\mathcal {K}}(\varphi ,A_t)( \langle \!\langle \Psi , {\widehat{n}} \Psi \rangle \!\rangle + N^{-1/6} \ln (N) ). \end{aligned}$$

We are thus left to estimate (122) and (126). We begin with the estimate for (126). As in (80), we can write

$$\begin{aligned} \langle \!\langle \Psi ,\mathbb {1}_{{\mathcal {B}}^{(d)}_{1}}p_1p_2M_{\nu }(x_1-x_2)p_1p_2 \mathbb {1}_{{\mathcal {B}}^{(d)}_{1}}\Psi \rangle \!\rangle = \langle \varphi , M_{\nu } *|\varphi |^2\varphi \rangle \langle \!\langle \Psi ,\mathbb {1}_{{\mathcal {B}}^{(d)}_{1}}p_1p_2 \mathbb {1}_{{\mathcal {B}}^{(d)}_{1}}\Psi \rangle \!\rangle . \end{aligned}$$

With Lemma 7.3 (c) with $\epsilon = 1/2$, we get $\Vert \mathbb {1}_{\overline{{\mathcal {B}}}^{(d)}_{1}} \Psi \Vert \le C N^{3/2-d}$. Together with $\Vert p_1p_2\Psi \Vert ^2= 1+ 2 \Vert p_1 q_2\Psi \Vert ^2+ \Vert q_1 q_2\Psi \Vert ^2$, we therefore obtain

$$\begin{aligned} {}(126)&\le 3\Vert q_1 \Psi \Vert ^2 + C \left( N^{3/2-d} + N^{3-2d} \right) + \frac{1}{2} |N\langle \varphi , M_{\nu } *|\varphi |^2\varphi \rangle - N \Vert M_\nu \Vert _1 \Vert \varphi ^2 \Vert ^2 | \\&\quad +\, \frac{1}{2} | 4 \pi - N \Vert M_\nu \Vert _1 |\Vert \varphi ^2 \Vert ^2 + \frac{1}{2} \langle \varphi , M_{\nu } *|\varphi |^2\varphi \rangle . \end{aligned}$$

Note that, using Young’s inequality and (80)

$$\begin{aligned}&| \langle \varphi , N M_{\nu } *|\varphi |^2\varphi \rangle - N \Vert M_\nu \Vert _1\Vert \varphi ^2 \Vert ^2| \\&\quad = \left| \int _{{\mathbb {R}}^2} d^2 x |\varphi (x)|^2 \left( N( M_{\nu } *|\varphi |^2)(x) -N \Vert M_\nu \Vert _1|\varphi (x)|^2 \right) \right| \\&\quad \le \Vert \varphi \Vert _\infty ^2 \Vert N(M_\nu *|\varphi |^2)-\Vert NM_\nu \Vert _1|\varphi |^2\Vert _1 \\&\quad \le C \Vert \varphi \Vert _\infty ^2 \Vert \Delta |\varphi |^2\Vert _1 N^{-2\nu } \ln (N) \\&\quad \le {\mathcal {K}}(\varphi , A_t) N^{-2\nu } \ln (N). \end{aligned}$$

Since $|N \Vert M_{\nu }\Vert _1- 4 \pi | \le C \frac{\ln (N)}{N}$ (see Lemma 5.5) and $\langle \varphi , M_{\nu } *|\varphi |^2\varphi \rangle \le \Vert \varphi \Vert _\infty ^4 \Vert M_\nu \Vert _1 \le C \Vert \varphi \Vert _\infty ^4 N^{-1}$, it follows that

128 $$\begin{aligned} \left| (126)\right|&\le {\mathcal {K}}(\varphi ,A_t) \left( \langle \!\langle \Psi ,{\widehat{n}}^{\varphi }\Psi \rangle \!\rangle + N^{3/2-d}+N^{3-2d+2}+ N^{-2\nu } \ln (N) + N^{-1} \ln (N) \right) \nonumber \\&\le {\mathcal {K}}(\varphi ,A_t) \left( \langle \!\langle \Psi ,{\widehat{n}}^{\varphi }\Psi \rangle \!\rangle + N^{-1} \ln (N) \right) , \end{aligned}$$

where $\nu >d \ge 3$ was used in the last inequality.

Using the same estimates, we obtain

$$\begin{aligned} {}(122) \le {\mathcal {K}}(\varphi ,A_t) \left( \langle \!\langle \Psi ,{\widehat{n}}^{\varphi }\Psi \rangle \!\rangle + N^{-2\beta } \ln (N) + N^{-1} \ln (N) \right) . \end{aligned}$$

In total, we obtain, for any $\nu > d \ge 1$, the bound

$$\begin{aligned} S_\beta (\Psi ,\varphi )&\le {\mathcal {K}}(\varphi ,A_t) \left( \langle \!\langle \Psi ,{\widehat{n}}\Psi \rangle \!\rangle +N^{-2\beta } \ln (N) +N^{-1/6 } \ln (N) \right) \\ {\tilde{S}}_\nu (\Psi ,\varphi )&\le {\mathcal {K}}(\varphi ,A_t)\left( \langle \!\langle \Psi ,{\widehat{n}}\Psi \rangle \!\rangle +N^{-1/6} \ln (N) \right) . \end{aligned}$$

**Estimate of**$M(\Psi , \varphi )$. We need to estimate (118), (119) and (120). We start with

$$\begin{aligned} |(118)|&\le 2|\langle \!\langle \nabla _1q_1\Psi , \mathbb {1}_{\overline{{\mathcal {A}}}^{(d)}_{1}} \nabla _1p_1\Psi \rangle \!\rangle |+2|\langle \!\langle \nabla _1q_1\Psi , \nabla _1p_1\Psi \rangle \!\rangle | \\&\le 2\Vert \nabla _1q_1\Psi \Vert \;\Vert \mathbb {1}_{\overline{{\mathcal {A}}}^{(d)}_{1}}\nabla _1p_1\Vert _{\text {op}} +2|\langle \!\langle {\widehat{n}}^{-1/2}q_1\Psi , \Delta _1 p_1{\widehat{n}}_{1}^{1/2}\Psi \rangle \!\rangle |. \end{aligned}$$

By Lemma 7.4, we obtain $\Vert \mathbb {1}_{\overline{{\mathcal {A}}}^{(d)}_{1}}\nabla _1p_1\Vert _{\text {op}} \le C \Vert \nabla \varphi \Vert _\infty N^{1/2-d}$.

Furthermore, we use $\Vert \nabla _1 q_1\Psi \Vert \le \Vert \nabla _1 \Psi \Vert +\Vert \nabla _1 p_1\Psi \Vert \le {\mathcal {K}}(\varphi , A_t)$ (see also Lemma 7.6) and $|\langle \!\langle {\widehat{n}}^{-1/2}q_1\Psi , \Delta _1 p_1{\widehat{n}}_{1}^{1/2}\Psi \rangle \!\rangle | \le {\mathcal {K}}(\varphi , A_t) \Vert {\widehat{n}}_{1}^{1/2}\Psi \Vert \Vert {\widehat{n}}^{1/2}\Psi \Vert \le {\mathcal {K}}(\varphi , A_t) ( \langle \!\langle \Psi ,{\widehat{n}}\Psi \rangle \!\rangle + N^{-1} )$. Hence, for $d\ge 3$,

$$\begin{aligned} |(118)|&\le {\mathcal {K}}(\varphi , A_t) (\langle \!\langle \Psi ,{\widehat{n}}\Psi \rangle \!\rangle +N^{1 -d} + N^{-1}) \le {\mathcal {K}}(\varphi ,A_t) (\langle \!\langle \Psi ,{\widehat{n}}\Psi \rangle \!\rangle + N^{-1}). \end{aligned}$$

With $\Vert \nabla _1 p_1 \Psi \Vert ^2= \Vert \nabla \varphi \Vert ^2 \Vert p_1 \Psi \Vert ^2$ line (119) is estimated by

$$\begin{aligned} {}(119)&= \Vert \mathbb {1}_{{\mathcal {A}}^{(d)}_{1}}\nabla _1p_1\Psi \Vert ^2- \Vert \nabla \varphi \Vert ^2 \\&\le | \Vert \nabla _1 p_1 \Psi \Vert ^2- \Vert \nabla \varphi \Vert ^2 | + \Vert \mathbb {1}_{\overline{{\mathcal {A}}}^{(d)}_{1}}\nabla _1p_1\Psi \Vert ^2 \\&\le C \left( \Vert \nabla \varphi \Vert ^2 \langle \!\langle \Psi , q_1\Psi \rangle \!\rangle + \Vert \nabla \varphi \Vert _\infty ^2 N^{1 - 2d} \right) \\&\le {\mathcal {K}}(\varphi ,A_t) \left( \langle \!\langle \Psi , {\widehat{n}} \Psi \rangle \!\rangle + N^{-1} \right) . \end{aligned}$$

For line (120), we use Lemma 7.5 to obtain

$$\begin{aligned} {} (120)\le C\Vert A_t\Vert _{\infty } \left( \langle \!\langle \Psi , {\widehat{n}} \Psi \rangle \!\rangle + N^{-1/2} \right) . \end{aligned}$$

In total, we obtain

$$\begin{aligned} M(\Psi , \varphi ) \le {\mathcal {K}}(\varphi ,A_t) \left( \langle \!\langle \Psi , {\widehat{n}} \Psi \rangle \!\rangle + N^{-1/2} \right) . \end{aligned}$$

$$\square $$

#### Lemma 7.10

- Let $V_N \in {\mathcal {V}}_N$ and let $R_\nu$ and $M_\nu$ be defined as in Definition 5.3. Then, for any $\Psi \in L^2({\mathbb {R}}^{2N}, {\mathbb {C}}) \cap {\mathcal {D}}(\nabla _1)$
  129 $$\begin{aligned} \Vert \mathbb {1}_{|x_1-x_2|\le R_{\nu }}\nabla _1\Psi \Vert ^2+\frac{1}{2}\langle \!\langle \Psi , (V_{N}-M_{\nu })(x_1-x_2)\Psi \rangle \!\rangle \ge 0. \end{aligned}$$
- Let $V_N \in {\mathcal {V}}_N$ and let $M_\nu$ be defined as in Definition 5.3. Let $\Psi \in L_s^2({\mathbb {R}}^{2N}, {\mathbb {C}}) \cap H^1({\mathbb {R}}^{2N}, {\mathbb {C}})$. Then, for sufficiently large *N* and for $\nu >d$,
  130 $$\begin{aligned} \Vert \mathbb {1}_{{\mathcal {B}}^{(d)}_{1}}\mathbb {1}_{\overline{{\mathcal {A}}}^{(d)}_{1}}\nabla _1\Psi \Vert ^2 + \frac{1}{2} \langle \!\langle \Psi ,\sum _{j\ne 1}\mathbb {1}_{{\mathcal {B}}^{(d)}_{1}}\left( V_N-M_{\nu }\right) (x_1-x_j)\Psi \rangle \!\rangle \ge 0. \end{aligned}$$

#### Proof

- We first show nonnegativity of the one-particle operator $H^{Z_n} : H^2 ({\mathbb {R}}^2, {\mathbb {C}}) \rightarrow L^ 2( {\mathbb {R}}^2, {\mathbb {C}})$ given by
  $$\begin{aligned} H^{Z_n}=-\Delta + \frac{1}{2} \sum _{z_k\in Z_n} (V_{N}(\cdot -z_k)-M_{\nu }(\cdot -z_k)) \end{aligned}$$
  for any $n\in {\mathbb {N}}$ and any *n*-elemental subset $Z_n\subset {\mathbb {R}}^2$ which is such that the supports of the potentials $M_{\nu }(\cdot -z_k)$ are pairwise disjoint for any two $z_k\in Z_n$. Since $f_{\nu }(\cdot -z_k)$ is the the zero energy scattering state of the potential $1/2 V_{N}(\cdot -z_k)- 1/2 M_{\nu }(\cdot -z_k)$, it follows that
  $$\begin{aligned} F^{Z_n}_{\nu }=\prod _{z_k\in Z_n}f_{\nu }(\cdot -z_k). \end{aligned}$$
  fulfills $H^{Z_n} F^{Z_n}_{\nu }=0$ for any such $Z_n$. By construction $f_{\nu }$ is a nonnegative function, so is $F^{Z_n}_{\nu }$. Since $\frac{1}{2} \sum _{z_k\in Z_n} (V_{N}(\cdot -z_k)-M_{\nu }(\cdot -z_k)) \in L^\infty ({\mathbb {R}}^2,{\mathbb {C}})$, this potential is a infinitesimal perturbation of $-\Delta$, thus $\sigma _{\text {ess}}( H^{Z_n} ) =[0, \infty )$. Assume now that $H^{Z_n}$ is not nonnegative. Then, there exists a ground state $\Psi _G\in H^2( {\mathbb {R}}^2,{\mathbb {C}})$ of $H^{Z_n}$ of negative energy $E<0$. The phase of the ground state can be chosen such that the ground state is real and positive a.e. (see e.g. [52], Theorem 10.12.). Since $f_{\nu }(\cdot -z_k)$ is positive outside $\text {supp}(V_N)$, the following inequality is valid^6^
  131 $$\begin{aligned} \langle F^{Z_n}_{\nu },H^{Z_n}\Psi _G\rangle =\langle F^{Z_n}_{\nu },E\Psi _G\rangle <0. \end{aligned}$$
  On the other hand we have since $F^{Z_n}_{\nu }$ is the zero energy scattering state
  $$\begin{aligned} \langle F^{Z_n}_{\nu },H^{Z_n}\Psi _G\rangle =\langle H^{Z_n}F^{Z_n}_{\nu },\Psi _G\rangle =0. \end{aligned}$$
  This contradicts (131) and the nonnegativity of $H^{Z_n}$ follows. Now, assume that there exists a $\psi \in H^2 ({\mathbb {R}}^2,{\mathbb {C}})$ such that the quadratic form
  $$\begin{aligned} Q (\psi )= \Vert \mathbb {1}_{|\cdot |\le R_{\nu }}\nabla \psi \Vert ^2+ \frac{1}{2}\langle \psi ,(V_{N}(\cdot )-M_{\nu }(\cdot ))\psi \rangle <0. \end{aligned}$$
  Since $V_{N}$ and $M_{\nu }$ are spherically symmetric we can assume that $\psi$ is spherically symmetric. Substituting $\psi \rightarrow a \psi ,\; a \in {\mathbb {R}}$, we can furthermore assume that, for all $|x| = R_\nu$, $\psi (x) = 1- \epsilon$ for $\epsilon >0$. Define ${\tilde{\psi }}$ such that ${\tilde{\psi }}(x)=\psi (x) \text { for } |x| \le R_{\nu }$ and ${\tilde{\psi }}(x)=1$ for $|x|> R_{\nu }+ \epsilon$ and $\epsilon >0$. Furthermore, ${\tilde{\psi }}$ can be constructed such that $\Vert \mathbb {1}_{|\cdot | \ge R_\nu } \nabla {\tilde{\psi }}\Vert ^2 \le C (\epsilon + \epsilon ^ 2)$. Then $Q ( {\tilde{\psi }})= Q (\psi )<0$ holds, because the operator associated with the quadratic form is supported inside the ball $B_0 (R_{\nu })$. Using ${\tilde{\psi }}$, we can construct a set of points $Z_n$ and a $\chi \in H^2({\mathbb {R}}^2, {\mathbb {C}})$ such that $\langle \chi ,H^{Z_n}\chi \rangle <0$, contradicting to nonnegativity of $H^{Z_n}$. For $R>1$ let
  $$\begin{aligned} \xi _R(x)=\left\{ \begin{array}{ll} R^2/x^2, &{}\quad \hbox {for }|x|>R; \\ 1, &{}\quad \hbox {else.} \end{array} \right. \end{aligned}$$
  Let now $Z_n$ be a subset $Z_n\subset {\mathbb {R}}^2$ with $|Z_n|=n$ which is such that the supports of the potentials $M_{\nu }(\cdot -z_k)$ lie within the Ball around zero with radius *R* and are pairwise disjoint for any two $z_k\in Z_n$. Since we are in two dimensions we can choose a *n* which is of order $R^2$. Let now $\chi _R(x)=\xi _R (x) \prod _{z_k\in Z_n} {\tilde{\psi }}(x-z_k)$. By construction, there exists a $D = {\mathcal {O}}(1)$ such that $\chi _R(x)= {\tilde{\psi }}(x-z_k)$ for $|x-z_k| \le D$. From this, we obtain
  $$\begin{aligned} \langle \chi _R,H^{Z_n}\chi _R\rangle&= \Vert \nabla \chi _R \Vert ^2+ n\frac{1}{2}\langle \psi ,(V_{N}(\cdot )-M_{\nu }(\cdot ))\psi \rangle \\&= n Q(\psi )+ \sum _{z_k \in Z_n} \Vert \mathbb {1}_{|\cdot -z_k|\ge R_\nu } \nabla \chi _R \Vert ^2 \\&\le n Q(\psi ) +C n (\epsilon +\epsilon ^2) + \Vert \nabla \xi _R \Vert ^2 \\&= n Q(\psi ) +C n (\epsilon + \epsilon ^2) +C. \end{aligned}$$
  Choosing *R* and hence *n* large enough and $\epsilon$ small, we can find a $Z_n$ such that $\langle \chi _R,H^{Z_n}\chi _R\rangle$ is negative, contradicting nonnegativity of $H^{Z_n}$. Now, we can prove that
  132 $$\begin{aligned} \Vert \mathbb {1}_{|x_1-x_2|\le R_{\nu }}\nabla _1\Psi \Vert ^2+\frac{1}{2}\langle \!\langle \Psi , (V_{N}-M_{\nu })(x_1-x_2)\Psi \rangle \!\rangle \ge 0. \end{aligned}$$
  holds for any $$\Psi \in H^2({\mathbb {R}}^{2N},{\mathbb {C}})$$. Using the coordinate transformation $${\tilde{x}}_1= x_1-x_2, {\tilde{x}}_i=x_i \; \forall i \ge 2$$, we have $$\nabla _{x_1}= \nabla _{{\tilde{x}}_1}$$. Thus (132) is equivalent to $$ {\tilde{Q}}(\Psi ):= \Vert \mathbb {1}_{|x_1|\le R_{\nu }}\nabla _1\Psi \Vert ^2+\frac{1}{2}\langle \!\langle \Psi , (V_{N}-M_{\nu })(x_1)\Psi \rangle \!\rangle \ge 0$$$$\forall \Psi \in H^2({\mathbb {R}}^{2N},{\mathbb {C}})$$. If it were now that $$ {\tilde{Q}}(\Psi ) $$ is not nonnegative, then there exists a $$\Gamma \in H^2({\mathbb {R}}^{2N},{\mathbb {C}})$$ such that $$ {\tilde{Q}}(\Gamma ) <0 $$. By the Schmidt decomposition theorem, there exist two orthonormal bases $$\{ \Phi _k \}_{k \in {\mathbb {N}}} \subset H^2({\mathbb {R}}^{2N-2}, {\mathbb {C}}), \{ \varphi _l \}_{l \in {\mathbb {N}}} \subset H^2({\mathbb {R}}^{2}, {\mathbb {C}})$$ and nonnegative numbers $$\{\lambda _k\}_{k \in {\mathbb {N}}}$$ such that
  $$\begin{aligned} \Gamma = \sum _{k \in {\mathbb {N}}} \lambda _k \varphi _k \otimes \Phi _k. \end{aligned}$$
  By this
  $$\begin{aligned} {\tilde{Q}}(\Gamma ) = \sum _{k \in {\mathbb {N}}} | \lambda _k|^ 2 Q(\varphi _k) \ge 0, \end{aligned}$$
  which in turn yields to a contradictions. Therefore, $$Q(\Psi ) \ge 0$$ for all $$\Psi \in H^2({\mathbb {R}}^{2},{\mathbb {C}})$$. By a standard density argument, we can conclude that $$Q(\Psi ) \ge 0$$$$\forall \Psi \in L^2({\mathbb {R}}^{2N}, {\mathbb {C}}) \cap {\mathcal {D}}(\nabla _1)$$.
- Define $c_k= \lbrace (x_1,\dots ,x_N) \in {\mathbb {R}}^{2N}| |x_1-x_k|\le R_\nu \rbrace$ and ${\mathcal {C}}_1= \cup _{k=2}^N c_k$. For $(x_1, \dots , x_N) \in {\mathcal {B}}^{(d)}_{1}$ it holds that $|x_i-x_j| \ge N^{-d}$ for $2 \le i,j \le N$. Let $\nu >d$. Assume that $N^{-d} > 2 R_\nu$, which hold for *N* sufficiently large, since $R_\nu \le CN ^{-\nu }$. Then, it follows that, for $i \ne j$, $\left( c_i \cap {\mathcal {B}}^{(d)}_{1} \right) \cap \left( c_j \cap {\mathcal {B}}^{(d)}_{1} \right) = \emptyset$. Under the same conditions, we also have $\mathbb {1}_{\overline{{\mathcal {A}}}^{(d)}_{1}} \ge \mathbb {1}_{{\mathcal {C}}_{1}}$. Therefore
  $$\begin{aligned}&\mathbb {1}_{\overline{{\mathcal {A}}}^{(d)}_{1}} \mathbb {1}_{{\mathcal {B}}^{(d)}_{1}} \ge \mathbb {1}_{{\mathcal {C}}_{1}} \mathbb {1}_{{\mathcal {B}}^{(d)}_{1}} = \mathbb {1}_{{\mathcal {C}}_{1} \cap {\mathcal {B}}^{(d)}_{1} } = \mathbb {1}_{ \cup _{k=2}^N \left( c_k \cap {\mathcal {B}}^{(d)}_{1} \right) } = \sum _{k=2}^N \mathbb {1}_{c_k \cap {\mathcal {B}}^{(d)}_{1} } =\mathbb {1}_{ {\mathcal {B}}^{(d)}_{1} } \sum _{k=2}^N \mathbb {1}_{c_k }. \end{aligned}$$
  Note that $\mathbb {1}_{{\mathcal {B}}^{(d)}_{1}}$ depends only on $x_2, \dots , x_N$. By this
  $$\begin{aligned} \Vert \mathbb {1}_{\overline{{\mathcal {A}}}^{(d)}_{1}} \mathbb {1}_{{\mathcal {B}}^{(d)}_{1}} \nabla _1 \Psi \Vert ^2 \ge \sum _{k=2}^N \Vert \mathbb {1}_{c_k } \nabla _1 \mathbb {1}_{ {\mathcal {B}}^{(d)}_{1} } \Psi \Vert ^2 = (N-1) \Vert \mathbb {1}_{|x_1-x_2| \le R_\beta } \nabla _1 \mathbb {1}_{ {\mathcal {B}}^{(d)}_{1} } \Psi \Vert ^2. \end{aligned}$$
  This yields
  $$\begin{aligned} {}(130)&\ge (N-1) \left( \Vert \mathbb {1}_{|x_1-x_2| \le R_\nu } \nabla _1 \mathbb {1}_{ {\mathcal {B}}^{(d)}_{1} } \Psi \Vert ^2 + \frac{1}{2}\langle \!\langle \mathbb {1}_{ {\mathcal {B}}^{(d)}_{1} } \Psi , (V_{N}-M_{\nu })(x_1-x_2)\mathbb {1}_{ {\mathcal {B}}^{(d)}_{1} } \Psi \rangle \!\rangle \right) \\&\ge 0. \end{aligned}$$
  where the last inequality follows from (a), using $\mathbb {1}_{ {\mathcal {B}}^{(d)}_{1} } \Psi \in L^2({\mathbb {R}}^{2N}, {\mathbb {C}}) \cap {\mathcal {D}}(\nabla _1)$.

$$\quad \square $$

#### Lemma 7.11

Let $W_\beta \in {\mathcal {W}}_\beta$. Let $\Psi \in L^2_{s}({\mathbb {R}}^{2N}, {\mathbb {C}}) \cap H^1({\mathbb {R}}^{2N}, {\mathbb {C}})$ and $\Vert \nabla _1\Psi \Vert$ be bounded uniformly in *N*. Let *d* in Definition 7.3 of $\mathbb {1}_{{{\mathcal {B}}_1^{(d)}}}$ sufficiently large. Let $\Gamma \in \lbrace \Psi , \mathbb {1}_{{{\mathcal {B}}_1^{(d)}}} \Psi \rbrace$. Then, for all $\beta >0$,

- $$\begin{aligned}&N\left| \langle \!\langle \Gamma , q_1p_2 W_\beta (x_1-x_2) p_1p_2 \Gamma \rangle \!\rangle \right| \le C \Vert \varphi \Vert _\infty ^2 \left( \langle \!\langle \Psi , {\hat{n}} \Psi \rangle \!\rangle + N^{-1} \right) , \\&N\left| \langle \!\langle \Gamma , p_1q_2 W_\beta (x_1-x_2) p_1p_2 \Gamma \rangle \!\rangle \right| \le C \Vert \varphi \Vert _\infty ^2 \left( \langle \!\langle \Psi , {\hat{n}} \Psi \rangle \!\rangle + N^{-1} \right) . \end{aligned}$$
- $$\begin{aligned}&N|\langle \!\langle \Gamma , p_1p_2 W_\beta (x_1-x_2) q_1q_2 \Gamma \rangle \!\rangle | \le {\mathcal {K}}(\varphi ,A_t) \left( \langle \!\langle \Psi , {\widehat{n}} \Psi \rangle \!\rangle + N^{-1/6} \ln (N) \right) . \end{aligned}$$
- $$\begin{aligned}&N|\langle \!\langle \Gamma , (1-p_1p_2) W_\beta (x_1-x_2) p_1p_2 \Gamma \rangle \!\rangle | \le {\mathcal {K}}(\varphi ,A_t) \left( \langle \!\langle \Psi , {\widehat{n}} \Psi \rangle \!\rangle + N^{-1/6} \ln (N) \right) . \end{aligned}$$

#### Proof

(a) We will only consider the first inequality of (a). The second inequality of (a) can be proven analogously. Let first $\Gamma = \mathbb {1}_{{{\mathcal {B}}_1^{(d)}}} \Psi$. Then,

133 $$\begin{aligned}&N \left| \langle \!\langle \mathbb {1}_{{{\mathcal {B}}_1^{(d)}}} \Psi , q_1p_2 W_\beta (x_1-x_2) p_1p_2 \mathbb {1}_{{{\mathcal {B}}_1^{(d)}}} \Psi \rangle \!\rangle \right| \nonumber \\&\quad \le N\left| \langle \!\langle \mathbb {1}_{{\overline{{\mathcal {B}}}^{(d)}_{1}}} \Psi , q_1p_2 W_\beta (x_1-x_2) p_1p_2 \mathbb {1}_{{{\mathcal {B}}_1^{(d)}}} \Psi \rangle \!\rangle \right| \end{aligned}$$

134 $$\begin{aligned}&\qquad +\, N\left| \langle \!\langle \Psi , q_1p_2 W_\beta (x_1-x_2) p_1p_2 \mathbb {1}_{{{\mathcal {B}}_1^{(d)}}} \Psi \rangle \!\rangle \right| . \end{aligned}$$

Using Lemma 7.4 (c) with $\epsilon =1$, together with $\Vert p_2 W_\beta (x_1-x_2) p_2 \Vert _{\text {op}} \le \Vert \varphi \Vert _\infty ^2 \Vert W_\beta \Vert _1$, the first line can be bounded by

135 $$\begin{aligned}&(133) \le {\mathcal {K}}(\varphi , A_t) N \Vert \mathbb {1}_{{\overline{{\mathcal {B}}}^{(d)}_{1}}} \Psi \Vert \Vert W_{\beta } \Vert _{1} \le {\mathcal {K}}(\varphi , A_t) N^{2- d}. \end{aligned}$$

The second term is bounded by

$$\begin{aligned} {}(134)&= N\left| \langle \!\langle \sqrt{W_\beta (x_1-x_2)} q_1p_2 ({\hat{n}})^{-\frac{1}{2}} \Psi , \sqrt{W_\beta (x_1-x_2)} p_1p_2 {\hat{n}}_{1}^{\frac{1}{2}} \mathbb {1}_{{{\mathcal {B}}^{(d)}}_{1}} \Psi \rangle \!\rangle \right| \\&\le CN \Vert \sqrt{W_\beta (x_1-x_2)} p_2 \Vert _{\text {op}}^2 \left( \Vert q_1 ({\hat{n}})^{-\frac{1}{2}} \Psi \Vert ^2 + \Vert {\hat{n}}_1^{\frac{1}{2}} \mathbb {1}_{{{\mathcal {B}}^{(d)}}_{1}} \Psi \Vert ^2 \right) \\&\le CN \Vert \sqrt{W_\beta (x_1-x_2)} p_2 \Vert _{\text {op}}^2 \left( \langle \!\langle \Psi , {\hat{n}} \Psi \rangle \!\rangle + \Vert {\hat{n}}_1^{\frac{1}{2}} \Psi \Vert ^2 + \Vert {\hat{n}}_1^{\frac{1}{2}} \mathbb {1}_{{\overline{{\mathcal {B}}}^{(d)}_{1}}} \Psi \Vert ^2 \right) \\&\le CN \Vert W_\beta \Vert _1 \Vert \varphi \Vert _\infty ^2 \left( \langle \!\langle \Psi , {\hat{n}} \Psi \rangle \!\rangle + \Vert \mathbb {1}_{{\overline{{\mathcal {B}}}^{(d)}_{1}}} \Psi \Vert ^2 \right) \\&\le C \Vert \varphi \Vert _\infty ^2 \left( \langle \!\langle \Psi , {\hat{n}} \Psi \rangle \!\rangle + N^{4 -2 d } \right) \le C \Vert \varphi \Vert _\infty ^2 \left( \langle \!\langle \Psi , {\hat{n}} \Psi \rangle \!\rangle + N^{-1} \right) . \end{aligned}$$

This yields (a) in the case $\Gamma = \mathbb {1}_{{{\mathcal {B}}_1^{(d)}}} \Psi$. The inequality (a) can be proven analogously for $\Gamma = \Psi$.

(b) Let $\Gamma = \mathbb {1}_{{{\mathcal {B}}_1^{(d)}}} \Psi$. We first consider (b) for potentials with $\beta < 1/4$. We have to estimate
  136 $$\begin{aligned}&N |\langle \!\langle \mathbb {1}_{{{\mathcal {B}}_1^{(d)}}} \Psi ,p_1 p_2 W_\beta (x_1-x_2) q_1 q_2 \mathbb {1}_{{{\mathcal {B}}^{(d)}_{1}}}\Psi \rangle \!\rangle | \nonumber \\&\quad \le N|\langle \!\langle \Psi ,p_1 p_2 W_\beta (x_1-x_2) q_1 q_2 \Psi \rangle \!\rangle | \nonumber \\&\qquad +\, N|\langle \!\langle \mathbb {1}_{\overline{{\mathcal {B}}}^{(d)}_{1}}\Psi ,p_1 p_2 W_\beta (x_1-x_2) q_1 q_2 \Psi \rangle \!\rangle | \nonumber \\&\qquad +\, N|\langle \!\langle \Psi ,p_1 p_2 W_\beta (x_1-x_2) q_1 q_2 \mathbb {1}_{\overline{{\mathcal {B}}^{(d)}_{1}}}\Psi \rangle \!\rangle | \nonumber \\&\qquad +\, N|\langle \!\langle \mathbb {1}_{\overline{{\mathcal {B}}}^{(d)}_{1}}\Psi ,p_1 p_2 W_\beta (x_1-x_2) q_1 q_2 \mathbb {1}_{\overline{{\mathcal {B}}^{(d)}_{1}}}\Psi \rangle \!\rangle | \nonumber \\&\quad \le N|\langle \!\langle \Psi ,p_1 p_2 W_\beta (x_1-x_2) q_1 q_2 \Psi \rangle \!\rangle | \end{aligned}$$
  137 $$\begin{aligned}&\qquad +\, CN \Vert \mathbb {1}_{\overline{{\mathcal {B}}}^{(d)}_{1}}\Psi \Vert \Vert W_\beta \Vert _\infty . \end{aligned}$$
  The last term is bounded using Lemma 7.4 (c) with $\epsilon =1$
  $$\begin{aligned} {}(137) \le CN N^{2-d} N^{-1+2 \beta } \le N^{- 1/2}, \end{aligned}$$
  where the last inequality holds choosing $d \ge 3$. Using Lemmas 4.2 (c) and 4.6 with $O_{1,2}=q_2 W_\beta (x_1-x_2) p_2$, $\Omega =N^{-1/2}q_1\Psi$ and $\chi =N^{1/2}p_1\Psi$ we get
  $$\begin{aligned} {}(136)&\le \left\| q_1\Psi \right\| ^2+ N^2\big |\langle \!\langle q_2\,\Psi ,p_1\sqrt{W_\beta }(x_1-x_2) p_3 \sqrt{W_\beta }(x_1-x_3)\\&\quad \sqrt{W_\beta }(x_1-x_2)p_2\sqrt{W_\beta }(x_1-x_3)p_1 q_3\,\Psi \rangle \!\rangle \big | \\&\quad +\,N^2(N-1)^{-1}\Vert q_2 W_\beta (x_1-x_2) p_2p_1\Psi \Vert ^2&\\&\le \left\| q_1\Psi \right\| ^2+N^2\Vert \sqrt{W_\beta }(x_1-x_2)p_1\Vert _{\text {op}}^4\;\Vert q_2\,\Psi \Vert ^2 \\&\quad +\, CN \Vert W_\beta (x_1-x_2) p_2\Vert _{\text {op}}^2. \end{aligned}$$
  With Lemma 4.2 (e) we get the bound
  $$\begin{aligned} {}(136)&\le \Vert q_1\Psi \Vert ^2+ N^2\Vert \varphi \Vert _\infty ^4\Vert W_\beta \Vert _1^2\;\Vert q_1\Psi \Vert ^2 + CN \Vert W_\beta \Vert ^2\Vert \varphi \Vert _\infty ^2. \end{aligned}$$
  Note, that $\Vert W_\beta \Vert _1\le CN ^{-1}$, $\Vert W_\beta \Vert ^2\le CN ^{-2+2\beta }$. Hence
  $$\begin{aligned} {}(136)\le C\left( \langle \!\langle \Psi ,q_1\Psi \rangle \!\rangle + {\mathcal {K}}(\varphi , A_t) N^{-1+2\beta }\right) . \end{aligned}$$
  Note that, for $\beta <1/4$, $N^{-1+2\beta } \le N^ {-1/6} \ln (N)$. Using the same bounds for $\Gamma = \Psi$, we obtain (b) for the case $\beta <1/4$.
b) for $1/4\le \beta$: We use $U_{\beta _1,\beta }$ from Definition 7.1 for some $0<\beta _1<1/4$. By the estimate above,it is left to control
  $$\begin{aligned} N\left| \langle \!\langle \mathbb {1}_{{{\mathcal {B}}_1^{(d)}}} \Psi , p_1p_2\left( W_{\beta }(x_1-x_2)-U_{\beta _1,\beta }(x_1-x_2)\right) q_1q_2 \mathbb {1}_{{{\mathcal {B}}_1^{(d)}}} \Psi \rangle \!\rangle \right| . \end{aligned}$$
  Let $\Delta h_{\beta _1,\beta }=W_{\beta }-U_{\beta _1,\beta }$. Integrating by parts and using that $\nabla _1 h_{\beta _1,\beta }(x_1-x_2)=-\nabla _2 h_{\beta _1,\beta }(x_1-x_2)$ gives
  138 $$\begin{aligned}&N\left| \langle \!\langle \mathbb {1}_{{{\mathcal {B}}_1^{(d)}}} \Psi , p_1p_2\left( W_{\beta }(x_1-x_2)-U_{\beta _1,\beta }(x_1-x_2)\right) q_1q_2 \mathbb {1}_{{{\mathcal {B}}_1^{(d)}}} \Psi \rangle \!\rangle \right| \nonumber \\&\quad =N\left| \langle \!\langle \nabla _1p_1 \mathbb {1}_{{{\mathcal {B}}_1^{(d)}}} \Psi , p_2\nabla _2 h_{\beta _1,\beta }(x_1-x_2)q_1q_2 \mathbb {1}_{{{\mathcal {B}}_1^{(d)}}} \Psi \rangle \!\rangle \right| \end{aligned}$$
  139 $$\begin{aligned}&\qquad +\,N\left| \langle \!\langle \mathbb {1}_{{{\mathcal {B}}_1^{(d)}}} \Psi , p_1p_2\nabla _2 h_{\beta _1,\beta }(x_1-x_2)\nabla _1q_1q_2 \mathbb {1}_{{{\mathcal {B}}_1^{(d)}}} \Psi \rangle \!\rangle \right| . \end{aligned}$$
  Let $(a_1,b_1)=(q_1,\nabla p_1)$ or $(a_1,b_1)=( \nabla q_1,p_1)$. Then, both terms can be estimated as follows: We use Lemma 4.6 with $\Omega =N^{-\eta /2} a_1\mathbb {1}_{{{\mathcal {B}}_1^{(d)}}} \Psi$, $O_{1,2}=N^{1+\eta /2}q_2\nabla _2 h_{\beta _1,\beta }(x_1-x_2)p_2$ and $\chi = b_1 \mathbb {1}_{{{\mathcal {B}}_1^{(d)}}} \Psi$. We choose $\eta < 2 \beta _1$.
  140 $$\begin{aligned}&N \left| \langle \!\langle \mathbb {1}_{{{\mathcal {B}}_1^{(d)}}} \Psi , a_1p_2\nabla _2 h_{\beta _1,\beta }(x_1-x_2)b_1q_2 \mathbb {1}_{{{\mathcal {B}}_1^{(d)}}} \Psi \rangle \!\rangle \right| \nonumber \\&\quad \le N^{-\eta }\Vert a_1 \mathbb {1}_{{{\mathcal {B}}_1^{(d)}}} \Psi \Vert ^2 \end{aligned}$$
  141 $$\begin{aligned}&\qquad +\, \frac{N^{2+\eta }}{N-1}\Vert q_2\nabla _2 h_{\beta _1,\beta }(x_1-x_2)b_1 p_2 \mathbb {1}_{{{\mathcal {B}}_1^{(d)}}} \Psi \Vert ^2 \end{aligned}$$
  142 $$\begin{aligned}&\qquad +\,N^{2+\eta }\left| \langle \!\langle \mathbb {1}_{{{\mathcal {B}}_1^{(d)}}} \Psi , b_1 p_2 q_3\nabla _2 h_{\beta _1,\beta }(x_1-x_2)\nabla _3 h_{\beta _1,\beta } (x_1-x_3)b_1 q_2p_3\mathbb {1}_{{{\mathcal {B}}_1^{(d)}}} \Psi \rangle \!\rangle \right| ^{1/2}. \end{aligned}$$
  We obtain (note that $\mathbb {1}_{{{\mathcal {B}}_1^{(d)}}}$ does not depend on $x_1$)
  $$\begin{aligned} (140)\le N^{-\eta }\Vert a_1 \mathbb {1}_{{{\mathcal {B}}_1^{(d)}}} \Psi \Vert ^2 = N^{-\eta }\Vert \mathbb {1}_{{{\mathcal {B}}_1^{(d)}}} a_1\Psi \Vert ^2 \le {\mathcal {K}}(\varphi , A_t) N^{-\eta } \end{aligned}$$
  since both $\Vert \nabla q_1 \Psi \Vert$ and $\Vert q_1 \Psi \Vert$ are bounded uniformly in *N*. Since $q_2$ is a projector it follows that
  $$\begin{aligned} (141)&\le \frac{N^{2+\eta }}{N-1}\Vert \nabla _2 h_{\beta _1,\beta }(x_1-x_2)p_2\Vert _{\text {op}}^2 \Vert b_1 \mathbb {1}_{{{\mathcal {B}}_1^{(d)}}} \Psi \Vert ^2\\&\le C \frac{N^{2+\eta }}{N-1} \Vert \varphi \Vert _\infty ^2 \Vert \nabla h_{\beta _1,\beta }\Vert ^2 \Vert b_1 \mathbb {1}_{{{\mathcal {B}}_1^{(d)}}} \Psi \Vert ^2 \\&\le {\mathcal {K}}(\varphi , A_t) N^{\eta -1}\ln (N) \Vert \varphi \Vert _\infty ^2, \end{aligned}$$
  where we used Lemma 7.2 in the last step. Next, we estimate
  143 $$\begin{aligned} (142)&\le N^{2+\eta }\Vert p_2 \nabla _2 h_{\beta _1,\beta }(x_1-x_2)b_1 q_2 \mathbb {1}_{{{\mathcal {B}}_1^{(d)}}} \Psi \Vert ^2\ \nonumber \\&\le 2 N^{2+\eta }\Vert p_2 \nabla _2 h_{\beta _1,\beta }(x_1-x_2)b_1 q_2 \mathbb {1}_{\overline{{\mathcal {B}}}^{(d)}_{1}}\Psi \Vert ^2\ \end{aligned}$$
  144 $$\begin{aligned}&\quad +\,2 N^{2+\eta }\Vert p_2 \nabla _2 h_{\beta _1,\beta }(x_1-x_2)b_1 q_2 \Psi \Vert ^2. \end{aligned}$$
  The first term can be estimated as
  $$\begin{aligned} {}(143)&\le CN ^{2+ \eta } \Vert \nabla _2 h_{\beta _1,\beta }(x_1-x_2)b_1\Vert _{\text {op}}^2 \Vert \mathbb {1}_{\overline{{\mathcal {B}}}^{(d)}_{1}}\Psi \Vert ^2 \\&\le CN ^{2+ \eta } \Vert \nabla _2 h_{\beta _1,\beta }\Vert ^2 (\Vert \varphi \Vert ^2_\infty +\Vert \nabla \varphi \Vert ^2_\infty ) \Vert \mathbb {1}_{\overline{{\mathcal {B}}}^{(d)}_{1}}\Psi \Vert ^2 \\&\le {\mathcal {K}}(\varphi , A_t) N^{2+ \eta } N^{-2} \ln (N) N^{4 -2d} \\&\le {\mathcal {K}}(\varphi , A_t) N^{-2 + \eta } \ln (N), \end{aligned}$$
  for any $d \ge 3$. In the last line we used Lemma 7.4 (c) with $\epsilon =1$. The last term can be estimated as
  $$\begin{aligned} {}(144)&\le 2N^{2+\eta }\Vert p_2 h_{\beta _1,\beta }(x_1-x_2)b_1 \nabla _2q_2 \Psi \Vert ^2 \\&\quad +\, 2N^{2+\eta }\Vert |\varphi (x_2)\rangle \langle \nabla \varphi (x_2)| h_{\beta _1,\beta }(x_1-x_2)b_1 q_2 \Psi \Vert ^2\ \\&\le CN ^{2+\eta }\Vert p_2 h_{\beta _1,\beta }(x_1-x_2) \Vert _{\text {op}}^2 \Vert b_1\nabla _2q_2 \Psi \Vert ^2\ \\&\quad +\, CN ^{2+\eta } \Vert |\varphi (x_2)\rangle \langle \nabla \varphi (x_2)| h_{\beta _1,\beta }(x_1-x_2) \Vert _{\text {op}}^2 \Vert b_1 q_2 \Psi \Vert ^2\ \\&\le CN ^{2+\eta } \left( \Vert \nabla \varphi \Vert _\infty ^2+ \Vert \varphi \Vert _\infty ^2 \right) \Vert h_{\beta _1,\beta } \Vert ^2 (1+ \Vert \nabla \varphi \Vert ^2) \\&\le {\mathcal {K}}(\varphi , A_t) N^{\eta - 2\beta _1} \ln (N)^2. \end{aligned}$$
  Combining both estimates we obtain, for any $\beta >1/4$,
  $$\begin{aligned}&N\left| \langle \!\langle \mathbb {1}_{{{\mathcal {B}}_1^{(d)}}} \Psi , p_1p_2W_{\beta }(x_1-x_2)q_1q_2 \mathbb {1}_{{{\mathcal {B}}^{(d)}}_{1}}\Psi \rangle \!\rangle \right| \\&\quad \le \inf _{ \eta >0} \inf _{ 0<\beta _1 <1/4} \left( {\mathcal {K}}(\varphi , A_t) \left( \langle \!\langle \Psi , {\widehat{n}} \Psi \rangle \!\rangle + N^{-1 + 2 \beta _1} + N^{- \eta } \right. \right. \\&\qquad \left. \left. + N^{\eta -1} \ln (N) + N^{\eta -2 \beta _1} \ln (N) \right) \right) \\&\quad \le {\mathcal {K}}(\varphi , A_t) \left( \langle \!\langle \Psi , {\widehat{n}} \Psi \rangle \!\rangle + N^{-1/6} \ln (N) \right) . \end{aligned}$$
  where the last inequality comes from choosing $\eta =1/3$ and $\beta _1=1/4$. For $\Gamma = \Psi$, (b) can be estimated the same way, yielding the same bound.
(c) This follows from (a) and (b), using that $1-p_1p_2= q_1q_2+p_1q_2+q_1p_2$. $\square$

## Footnotes

1 We like to remark that it is well known that the convergence of $\gamma ^{(1)}_{\Psi }$ to $|\varphi \rangle \langle \varphi |$ in trace norm is equivalent to the respective convergence in operator norm since $|\varphi \rangle \langle \varphi |$ is a rank-1-projection, see Remark 1.4. in [51]. For other indicators of condensation and their relation we refer to [41].

2 Throughout the paper we use the notation $\langle \!\langle \cdot , \cdot \rangle \!\rangle = \langle \cdot , \cdot \rangle _{L^2_s({\mathbb {R}}^{2N}, {\mathbb {C}})}$ while $\langle \cdot , \cdot \rangle$ always refers to the scalar product of $L^2({\mathbb {R}}^2, {\mathbb {C}})$.

3 Even if the effect of the microscopic structure is negligible in the interaction, one should note that $\Psi _t$ is not close to a full product state $\prod _{k=1}^ N \varphi _t (x_k)$ in norm. For certain types of interactions, it has rigorously been shown that $\Psi _t$ can be approximated by a quasifree state satisfying a Bogoliubov-type dynamics. We refer to [5, 12, 23–25, 31, 35, 43–46] for the precise statements.

4 For an extensive introduction to the method we refer to [50].

5 It should be noted that the requirement $\alpha (\Psi _0,\varphi _0)\rightarrow 0$ defines conditions on the initial states $(\Psi _0,\varphi _0)$.

6 Note that a one particle ground state of negative energy decays exponentially, that is $\Psi _G (x) \le C_1 e^{-C_2 |x|}, C_1,C_2 >0$. Hence, (131) is well defined, although $F^{Z_n}_{\nu } \notin L^2( {\mathbb {R}}^2, {\mathbb {C}})$.
